# Proteomic landscape of seminal plasma associated with dairy bull fertility

**DOI:** 10.1038/s41598-018-34152-w

**Published:** 2018-11-05

**Authors:** A. G. A. Viana, A. M. A. Martins, A. H. Pontes, W. Fontes, M. S. Castro, C. A. O. Ricart, M. V. Sousa, A. Kaya, E. Topper, E. Memili, A. A. Moura

**Affiliations:** 1 Department of Animal Science, Federal University of Ceará, Fortaleza, Brazil; 2 Laboratory of Protein Chemistry and Biochemistry, University of Brasília, Brasília, Brazil; 3 Department of Reproduction and Artificial Insemination, Selcuk University, Konya, Turkey; 4 Alta Genetics Inc., Watertown, WI USA; 5 Department of Animal and Dairy Sciences, Mississippi State University, Starkville, USA

## Abstract

Male fertility is the ability of sperm to fertilize the egg and sustain embryo development. Several factors determine the fertilizing capacity of mammalian sperm, including those intrinsic to sperm and components of the seminal plasma. The present study analyzed the seminal fluid proteome of *Bos taurus* and potential associations between proteins and fertility scores. Mass spectrometry coupled with nano HPLC allowed the identification of 1,159 proteins in the dairy bull seminal plasma. There were 50 and 29 seminal proteins more abundant in high (HF) low fertility (LF) bulls, respectively. Based on multivariate analysis, C-type natriuretic peptide, TIMP-2, BSP5 and sulfhydryl oxidase indicated relationship with HF bulls. Clusterin, tissue factor pathway inhibitor 2, galectin-3-binding protein and 5′-nucleotidase were associated with LF bulls. Abundance of NAD(P)(+)-arginine ADP-ribosyltransferase, prosaposin and transmembrane protein 2 proteins had the highest positive correlations with fertility ranking. Quantities of vitamin D-binding protein, nucleotide exchange factor SIL1 and galectin-3-binding protein showed the highest negative correlations with fertility ranking. A fertility ranking score was calculated and the relationship with these proteins was significant (Spearman’s rho = 0.94). The present findings represent a major and novel contribution to the study of bovine seminal proteins. Indicators of fertility can be used to improve reproductive biotechnologies.

## Introduction

Reproduction efficiency of the male is one of the most important factors influencing sustainability of livestock as cryopreserved sperm from the elite bulls are widely distributed around the world by means of artificial insemination^1^. Male fertility is defined as the ability of sperm to fertilize the egg and sustain embryo development^2^ and accurate prediction of dairy bull fertility is still a significant challenge despite of the advances in genetic selection of dairy herds^1^. As well established, herd fertility is determined by female performance but aspects of male physiology are also important, as the AI industry still faces challenges related to bull subfertility^1,3^. Given that semen of dairy bulls are used to inseminate large number of cows, selection of high fertility bulls is crucial because even small increases in conception rates in the herds represent major revenues for the industry and farmers.

Several factors potentially determine the fertilizing capacity of mammalian sperm, including those that are intrinsic to sperm, such as DNA integrity^4^, RNA^5^, proteins^6^ and metabolites^7^. Sperm physiology and eventually their fertilizing capacity are also modulated by components of the milieu where they are maintained, the seminal plasma^3^. Seminal fluid is a composite secretion from the accessory sex glands and epididymides, mainly, and it contains organic and inorganic compounds, including proteins, lipids, ions and metabolites^8^. Proteins of the seminal plasma play vital roles in sperm protection^9^, capacitation^10^, acrosome reaction and sperm-egg binding, fertilization and initial embryonic development^11–13^. Given the importance of seminal plasma proteins, methods based on gel electrophoresis and mass spectrometry have allowed the identification of numerous classes of proteins in the bull seminal plasma^14–17^. More recently, the shotgun proteomic approach based on LC-MS/MS has been used for the study of protein mixtures^18^ as this technical strategy potentially allows more efficient identification of molecules in complex biological systems, such as the seminal plasma.

Studies have described statistical associations between components of reproductive fluids and fertility criteria of men^19,20^, boars^21^ and bulls^22–25^, among other species. These researchers used gel electrophoresis, N-terminal sequencing or SDS-PAGE coupled with mass spectrometry to compare and identify proteins of seminal plasma from males with different fertility status. Such studies certainly revealed important pieces of information about the composition of seminal fluid and potential molecular markers of fertility. Although representing remarkable achievements, gel-based strategies have limitations when used as the sole method to describe the whole proteome of a biological entity.

Also, several studies have used semen parameters to distinguish fertility phenotypic status in bulls^25^ and men as well^20^. However, such parameters are not reliable predictors of male fertility when compared to *in vivo* assays^26^. Working with dairy bulls as a model for fertility studies allows us to have access to an accurate database of sire conception rates calculated from hundreds of artificial inseminations. Thus, the present study was conducted to analyze the seminal plasma proteome of dairy sires (*Bos taurus*) using a label-free shotgun proteomics approach. We also investigated potential associations between seminal plasma proteins and fertility phenotype of the bulls.

## Results

### Seminal plasma proteome of Holstein bulls

**Figure 1:**
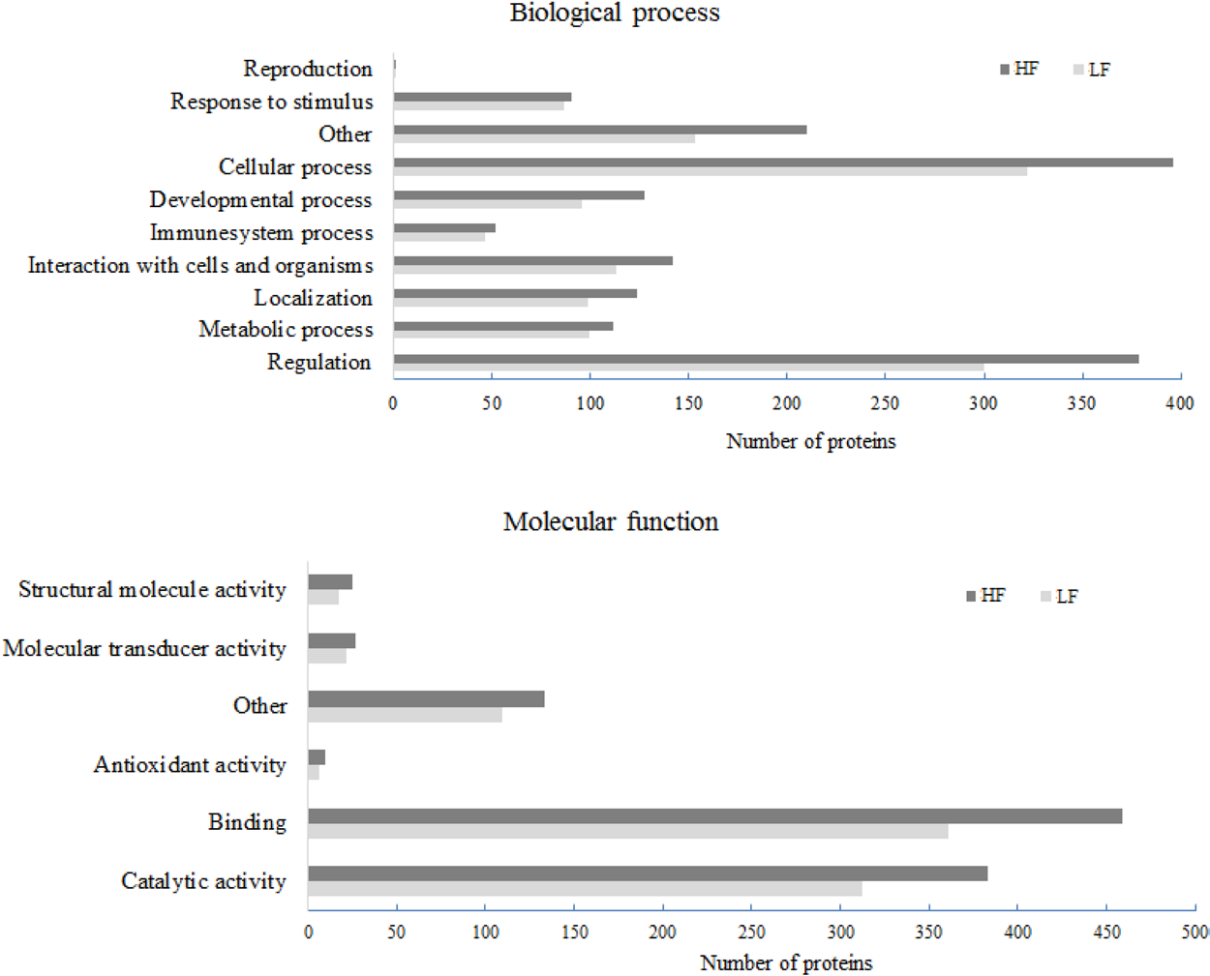
Gene ontology annotations of bull seminal plasma proteins based on biological process and molecular function. Protein data were analyzed using the software for researching annotations of proteins STRAP^27^. Gene ontology terms were obtained from UniProtKB database.

In the present study, 1,159 proteins were identified in the dairy bull seminal plasma, using mass spectrometry coupled with nano HPLC (Supplementary Table S1). Among all listed proteins, 765 were characterized according to UniProt database and 394 were still defined as non-characterized. Gene ontology terms related to biological process and molecular function of dairy bull seminal plasma proteins are presented in Fig. 1. The most important biological processes linked to the identified proteins were cellular process (24.3 and 24.5% in HF and LF bulls, respectively) followed by regulation (23.2 and 22.8% in HF and LF bulls, respectively) and interaction with cells and organisms (8.7 and 8.6% in HF and LF bulls, respectively). Molecular functions of bovine seminal proteins were mainly reported as binding (44.2 and 43.6% in HF and LF bulls, respectively) and catalytic activity (37 and 37.8% in HF and LF bulls, respectively).

### Comparison between protein profiles of seminal plasma from high and low fertility bulls

**Figure 2:**
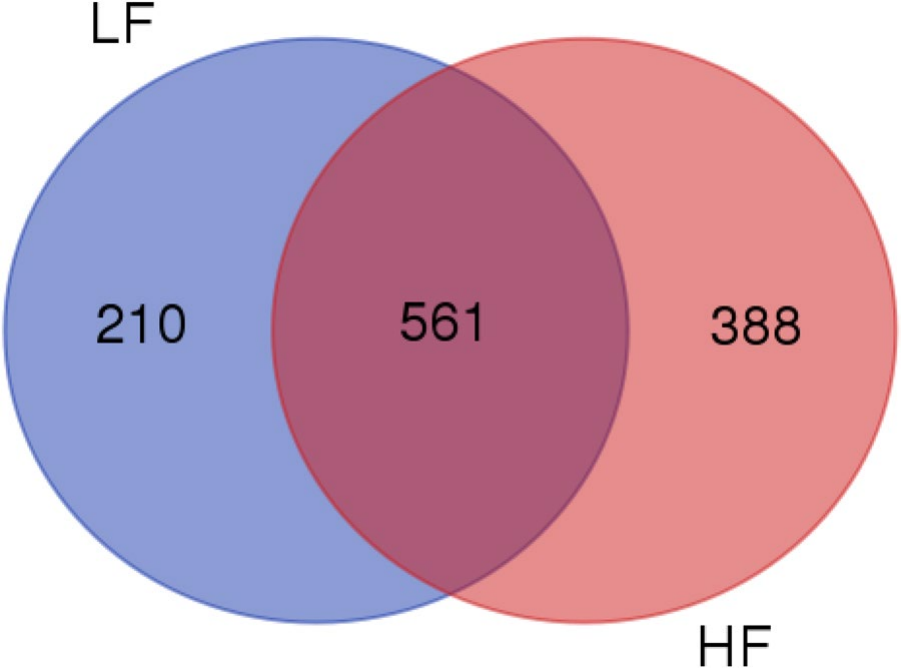
Proteomes of seminal plasma from bulls of high (HF, n = 5) and low (LF, n = 5) fertility scores. The Venn diagram was generated by the Bioinformatics & Evolutionary Genomics platform (http://bioinformatics.psb.ugent.be/webtools/Venn. From a total of 1,159 proteins, 949 were identified in HF group and 771 in LF. The intersection represents proteins (561) conserved to both HF and LF groups.

Of the 1,159 proteins identified in dairy bull seminal plasma, 949 were found in seminal plasma from HF bulls and 771 in LF sires, with 561 proteins (48.4%) common to both HF and LF phenotypes (Supplementary Table S1; Fig. 2). Thus, 388 proteins were exclusive to HF, while 210 were found in only LF sires. Among the proteins present in the seminal plasma of all bulls (561), there were 79 with different (p < 0.05) quantities in the groups of high and low fertility sires. Then, 50 proteins were more abundant in HF bulls and 29 proteins, in LF bulls (Table 1).

**Table 1:** Proteins of the seminal plasma differentially expressed in bulls with high (HF) and low (LF) fertility scores. Proteins were identified by DDA (data dependent acquisition) label-free mass spectrometry, Progenesis QI software and UniProt database.

Accession number	Description	Overexpression in	Peptide count	Unique peptides	Confidence score	Anova (p)
Q05927	5′-nucleotidase	LF	63	33	173.56	0.031
Q50HZ8	Acetyl-CoA carboxylase, type beta (Fragment)	HF	1	1	70.17	0.023
Q27984	Alpha1-antichymotrypsin isoform pHHK12 (Fragment)	LF	1	1	47.62	0.049
F1MVR5 G3N156	Anion exchange protein	LF	1	1	21.11	0.026
Q5EA01	Beta-1,4-glucuronyltransferase 1	LF	4	4	98,80	0.043
H7BWW2	Beta-hexosaminidase	LF	37	26	97.55	0.049
F1N619	Cadherin-1 (Fragment)	LF			174.00	0.012
P52193	Calreticulin	HF	5	5	61.38	0.049
P06833	Caltrin	HF	15	13	138.93	0.020
A6BML7	Carboxypeptidase	HF	15	15	39.83	0.008
Q17QK3	Carboxypeptidase Q	LF	1	1	47.79	0.047
A5PJF7	C-C motif chemokine	HF	3	3	339.39	0.009
F1N2J8	Chromosome 16 open reading frame 89	HF	6	6	75.18	0.032
F1MLR4	Ciliary neurotrophic factor receptor subunit alpha precursor	LF	6	6	121.84	0.033
P17697	Clusterin	LF	85	73	1233.10	0.027
A0A0F6QNP7	Complement component 3	LF	5	5	467.05	0.040
P81187	Complement factor B	LF	6	6	433.91	0.047
F1MC45	Complement factor H (Fragment)	LF	61	5	221.93	0.004
P55206	C-type natriuretic peptide	HF	46	44	488.24	0.032
A7MBJ5	Cullin-associated NEDD8-dissociated protein 1	LF	22	20	115.93	0.033
P81425	Dipeptidyl peptidase 4	LF	25	25	303.81	0.024
O18738	Dystroglycan	HF	2	2	94.94	0.011
E1BJV0	EH domain containing 4	LF	14	11	142.00	0.009
A6QR19	ENO2 protein	HF	1	1	33.46	0.042
P79345	Epididymal secretory protein E1	LF	29	29	123.57	0.031
A7E3W2	Galectin-3-binding protein	LF	23	22	313.46	0.031
E1BA29	Guanine nucleotide-binding protein G(q) subunit alhpa	LF	1	1	97.95	0.008
Q0P565	HD domain-containing protein 2	HF	1	1	70.52	0.005
Q76LV2	Heat shock protein HSP 90-alpha	HF	29	28	189.30	0.029
F1MNT3	Hormone-sensitive lipase	LF	10	8	52.37	0.004
Q7YS45	Hyaluronidase (Fragment)	LF	1	1	107.36	0.029
E1B748	Hypoxia up-regulated protein 1 precursor	HF	17	17	289.84	0,001
Q70IB2	Inactive ribonuclease-like protein 10	HF	8	8	57,69	0,029
Q95M12	Legumain	LF	8	8	138.49	0,022
Q9MYM4	Lysosomal alpha-glucosidase	LF	11	11	76.30	0,036
Q3SZI0	Mannose-6-phosphate isomerase	HF	6	6	84.25	0,039
A5D7D5	MATN2 protein	HF	4	4	50.06	0.022
E1BDF3	Matrilin 4	HF	20	20	149.27	0.017
P16368	Metalloproteinase inhibitor 2	HF	11	11	1071.83	0.034
Q9N282	MMP-9 (Fragment)	HF	1	1	33.60	0.014
Q1LZH9	N-acetylglucosamine-6-sulfatase	HF	9	9	62.80	0.040
E1BI74	NAD(P)(+)−arginine ADP-ribosyltransferase (Fragment)	HF	14	13	76.94	0.002
Q0IIH5	Nucleobindin 2	HF	26	25	290.90	0.021
Q32KV6	Nucleotide exchange factor SIL1	LF	5	5	57.08	0.029
A7MBI8	NUDT9 protein	LF	1	1	39.35	0.049
E1B818	Olfactomedin-like 2A-like	HF	6	6	163.47	0.001
Q9BGI2	Peroxiredoxin-4	HF	3	3	66.36	0.021
Q32KN6	Phosphoglycerate kinase	HF	35	27	100.42	0.045
Q28017	Platelet-activating factor acetylhydrolase	HF	61	59	1162.20	0.026
A1L555	Prosaposin	HF	35	3	120.78	0.007
P21856	Rab GDP dissociation inhibitor alpha	HF	9	4	64.47	0.033
Q0VCQ9	Reticulocalbin 2, EF-hand calcium binding domain	HF	9	9	65.81	0.020
Q0III8	RNASET2 protein (Fragment)	HF	6	6	26.70	0.042
A7MB70	Semaphorin-3C	HF	6	5	97.87	0.011
P81019	Seminal plasma protein BSP-30 kDa	HF	85	83	2706.25	0.005
P00669	Seminal ribonuclease	HF	92	79	1070.13	0.018
Q29443	Serotransferrin	LF	29	1	30.41	0.029
Q2HJF0	Serotransferrin-like	HF	33	4	208.39	0.002
Q862P3	Similar to cyclophilin B (Fragment)	HF	1	1	73.31	0.021
F1MJI3	SLIT-ROBO Rho GTPase activating protein 3	HF	2	1	20.89	0.036
Q4R0H2	Spermadhesin 2	LF	80	29	300.31	0.015
F1MHF1	ST6 beta-galactoside alpha-2,6-sialyltransferase 1	HF	13	13	138.21	0.024
P82292	Spermadhesin Z13	HF	53	4	36.67	0.038
A6QQA8	Sulfhydryl oxidase	HF	38	37	405.51	0.013
F1MJB6; A0JN68	Targeting protein for Xklp2	HF	1	1	20.33	0.024
Q32L40	T-complex protein 1 subunit alpha	HF	1	1	42.71	0.034
Q3ZBH0	T-complex protein 1 subunit beta	HF	18	18	58.09	0.021
Q7YRQ8	Tissue factor pathway inhibitor 2	LF	34	32	378.76	0,007
F1MNY2	Transmembrane protein 2	HF	1	1	22.47	0.010
Q3T077	Tubulin polymerization-promoting protein family member 2	HF	5	5	68.68	0,050
G3X861	Uncharacterized protein (Fragment)	HF	1	1	55.44	0.050
F1MY12	Uncharacterized protein (Fragment)	HF	2	2	91,12	0.010
E1BI55	Uncharacterized protein	HF	1	1	21.69	0.039
G5E5W7	Uncharacterized protein	HF	1	1	54.09	0.013
Q3MHN5	Vitamin D-binding protein	LF	1	1	70.54	0.021
Q32LB7	V-type proton ATPase subunit E 2	HF	1	1	31.85	0.044
P40682	V-type proton ATPase subunit S1	HF	1	1	60.26	0.025
Q3T0Z0	WAP four-disulfide core domain 2	HF	3	3	98.59	0.029
Q3ZCH5	Zinc-alpha-2-glycoprotein	LF	2	2	100.34	0.006

**Figure 3:**
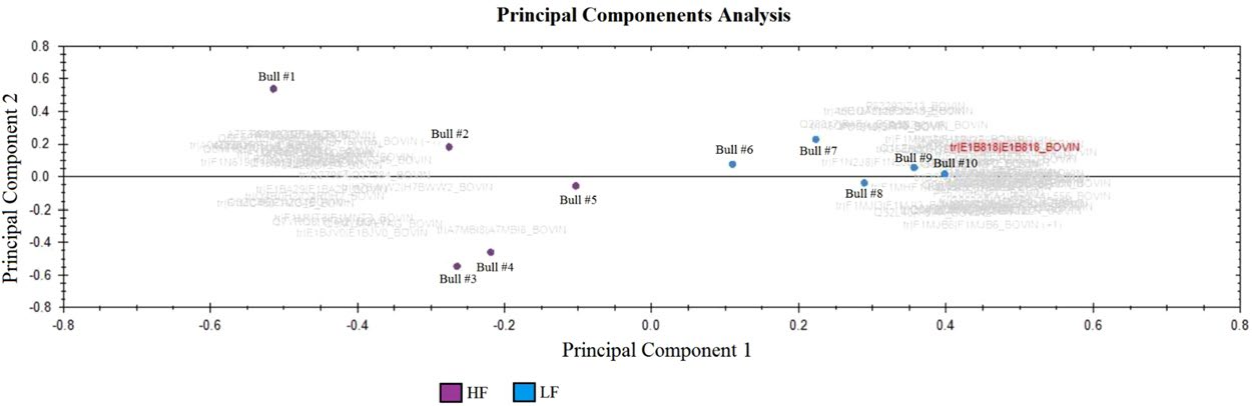
Principal component analysis (PCA) score plot associated with 79 seminal plasma proteins differentially expressed in high and low fertility bulls.

**Figure 4:**
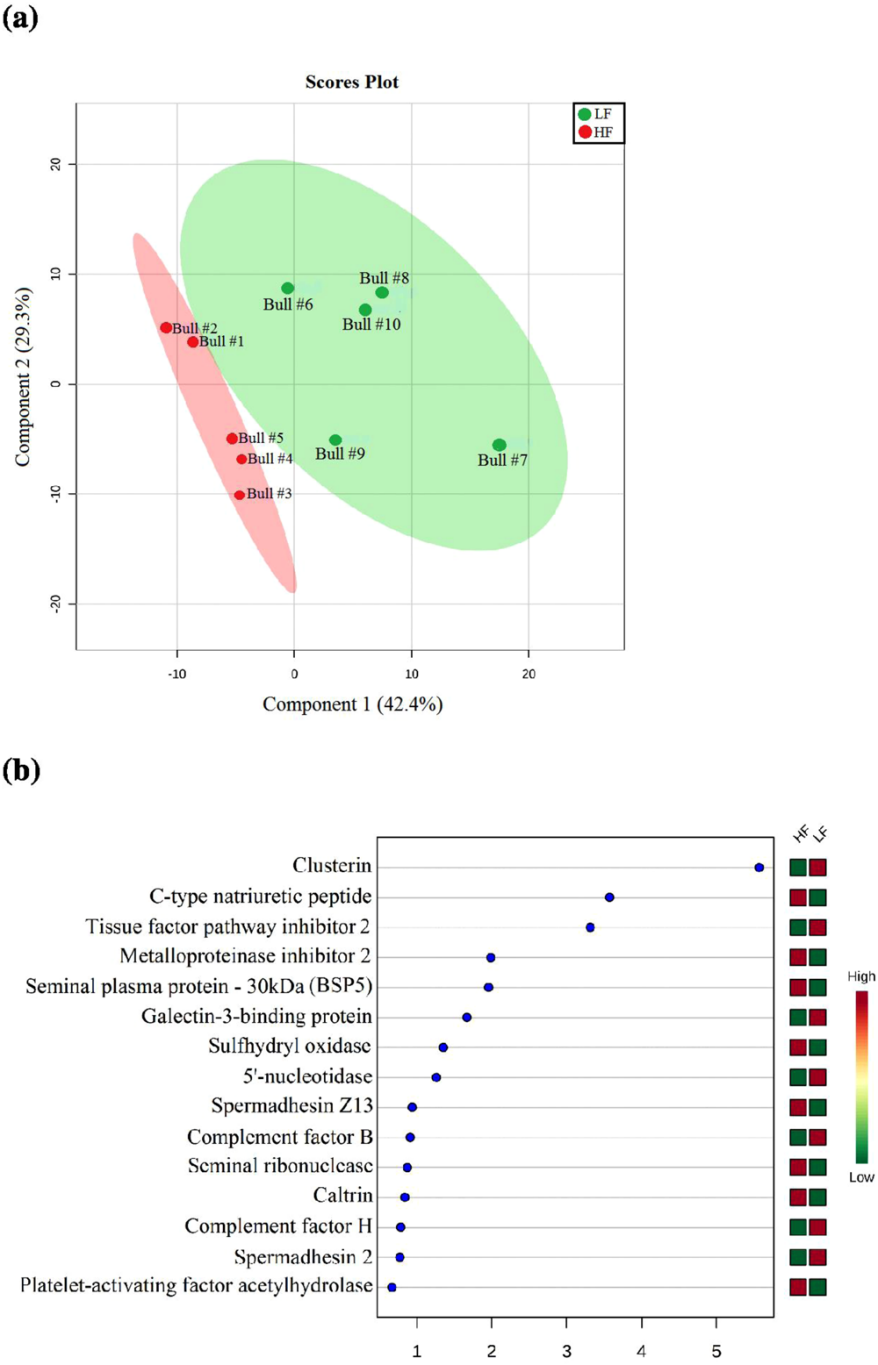
(**a**) Partial least-squares discriminant analysis (PLS-DA), showing the separation of groups of bulls with high and low fertility scores. The explained variances are shown in brackets. (**b**) Important features (proteins) based on Variable Importance in Projection (VIP) scores.

According to the plot based on the principal component analysis, there was a clear distribution of proteins with different quantities in high and low fertility bulls (Fig. 3). Based on multivariate analysis, a score plot of the two components with the highest variability (49.5 and 37.3%, data not shown) was performed (Fig. 4a) and eight proteins had VIP score greater than 1.5 (Fig. 4b), indicating meaningful contributions for definition of the fertility phenotype. Proteins contributing to definition of high fertility are C-type natriuretic peptide (NPPC), metalloproteinase inhibitor 2 (TIMP2), seminal plasma protein −30 kDa (BSP5) and sulfhydryl oxidase (QSOX1). On the other hand, clusterin (CLU), tissue factor pathway inhibitor 2 (TFPI2), galectin-3-binding protein and 5′-nucleotidase (NT5E) are the ones with meaningful contributions to low the fertility phenotype.

### Protein-based fertility rank score

**Figure 5:**
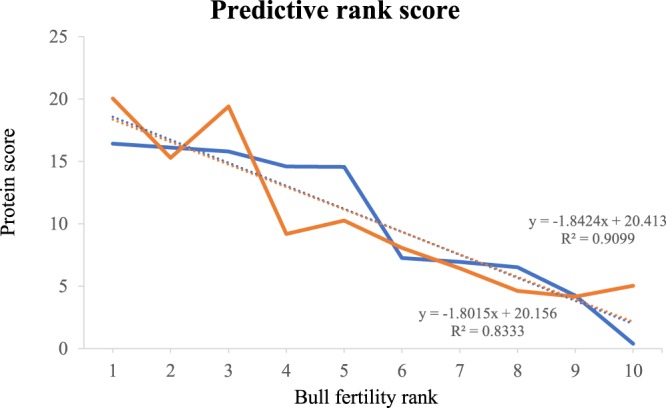
Predictive fertility rank score based on protein score (Y) and bull fertility rank (X). Protein score was obtained using normalized abundances of the six proteins with highest correlation with fertility rank. Bull fertility rank is shown from the highest (1) to the lowest value (10), as defined in Table 2. A predictive fertility rank score was significant with Spearman’s rho = 0.91 and Pearson’s correlation = 0.83. The blue line represents the conception rate difference from average (%) and the orange line represents the protein fertility score. The dotted line represents the linear regression for the respective (blue or orange) curve, showing the correlations between both scores, the conception rate and protein fertility score.

Given the 79 seminal plasma proteins with different abundances (p < 0.05) between the groups of high and low fertility bulls, we selected the six proteins showing the highest correlations with fertility ranking. NAD(P)(+)-arginine ADP-ribosyltransferase, prosaposin and transmembrane protein 2 had the highest positive correlations, while vitamin D-binding protein, nucleotide exchange factor SIL1 and galectin-3-binding protein showed the highest negative correlations with fertility of bulls. Using the normalized abundances of all these six proteins, it was possible to calculate a predictive fertility rank score with Spearman’s rho = 0.91 and Pearson’s correlation = 0.83 (Fig. 5).

### *In silico* analysis of protein-protein networks

**Figure 6:**
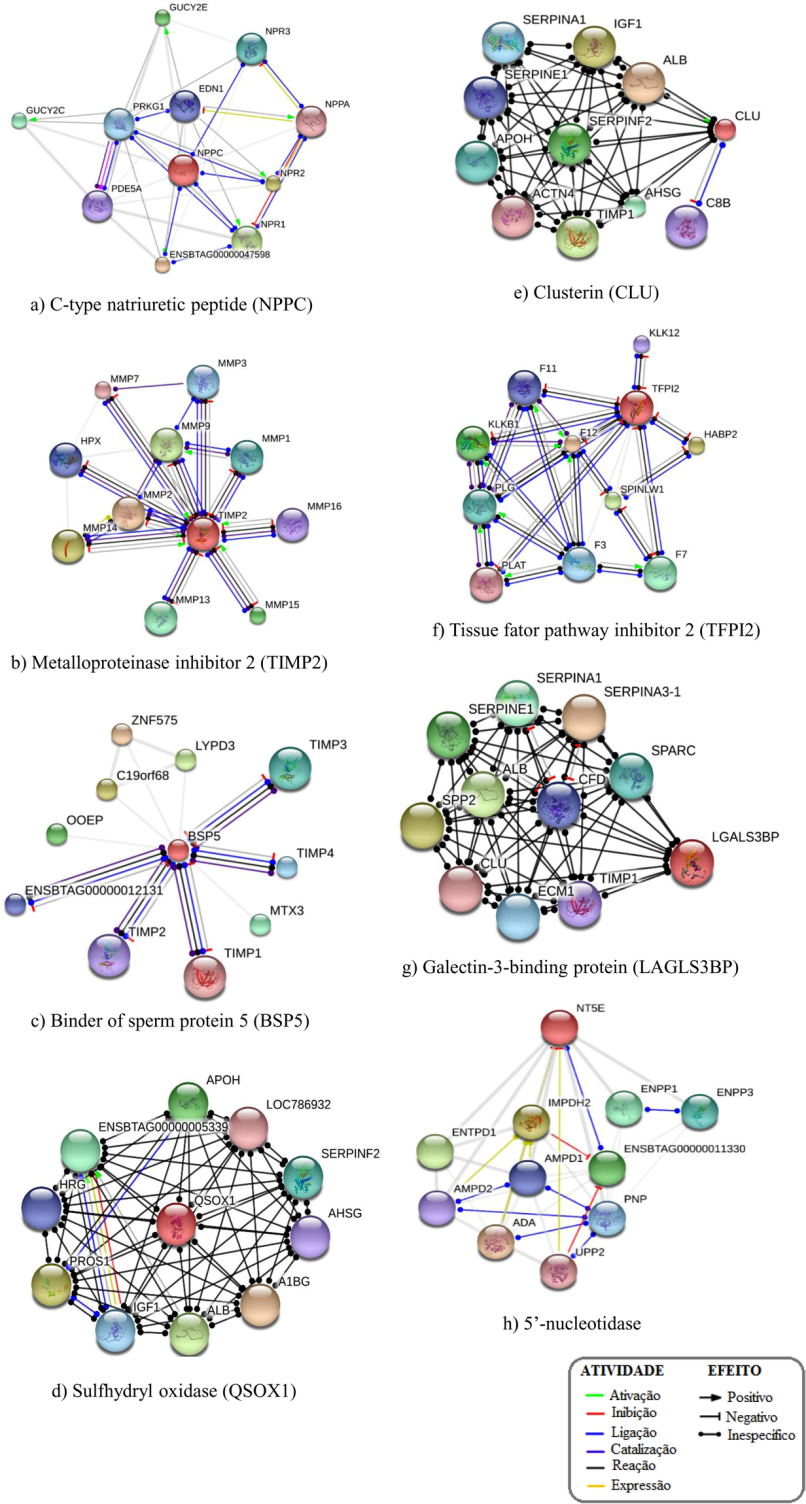
*In silico* analysis of protein-protein network as determined by STRING platform (http://string-db.org). Interactions were evaluated for seminal plasma proteins associated with VIP score >1.5: (**a**) C-type natriuretic peptide (NPPC); (**b**) Metalloproteinase inhibitor 2 (TIMP-2); (**c**) Binder of Sperm Protein 5 (BSP5); (**d**) Sulfhydryl oxidase (QSOX1); (**e**) Clusterin (CLU); (**f**) Tissue factor pathway inhibitor 2 (TFPI2); (**g**) Galectin-3-binding protein (LGAL3SBP); (**h**) 5′-nucleotidase (NT5E).

Seminal plasma proteins associated with VIP score >1.5 were NPPC, TIMP2, BSP5, QSOX1, CLU, TFPI2, galectin-3-binding protein and NT5E. Based on *in silico* analysis of protein-protein network, NPPC interacts with its receptors and with endothelin 1 (Fig. 6a). TIMP2 exhibits associations with several types of metalloproteinases (Fig. 6b) and BSP5 interacts with tissue inhibitor of metalloproteinases (Fig. 6c), as well as with oocyte-expressed protein homolog. QSOX1 interacts with glycoproteins (A1BG, HRG, AHSG), insulin-like growth factor 1 (IGF-1) and albumin (Fig. 6d). Clusterin shows links with albumin, IFG-1, alpha actinin 4, complement component C8 beta and protease inhibitors (Fig. 6e). TFPI2, in turn, interacts with proteins related to coagulation, plasminogen precursor and tissue type plasminogen activator (Fig. 6f). Galectin-3-binding protein interacts with protease inhibitors (serpins and TIMP1) and with clusterin, another protein overexpressed in low fertility animals with VIP score higher than 1.5 (Fig. 6g). Finally, NT5E interacts with enzymes such as deaminases and ectonucleotides (Fig. 6h).

## Discussion

In the present study, we used a label-free mass spectrometry approach to characterize the seminal plasma proteome of adult Holstein bulls. This strategy allowed the identification of 1,159 proteins and represents a major contribution to the understanding of seminal plasma composition in the *Bos taurus* species. Moreover, there were specific seminal proteins with different expression profiles in sires with contrasting fertility phenotypes determined *in vivo*.

The major biological processes (cellular process, regulation and interaction with cells) and molecular functions (binding and catalytic activity) of the seminal proteins related to their participation in events such as cell protection, sperm motility and capacitation, acrosome reaction, fertilization and embryonic development. Also, gene ontology terms related to biological process and molecular function were very similar in bulls of high and low fertility. The reason for this certainly relies on the fact that dairy bulls used in our study have been selected for decades by the AI companies and differences in fertility among them do not relate to general pattern of seminal plasma proteins, such as the pattern defined by gene ontology. Instead, differences in fertility phenotypes of those bulls are associated to very specific molecular aspects of seminal plasma, as we discuss below.

According to multivariate statistical analysis, proteins identified as more abundant in HF bulls were seminal plasma protein −30 kDa (BSP5), metalloproteinase inhibitor 2 (TIMP2), C-type natriuretic peptide (NPPC) and sulfhydryl oxidase (SQOX1). These proteins had VIP >1.5 and, thus, they are the best indicators of the high fertility phenotype of the bulls. BSP5 belongs to the Binder of Sperm Protein (BSP) family and, along with BSP1 and BSP3, represent around 60% of all proteins of bull seminal plasma^15,27,28^. BSPs are secreted by the accessory sex glands and, after ejaculation, bind to sperm^29^ and induce cholesterol and phospholipid efflux from the sperm membrane, an essential step for capacitation^30^. BSPs also mediate sperm interaction with the oviduct epithelium^31^ and BSP1 affects both fertilization and early development of bovine embryos *in vitro*^13^. *In silico* analysis points out to significant interactions between BSP5 and metalloproteinase inhibitors, such as TIMP-2, both with greater abundance in high fertility bulls. Furthermore, a link seems to exist between BSP5 and the oocyte-expressed protein homolog, which is located in subcortical cytoplasm of early embryos and play roles in cell divisions^32^, thereby supporting a putative role of BSP5 during fertilization.

Metalloproteinase inhibitors (TIMPs) modulate the activity of matrix metalloproteinases (MMPs), enzymes involved in remodeling of the extracellular matrix (ECM)^33^. The balance between MMPs and TIMPs is crucial during ECM remodeling^33^. Certain TIMPs have been suggested to play roles in sperm-egg fusion in the mouse^34^. TIMP-3 controls the degree of trophoblast implantation in the murine uterus^35^ and TIMP-2 content in bovine seminal plasma has a negative correlation with post-thaw sperm morphology and membrane stability^36^. Moreover, treatment of bull sperm with heparin binding proteins, a fertility-associated antigen and TIMP-2, increased pregnancy rates after artificial insemination^37^. Not only does TIMP-2 interact with several types of metalloproteinases but also with HPX, a protein that transports hemoglobin to the liver for breakdown and iron recovery. According to *in silico* analysis, TIMP-2 interacts with several types of metalloproteinases and, in fact, some MMPs are involved in reproductive events, such as angiogenesis, implantation and embryogenesis^38,39^.

NPPC belongs to a family of small peptides that participates in natriuresis and diuresis through vasodilatation^40^. Authors have described higher amounts of NPPC in reproductive tissues of male pigs when compared to other^41^. NPR-B (a NPPC receptor) is present in the acrosome and tail of human sperm and, thus, it is plausible that NPPC from seminal plasma binds to its receptor thereby stimulating intracellular cGMP and sperm motility^42^. *In silico* analysis showed interactions between NPPC, its receptors and guanylate cyclase, an enzyme involved in cGMP biosynthesis^42^. Also, NPPC interacts with natriuretic peptide A, which promotes trophoblasts implantation and artery remodeling in uterus^43^. QSOX1 plays a role in reduction of oxygen molecule to hydrogen peroxide, forming disulfide bonds in proteins and peptides^44^. In the male reproductive tract, QSOX1 protects spermatozoa structure and function by oxidizing sulfhydryl groups that could cause damage to the cell^45^. Several authors suggest that QSOX is crucial for sperm physiology and its dysregulation is associated with failures in spermatogenesis in hamsters^46^ and rats^47^. Based on *in silico* analysis, QSOX1 interacts with several types of glycoproteins present in the cellular membrane and with albumin, the most abundant protein of the cauda epididymal fluid in Holstein bulls^48^. Albumin protects sperm cells against harmful effects of lipid peroxides^49^ and acts during sperm capacitation^50^ and acrosome reaction^51^. QSOX1 also interacts with vascular endothelial growth factor A and with insulin growth factor 1, a protein that improves blastocyst rate formation^52^ in the bovine species.

Thus, seminal plasma proteins BSP5, TIMP2, NPPC and QSOX1 participate in important events related to reproduction, which explains, at least partially, their empirical associations with fertility. An earlier study described a quadratic relationship between BSP5 content in accessory sex gland fluid and fertility status of bulls^15^. This indicates that increasing amounts of BSP5 are beneficial but too much BSP5 in semen becomes detrimental to fertility. In fact, *in vitro* experiments confirm that BSPs are needed for proper sperm function but, when cells are exposed to high amounts of BSPs and for long periods of time, they excessively loose membrane cholesterol and phospholipids and become less viable^25,47^. Considering these facts, we suggest that the amount of BSP5 present in the bulls of our study was not sufficiently high to exert negative effects on fertility.

Seminal plasma clusterin (CLU), tissue factor pathway inhibitor 2 (TFPI2), galectin-3-binding protein and 5′-nucleotidase had VIP >1.5, indicating their significant contribution for definition of the low fertility phenotype of the dairy bulls. CLU is a chaperone and protects sperm against complement-mediated attack^53^ and against the effects of protein precipitation^54^. Clusterin contributes to removal of defective spermatozoa and is an indicator of poor semen quality in bulls^55^, rams^56^, men^57^, stallions^58^ and peccaries^59^. Also, seminal plasma CLU is inversely associated with the number of normal sperm in beef cattle^60^ and a positive association exists between abnormal morphology of sperm head and clusterin expression after scrotal insulation of Holstein bulls^55^. Based on *in silico* analysis, CLU interacts with a diverse cohort of molecules, some of which found in the reproductive tract and germ cells of bulls, such as serpins, albumin, TIMP, alpha-2-HS-glycoprotein. CLU also interacts with galectin-3 binding protein, another protein found at high levels in the seminal plasma of low fertility bulls, discussed below. Thus, there is sufficient experimental evidence in support of the inverse association between seminal plasma CLU and fertility of bulls.

TFPI-2 is a serine protease inhibitor also known as matrix-associated serine protease inhibitor (MSPI)^61^. It has been postulated that TFPI-2 is in fact one of the products of PP5 (placental protein 5) degradation^62^. PP5 is a placental glycoprotein associated with the coagulation and fibrinolytic system^63^ and PP5 plays a role in clotting and liquefaction mechanisms in human seminal plasma^64^. In fact, our *in silico* analysis indicates that TFPI-2 activity is linked to coagulation proteins, such as coagulation factors (F3, F7, F11 and F12), plasminogen precursor (PLG) and tissue-type plasminogen activator (PLAT). TFPI-2 also interacts with kallikreins, a group of proteins that convert kininogen into kinin, promoting increase in sperm motility^65^. However, further studies are still needed to confirm if TFPI-2 has a causal relation with low fertility in bulls. NT5E is a glycosylated enzyme already described in seminal plasma of bulls^66^ and participates in hydrolysis of AMP, stimulating sperm motility and sperm capacitation^67^. *In silico* analysis showed interactions of TFPI-2 with deaminases (ADA, AMPD1 and AMP D2), phosphorylases (ENTPD1, ENPP1, ENPP3, PNP and UPP2) and inosine-5′-monophosphate dehydrogenase 2 (IMPDH2). These enzymes that interact with TFPI-2 act through different intracellular signaling events that may lead to activation of sperm motility and capacitation. Galectin-3-binding protein is a member of beta-galactoside binding lectins expressed in various cells and tissues^68^ and this molecule has been previously found in epidydimal fluid of dairy bulls^48^. In humans, seminal galectin-3-binding protein plays multiple roles associated with semen liquefaction, sperm motility, angiogenesis in the female reproductive tract and as a pro-inflammatory agent^69^. *In silico* analysis detected an interaction between galectin-3 binding protein and clusterin, which is also over expressed in low fertility dairy bulls. Like clusterin, galectin-3-binding protein also interacts with albumin, a molecule involved in sperm capacitation and acrosome reaction, as we mentioned above. In addition, galectin-3 binding protein interacts with complement factor D (CFD), an activator of the immune system in the female reproductive tract^70^. As well known, seminal plasma components interact with the female reproductive tract, stimulating gene expression and the immune system, influencing fertility and embryo development^71^. Proteins from seminal plasma interact with endometrium epithelial cells, inducing or suppressing several mRNAs. This event causes synthesis of cytokines and chemokines that recruit immune cells from the blood to the endometrial lumen^72^. Besides cleaning the environment, such immune cells play roles in selection of the most competent sperm for fertilization^73^. Dendritic cells, a type of immune cells, carry seminal fluid antigens to the local lymph node activating Treg cell (regulatory T cells) population. Treg cells migrate, via blood, to the endometrium and promote endometrial receptivity for embryo implantation once the embryo expresses the same paternally derived antigens present in seminal plasma.

**Figure 7:**
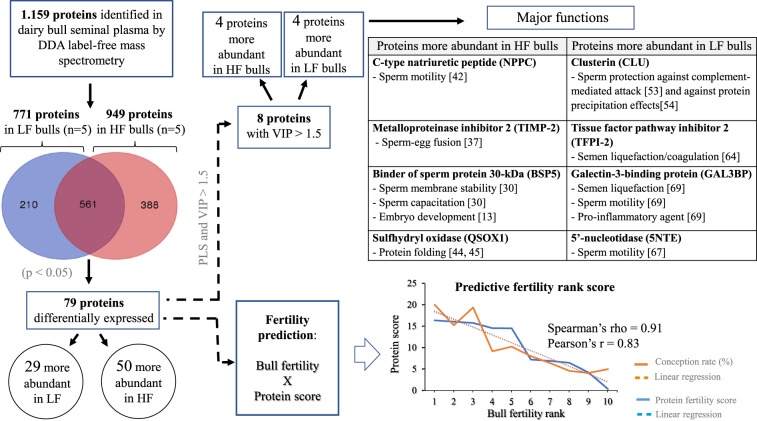
A graphical abstract showing the main findings of this study. Using DDA label-free mass spectrometry, 1,159 proteins were identified in dairy bull seminal plasma. Of the 1,159 proteins, 949 were found in seminal plasma from high fertility (HF, n = 5) bulls and 771 proteins in low fertility (LF, n = 5) sires. while 561 proteins were common to both HF and LF phenotypes. There were 50 and 29 seminal proteins more abundant in HF and LF bulls, respectively. Based on multivariate analyses, there were eight proteins with VIP score greater than 1.5 (Fig. 4b), indicating meaningful contributions of such proteins for definition of the fertility phenotype. Among them, four proteins were more abundant in either HF or LF bulls. DDA: dependent data acquired; PLS: partial least square; VIP: variable influence in projection.

In conclusion, the present study is a comprehensive overview of the proteome of bull seminal plasma. An approach based on DDA label-free mass spectrometry allowed the description of 1,159 proteins and this is, so far, the broadest inventory of the bovine seminal plasma proteome. At this point, we cannot precisely make inferences about the full protein composition of the seminal plasma of dairy bulls but it is certain that seminal fluid is a very complex milieu, containing components yet to be identified. Statistical analyses indicated eight proteins with significant contributions for definition of the fertility phenotype of dairy bulls. Moreover, we describe two distinct fertility indicators: a discriminator of high and low fertility bulls and a rank predictor. The entire approach used in our study and functional aspects of potential indicators of bull fertility are depicted in Fig. 7. Studies about the composition of seminal fluid set the foundations for the understanding of mechanisms regulating male fertility, which in turn has major economic impact for the industry and farmers. Also, definition of molecular indicators of bull fertility can be used to enhance reproductive biotechnologies for cattle.

## Materials and Methods

### Experimental design

Analysis of the seminal plasma proteome from Holstein bulls (*Bos taurus*) with contrasting *in vivo* fertility rates was conducted using high performance liquid chromatography combined with mass spectrometry. Computational biology as well as univariate and multivariate analyses were performed to compare the seminal proteome of high (HF) and low (LF) fertility dairy bulls and to detect molecular indicators for bull fertility. Then, we evaluated the correlation between normalized abundance of seminal proteins to create an equation of predictive fertility score.

### *In vivo* bull fertility and semen samples

Semen samples from ten Holstein bulls with reliable fertility phenotypes (Table 2) were provided by Alta Genetics (Watertown, WI, USA). Individual fertility scores of bulls used in the present study were calculated using Probit.F90 software, based on the average conception of at least 674 breeding outcomes per bull^6^. The population standard deviations were used as criteria to define bull fertility^74^ and, for the present study, high and low fertility sires differed from the mean by at least 1.3 standard deviations. Factors that influence fertility performance of sires (breeding event, environmental factors and herd management) were adjusted to determine reliable fertility scores using threshold models^69^.

**Table 2:** Fertility phenotypes of Holstein bulls. Bulls 1–5 are defined as high fertility (HF) and bulls 6–10, as low fertility (LF).

Bull #	Fertility status	Number of breedings	Conception rates (% difference from average)	Std of difference
1	HF	5293	5.42	2.0238
2	HF	825	5.1	1.9034
3	HF	2032	4.8	1.7931
4	HF	2487	3.59	1.3415
5	HF	5751	3.56	1.3304
6	LF	1604	−3.75	−1.4014
7	LF	2276	−4.06	−1.5159
8	LF	967	−4.49	−1.6762
9	LF	5603	−6.76	−2.5239
10	LF	674	−10.61	−3.9624

Semen from the five high and five low fertility bulls were collected using an artificial vagina and treated with a protease inhibitor, as reported before^24,74^. Right after semen collection, seminal plasma was subjected to a 10-min. centrifugation at 700 × *g* (4 °C). Afterwards, the resulting supernatant (seminal plasma) was transferred to a new tube and centrifuged again at 10,000 × *g* for 60 min., at 4 °C^27^. Following the second centrifugation, the supernatant was pipetted out into a cryotube, covered with Parafilm^®^M (Sigma-Aldrich, Darmstadt, Germany), then subjected to lyophilization using a Freeze Drier System (Labconco, Kansas City, MO, USA) (vacuum of 133 × 10^−3^ mBar, −40 °C). The samples were then stored at −80 °C for further analysis.

### Protein quantification, trypsinization and desalting

Lyophilized seminal plasma samples were suspended in 0.02 M TEAB and soluble protein content was quantified using Qubit^TM^ assay (Thermo Fisher Scientific, Waltham, MA, USA). Twenty-five micrograms of seminal plasma proteins were aliquoted in a microtube and dried in vacuum. To each sample, 15 µl of lysis buffer containing 8 M urea, 0.02 M TEAB and 0.5 M DTT (dithiothreitol) were added, followed by incubation at 55 °C and 400 rpm agitation (Eppendorf^®^ Thermomixer^®^ R, Sigma-Aldrich, Darmstadt, Germany) for 25 min. Further, as an alkylation process, a volume of IAA (iodoacetamide) was added to reach a final concentration of 0.014 M. The mixture was maintained at 21 °C and 400 rpm in the dark for 40 min. Prior to protein digestion, a volume of digestion buffer was added to reach a final concentration of 0.005 M DTT, 0.001 M CaCl_2_ and 0.02 M TEAB in a final volume of 75 µl. All samples were digested with trypsin (Promega, Fitchburg, WI, USA) with a 1/50 (w/w) enzyme/substrate ratio and incubated at 37 °C for 18 h. A solution of TFA (trifluoroacetic acid) was added to a final concentration of 1% to stop tryptic activity^75^.

Ten stage tip C18 columns were manually made to perform peptide desalting using Empore ^TM^ SPE disks (Sigma-Aldrich, Darmstadt, Germany), as previously described^76^. Briefly, to prepare a stage tip C18 membranes 100% methanol was added to the column at centrifuged at 1,000 × *g* for 3 min. The same procedure was repeated twice: first with a solution containing 80% acetonitrile and 0.5% acetic acid and second with 5% acetic acid. Finally, tryptic-digested samples were added to columns and centrifuged at 900 × *g* during 5 min, followed by washing twice with 0.5% acetonitrile at 1,000 × *g* for 3 min. To elute peptides, the columns were centrifuged at 600 × *g* for 3 min with increasing concentrations of acetonitrile (25% to 80%) with 0.5% acetic acid. Then, samples were again subjected to peptide quantification prior to mass spectrometry analysis (Qubit^TM^; Thermo Fisher, Waltham, MA, USA).

### Label-free mass spectrometry

Three micrograms of tryptic digested peptides from each sample were individually applied to a Dionex Ultimate 3,000 liquid chromatographer (Thermo Scientific, Waltham, MA, USA) for reversed phase nano-chromatography. The peptides were injected into a 2 cm × 100 µm trap-column containing C18, 5 µm particles (Dr. Maisch GmbH, Germany). The peptides were eluted from this column to another analytical one (32 cm × 75 µm) containing C18, 3 µm particles (Dr. Maisch GmbH, Germany) and finally eluted to the spectrometer’s ionization source. The elution gradient was composed of 0.1% formic acid in water (solvent A), and 0.1% formic acid in acetonitrile (solvent B), in a gradient of 2 to 35% solvent B for 170 min.

Samples were analyzed in positive DDA (data dependent acquisition) mode in a label-free mass spectrometric approach using an Orbitrap Elite instrument (Thermo Fisher, Waltham, MA, USA), as previously reported^77^. The eluted fractions generated MS1 spectra between 300–1,650 m/z with a resolution of 120,000 FWHM at 400 m/z. The twenty most abundant ions from MS1 with charges larger than two were automatically selected to fragmentation (MS2) by higher-energy collisional dissociation (HCD) with an automatic gain control (AGC) of 1 × 10^6^ and dynamic exclusion of 10 ppm for 90 s. HCD isolation window was set for 2.0 m/z, with 5 × 10^4^ AGC, normalized collision energy of 35% and threshold for detection of 3,000.

### Data analyses

MS1 spectra found in the chromatograms were aligned and, according to integrated intensity area from the XIC peaks generated by the respective ion, quantified using Progenesis QI software Nonlinear Dynamics (Waters, Milford, MA, USA). The protein identification was performed using Peaks software, which deduces sequences from the fragmentation information and searches in UniProt database. Protein identification information was inserted again in Progenesis QI program and combined with quantitative data generated previously.

Multivariate statistical analysis was performed using Progenesis QI software to evaluate differences in protein abundance in bulls of high and low fertility. Normalized abundances of proteins were plotted against fertility scores of each bull. A first statistical analysis was performed before protein identification to filter the MS1 features presenting ANOVA p-values < 0.05. Peaks 7.0 software was used with the fragmentation spectra and searched the *Bos taurus* Uniprot database, downloaded on 01/nov/2016. Parameters were set as following: precursor ion mass error tolerance of 10 ppm, MS/MS mass tolerance of 0.5 Da, carbamidomethylation of cysteine residues (fixed modification), deamidation and methionine oxidation (variable modifications). Trypsin was selected as the digestion enzyme, and up to two missed cleavage sites per peptide were allowed. The identified proteins were filtered at a rate of 1% for false discovery rate (FDR), and a minimum of 1 unique peptide per protein was required for identification. The protein input from Peaks were imported into the Progenesis QI software to generate quantitative data at the protein level. Multivariate Principal Component Analysis (PCA) was performed in Progenesis QI to evaluate protein abundances as related to phenotypes. Proteins were considered differentially abundant when presented p ≤ 0.05 after the ANOVA test at the protein level.

An additional multivariate analysis was carried out using MetaboAnalyst 3.0 (http://www.metaboanalyst.ca)^78^ considering the proteins significantly related to bull phenotypes. The protein dataset was normalized by sum, and Pareto-scaling was used to reduce relative importance of MS large values. Partial-Least Squares Discriminant Analysis (PLS-DA) was applied to differentiate classes in highly complex protein datasets, despite variability within each class. Variable Importance in Projection (VIP) based on the PLS-DA was used for the identification of biologically relevant features to categorize indicators of fertility. Then, variables with VIP >1.5 were considered important for group separation (high *vs* low fertility).

Normalized relative abundances of the regulated proteins were tested for correlation with the individual fertility scores using Spearman and Pearson correlation coefficient. The abundances of the three better positively correlated proteins and the three better negatively correlated proteins were used to calculate a rank score. Then, a protein-based fertility rank score was calculated based on a curve-fitting model of the protein abundances.

### Functional clustering and networking of bull seminal plasma proteins

Gene ontology (GO) analysis was carried out using STRAP software, gathering information about biological process and molecular function from UniProtKB and EBI databases. Moreover, *in silico* analysis of protein-protein network was performed using STRING (http://string-db.org) version 9.0 database^27^. Interactions were evaluated for seminal plasma proteins associated with VIP score >1.5.

## Electronic supplementary material

Supplementary Table S1
