# Supplementary material for: Proteomic landscape of seminal plasma associated with dairy bull fertility
Source: Sci Rep. 2018 Nov 5;8:16323. doi: 10.1038/s41598-018-34152-w (PMC6218504; doi:10.1038/s41598-018-34152-w)
Supplement: Supplementary file 1 — Supplementary Table S1 [file 41598_2018_34152_MOESM1_ESM.pdf]

# Proteomic landscape of seminal plasma associated with dairy bull fertility

Arabela Guedes de Azevedo Viana,

Aline Maria Araújo Martins,

Arthur Henriques Pontes,

Wagner Fontes,

Mariana de Souza Castro,

Carlos André Ornelas Ricart,

Marcelo Valle de Sousa,

Abdullah Kaya,

Einko Topper,

Erdogan Memili,

Arlindo de Alencar Araripe Noronha Moura

**Supplementary table S1.** Proteins of the bull seminal plasma as identified by DDA (data dependent acquisition) label-free mass spectrometry and tools of bioinformatics (Progenesis QI and UniProt database). The list of proteins includes those exclusively detected in high (HF) and low (LF) fertility bulls as well as those common (C) to both groups.

| Accession number (Uniprot) | Description | Group | Coverage (%) | #Peptides | #Unique | PTM | Avg. Mass (mDa) |
|---|---|---|---|---|---|---|---|
| tr\|A7Z057\|A7Z057_BOVIN | 14-3-3 protein gamma | HF | 17 | 4 | 1 | Y | 28303 |
| P68252\|1433G_BOVIN | 14-3-3 protein gamma | HF | 17 | 4 | 1 | Y | 28253 |
| Q0VC36\|1433S_BOVIN | 14-3-3 protein sigma | HF | 13 | 4 | 1 | Y | 27849 |
| Q3SZI4\|1433T_BOVIN | 14-3-3 protein theta | HF | 11 | 4 | 0 | N | 27764 |
| tr\|B0JYM5\|B0JYM5_BOVIN | 14-3-3 protein theta | HF | 11 | 4 | 0 | N | 27764 |
| P63103\|1433Z_BOVIN | 14-3-3 protein zeta/delta | C | 52 | 13 | 10 | Y | 27745 |
| A8YXY3\|SEP15_BOVIN | 15 kDa selenoprotein | HF | 32 | 3 | 3 | Y | 17975 |
| tr\|F1MLV1\|F1MLV1_BOVIN | 26S protease regulatory subunit 10B | HF | 4 | 1 | 1 | N | 44173 |
| Q2KIW6\|PRS10_BOVIN | 26S protease regulatory subunit 10B | HF | 4 | 1 | 1 | N | 44074 |
| P56701\|PSMD2_BOVIN | 26S proteasome non-ATPase regulatory subunit 2 | HF | 2 | 1 | 1 | Y | 100258 |
| Q3ZBZ1\|CAB45_BOVIN | 45 kDa calcium-binding protein | C | 70 | 18 | 18 | Y | 41106 |
| tr\|F1N2L9\|F1N2L9_BOVIN | 4-trimethylaminobutyraldehyde dehydrogenase | C | 2 | 1 | 1 | N | 53991 |
| Q2KJH9\|AL9A1_BOVIN | 4-trimethylaminobutyraldehyde dehydrogenase | C | 2 | 1 | 1 | N | 53977 |
| Q05927\|5NTD_BOVIN | 5'-nucleotidase | C | 61 | 31 | 16 | Y | 62966 |
| tr\|G3MZC1\|G3MZC1_BOVIN | 5'-nucleotidase (Fragment) | HF | 54 | 18 | 3 | Y | 35982 |
| Q75WB5\|OPLA_BOVIN | 5-oxoprolinase | LF | 1 | 1 | 1 | Y | 137465 |
| tr\|F1ME97\|F1ME97_BOVIN | 5-oxoprolinase | LF | 1 | 1 | 1 | Y | 137354 |
| tr\|Q3ZCI4\|Q3ZCI4_BOVIN | 6-phosphogluconate dehydrogenase decarboxylating | HF | 2 | 1 | 1 | N | 53077 |
| tr\|F1MKH8\|F1MKH8_BOVIN | 72 kDa type IV collagenase | C | 10 | 2 | 2 | N | 73848 |
| Q0VCX2\|GRP78_BOVIN | 78 kDa glucose-regulated protein | C | 20 | 11 | 9 | N | 72400 |
| tr\|F1MM80\|F1MM80_BOVIN | Abnormal spindle-like microcephaly-associated protein homolog | C | 0 | 1 | 1 | Y | 405871 |

| | | | | | | | |
|---|---|---|---|---|---|---|---|
| P62285\|ASPM_BOVIN | Abnormal spindle-like microcephaly-associated protein homolog (Fragment) | HF | 0 | 1 | 1 | Y | 395924 |
| tr\|A0JND7\|A0JND7_BOVIN | ABO blood group (Transferase A alpha 1-3-N-acetylgalactosaminyltransferase | LF | 9 | 2 | 2 | N | 41926 |
| P23795\|ACES_BOVIN | Acetylcholinesterase | C | 2 | 3 | 0 | N | 67664 |
| Q17QB3\|ASAH1_BOVIN | Acid ceramidase | LF | 21 | 6 | 6 | N | 44965 |
| tr\|G3M8V1\|G3M8V1_BOVIN | Acid phosphatase type 5 | C | 16 | 4 | 4 | Y | 38237 |
| Q3ZC91\|ASM3A_BOVIN | Acid sphingomyelinase-like phosphodiesterase 3a | C | 7 | 2 | 2 | N | 51221 |
| tr\|F1MUI5\|F1MUI5_BOVIN | Acid sphingomyelinase-like phosphodiesterase 3a | C | 7 | 2 | 2 | N | 51175 |
| tr\|P79343\|P79343_BOVIN | Acrosin | C | 8 | 3 | 3 | Y | 41722 |
| P01000\|IAC1_BOVIN | Acrosin inhibitor 1 | C | 60 | 6 | 6 | Y | 7379 |
| tr\|Q32KR2\|Q32KR2_BOVIN | Acrosomal vesicle protein 1 | LF | 14 | 3 | 3 | Y | 28953 |
| tr\|A4IFM8\|A4IFM8_BOVIN | Actin alpha 1 skeletal muscle | HF | 32 | 16 | 1 | Y | 42023 |
| Q3ZC07\|ACTC_BOVIN | Actin alpha cardiac muscle 1 | C | 32 | 17 | 0 | Y | 42019 |
| P68138\|ACTS_BOVIN | Actin alpha skeletal muscle | C | 32 | 17 | 0 | Y | 42051 |
| P60712\|ACTB_BOVIN | Actin cytoplasmic 1 | C | 75 | 26 | 11 | Y | 41737 |
| P63258\|ACTG_BOVIN | Actin cytoplasmic 2 | C | 75 | 26 | 11 | Y | 41793 |
| A6QQV9\|AF1L1_BOVIN | Actin filament-associated protein 1-like 1 | HF | 2 | 2 | 2 | Y | 85931 |
| E1BNG3\|ASCC3_BOVIN | Activating signal cointegrator 1 complex subunit 3 | HF | 1 | 2 | 1 | N | 250763 |
| tr\|Q3MHY9\|Q3MHY9_BOVIN | ACTR1A protein (Fragment) | HF | 16 | 4 | 4 | N | 41463 |
| P07107\|ACBP_BOVIN | Acyl-CoA-binding protein | C | 61 | 6 | 6 | Y | 10044 |
| tr\|Q2NKZ3\|Q2NKZ3_BOVIN | ADAM metallopeptidase domain 32 | C | 7 | 4 | 4 | Y | 82546 |
| P00570\|KAD1_BOVIN | Adenylate kinase isoenzyme 1 | C | 74 | 13 | 13 | Y | 21664 |
| O62827\|ADML_BOVIN | ADM | C | 49 | 13 | 13 | Y | 20963 |
| tr\|B0JYQ0\|B0JYQ0_BOVIN | ALB protein | LF | 64 | 47 | 1 | Y | 69294 |
| tr\|Q5E962\|Q5E962_BOVIN | Aldo-keto reductase family 1 member B1 | C | 76 | 18 | 16 | Y | 36050 |
| Q5EA79\|GALM_BOVIN | Aldose 1-epimerase | HF | 5 | 1 | 1 | N | 37614 |
| P16116\|ALDR_BOVIN | Aldose reductase | C | 76 | 18 | 16 | Y | 35919 |
| tr\|A4FUG6\|A4FUG6_BOVIN | ALG2 protein | LF | 2 | 1 | 1 | Y | 46831 |
| P09487\|PPBT_BOVIN | Alkaline phosphatase tissue-nonspecific isozyme | C | 7 | 3 | 3 | N | 57193 |
| tr\|Q28921\|Q28921_BOVIN | Alpha 1-antichymotrypsin (Fragment) | HF | 6 | 1 | 1 | N | 28571 |
| tr\|Q5GN72\|Q5GN72_BOVIN | Alpha-1-acid glycoprotein | C | 7 | 1 | 1 | N | 23158 |
| Q3SZR3\|A1AG_BOVIN | Alpha-1-acid glycoprotein | C | 7 | 1 | 1 | N | 23182 |

| | | | | | | | |
|---|---|---|---|---|---|---|---|
| tr\|Q27983\|Q27983_BOVIN | Alpha1-antichymotrypsin isoform pHHK11 (Fragment) | HF | 7 | 1 | 1 | N | 22711 |
| P34955\|A1AT_BOVIN | Alpha-1-antiproteinase | C | 4 | 1 | 1 | N | 46104 |
| Q2KJF1\|A1BG_BOVIN | Alpha-1B-glycoprotein | LF | 10 | 3 | 3 | Y | 53554 |
| P28800\|A2AP_BOVIN | Alpha-2-antiplasmin | C | 3 | 1 | 1 | N | 54711 |
| P12763\|FETUA_BOVIN | Alpha-2-HS-glycoprotein | LF | 9 | 2 | 2 | Y | 38419 |
| tr\|B0JYN6\|B0JYN6_BOVIN | Alpha-2-HS-glycoprotein | LF | 9 | 2 | 2 | Y | 38419 |
| Q7SIH1\|A2MG_BOVIN | Alpha-2-macroglobulin | C | 18 | 20 | 19 | Y | 167575 |
| Q3B7N2\|ACTN1_BOVIN | Alpha-actinin-1 | HF | 4 | 3 | 2 | Y | 102980 |
| Q9XSJ4\|ENOA_BOVIN | Alpha-enolase | C | 18 | 6 | 6 | N | 47326 |
| tr\|F1MB08\|F1MB08_BOVIN | Alpha-enolase | LF | 12 | 3 | 3 | N | 47284 |
| tr\|G5E5M8\|G5E5M8_BOVIN | Alpha-mannosidase | C | 11 | 10 | 4 | Y | 115130 |
| tr\|E1BHP0\|E1BHP0_BOVIN | Alpha-mannosidase | C | 44 | 32 | 22 | Y | 115228 |
| tr\|F1MWT0\|F1MWT0_BOVIN | Alpha-mannosidase | C | 42 | 27 | 6 | Y | 115861 |
| tr\|A8YXY9\|A8YXY9_BOVIN | Alpha-mannosidase | HF | 48 | 21 | 1 | Y | 85396 |
| Q58DH9\|NAGAB_BOVIN | Alpha-N-acetylgalactosaminidase | LF | 3 | 1 | 1 | N | 46533 |
| tr\|Q1RMM9\|Q1RMM9_BOVIN | Alpha-N-acetylgalactosaminidase | LF | 3 | 1 | 1 | N | 46561 |
| tr\|Q08E54\|Q08E54_BOVIN | Amyloid beta (A4) protein | C | 14 | 5 | 5 | Y | 78597 |
| tr\|A6H6X2\|A6H6X2_BOVIN | ANG2 protein | C | 43 | 5 | 5 | Y | 17155 |
| tr\|Q2NKV1\|Q2NKV1_BOVIN | Angiogenin  ribonuclease  RNase A family  5 | C | 56 | 10 | 10 | Y | 17004 |
| P10152\|ANG1_BOVIN | Angiogenin-1 | C | 56 | 10 | 10 | Y | 16970 |
| P80929\|ANG2_BOVIN | Angiogenin-2 | C | 51 | 5 | 5 | Y | 14522 |
| tr\|Q9BDG1\|Q9BDG1_BOVIN | Angiotensin I converting enzyme (Fragment) | C | 73 | 6 | 3 | Y | 14326 |
| tr\|F1MQJ0\|F1MQJ0_BOVIN | Angiotensin-converting enzyme (Fragment) | C | 30 | 39 | 34 | Y | 141241 |
| P15497\|APOA1_BOVIN | Apolipoprotein A-I | C | 32 | 7 | 7 | Y | 30276 |
| tr\|V6F9A2\|V6F9A2_BOVIN | Apolipoprotein A-I | C | 32 | 7 | 7 | Y | 30276 |
| P81644\|APOA2_BOVIN | Apolipoprotein A-II | C | 16 | 1 | 1 | N | 11202 |
| Q08DD1\|ARSA_BOVIN | Arylsulfatase A | C | 16 | 3 | 3 | N | 53807 |
| Q148F3\|ARSK_BOVIN | Arylsulfatase K | C | 10 | 3 | 3 | N | 61361 |
| Q2HJH1\|DNPEP_BOVIN | Aspartyl aminopeptidase | C | 41 | 11 | 11 | Y | 51828 |
| tr\|Q3MHZ3\|Q3MHZ3_BOVIN | ATP6AP1 protein (Fragment) | C | 22 | 6 | 6 | Y | 51519 |
| tr\|A7E3T8\|A7E3T8_BOVIN | ATP-binding cassette  sub-family G  member 2 | HF | 3 | 1 | 1 | Y | 73078 |
| tr\|B2D1N9\|B2D1N9_BOVIN | ATP-binding cassette sub-family G member 2 | HF | 3 | 1 | 1 | Y | 73097 |
| Q32PF2\|ACLY_BOVIN | ATP-citrate synthase | HF | 1 | 1 | 1 | N | 119789 |

| | | | | | | | |
|---|---|---|---|---|---|---|---|
| P17453\|BPI_BOVIN | Bactericidal permeability-increasing protein | LF | 2 | 1 | 1 | N | 53442 |
| tr\|F1MQ17\|F1MQ17_BOVIN | Bactericidal permeability-increasing protein (Fragment) | LF | 2 | 1 | 1 | N | 49002 |
| tr\|Q3ZBX0\|Q3ZBX0_BOVIN | Basigin | LF | 5 | 1 | 1 | N | 29773 |
| Q5EA01\|B4GA1_BOVIN | Beta-1 4-glucuronyltransferase 1 | LF | 18 | 3 | 3 | N | 47231 |
| P01888\|B2MG_BOVIN | Beta-2-microglobulin | C | 58 | 8 | 8 | Y | 13677 |
| tr\|A7LM96\|A7LM96_BOVIN | Beta-defensin | C | 15 | 1 | 1 | Y | 9310 |
| tr\|G3N236\|G3N236_BOVIN | Beta-defensin (Fragment) | C | 16 | 1 | 1 | Y | 10056 |
| tr\|G8CY17\|G8CY17_BOVIN | Beta-defensin (Fragment) | C | 21 | 4 | 4 | Y | 10785 |
| tr\|G8CY19\|G8CY19_BOVIN | Beta-defensin (Fragment) | C | 31 | 3 | 3 | Y | 11397 |
| tr\|G8CY20\|G8CY20_BOVIN | Beta-defensin (Fragment) | C | 10 | 2 | 2 | Y | 9237 |
| tr\|F1MBP7\|F1MBP7_BOVIN | Beta-defensin 10 | C | 40 | 3 | 3 | Y | 6954 |
| P46168\|DFB10_BOVIN | Beta-defensin 10 | C | 40 | 3 | 3 | Y | 6928 |
| P46169\|DFB11_BOVIN | Beta-defensin 11 | HF | 33 | 3 | 1 | Y | 6507 |
| Q32P86\|DB119_BOVIN | Beta-defensin 119 | C | 28 | 2 | 2 | Y | 9805 |
| P46170\|DFB12_BOVIN | Beta-defensin 12 | HF | 53 | 3 | 1 | Y | 4106 |
| tr\|G8CY12\|G8CY12_BOVIN | Beta-defensin 126 (Fragment) | C | 27 | 3 | 3 | Y | 9374 |
| tr\|G8CY08\|G8CY08_BOVIN | Beta-defensin 126 (Fragment) | C | 7 | 1 | 1 | N | 11183 |
| tr\|G8CY14\|G8CY14_BOVIN | Beta-defensin 129 (Fragment) | C | 31 | 7 | 6 | Y | 17856 |
| P46171\|DFB13_BOVIN | Beta-defensin 13 | HF | 48 | 3 | 1 | Y | 4450 |
| tr\|G8CY09\|G8CY09_BOVIN | Beta-defensin 132 | HF | 8 | 1 | 1 | Y | 9337 |
| tr\|Q5W5H4\|Q5W5H4_BOVIN | Beta-defensin 405 (Fragment) | C | 60 | 3 | 3 | Y | 4775 |
| O18815\|DFBC7_BOVIN | Beta-defensin C7 (Fragment) | C | 38 | 4 | 2 | Y | 5650 |
| tr\|A5D7U5\|A5D7U5_BOVIN | Beta-galactosidase | C | 40 | 18 | 18 | Y | 73475 |
| tr\|F1MUF4\|F1MUF4_BOVIN | Beta-galactosidase | C | 40 | 18 | 18 | Y | 73475 |
| tr\|F1MDX6\|F1MDX6_BOVIN | Beta-galactosidase (Fragment) | C | 7 | 2 | 1 | N | 75483 |
| tr\|O18974\|O18974_BOVIN | Beta-galactoside alpha-2 6-sialyltransferase | C | 38 | 14 | 14 | Y | 46246 |
| tr\|A3KMY8\|A3KMY8_BOVIN | Beta-glucuronidase | C | 38 | 19 | 17 | Y | 74470 |
| tr\|H7BWW2\|H7BWW2_BOVIN | Beta-hexosaminidase | C | 54 | 28 | 19 | Y | 61262 |
| tr\|E1B9E8\|E1B9E8_BOVIN | Beta-hexosaminidase | C | 33 | 19 | 10 | Y | 62467 |
| Q0V8R6\|HEXA_BOVIN | Beta-hexosaminidase subunit alpha | C | 6 | 2 | 2 | N | 60353 |
| Q5I597\|BHMT1_BOVIN | Betaine--homocysteine S-methyltransferase 1 | HF | 2 | 1 | 1 | N | 44878 |
| Q29444\|MANBA_BOVIN | Beta-mannosidase | C | 59 | 38 | 36 | Y | 101176 |
| tr\|A6QLB0\|A6QLB0_BOVIN | Beta-mannosidase | C | 59 | 38 | 36 | Y | 101124 |

| | | | | | | | |
|---|---|---|---|---|---|---|---|
| P13600\|NGF_BOVIN | Beta-nerve growth factor | C | 48 | 18 | 18 | Y | 26669 |
| tr\|F1MJM4\|F1MJM4_BOVIN | Biotinidase | LF | 5 | 3 | 3 | N | 58310 |
| A6QQ07\|BTD_BOVIN | Biotinidase | LF | 5 | 3 | 3 | N | 58353 |
| tr\|A5D7K0\|A5D7K0_BOVIN | BLVRA protein | LF | 2 | 1 | 1 | N | 33644 |
| Q2KJH1\|BMP4_BOVIN | Bone morphogenetic protein 4 | C | 13 | 3 | 3 | Y | 46626 |
| tr\|E5DCU8\|E5DCU8_BOVIN | Bone morphogenetic protein 4 (Fragment) | C | 14 | 3 | 3 | Y | 42751 |
| tr\|Q5I4I9\|Q5I4I9_BOVIN | Bone morphogenetic protein 4 (Fragment) | C | 14 | 3 | 3 | Y | 44544 |
| Q8SPU5\|BPIA1_BOVIN | BPI fold-containing family A member 1 | C | 11 | 2 | 2 | N | 26576 |
| Q8SPF8\|BPIB1_BOVIN | BPI fold-containing family B member 1 | C | 52 | 19 | 19 | Y | 51700 |
| tr\|F1MLW2\|F1MLW2_BOVIN | BPI fold-containing family B member 1 | C | 52 | 19 | 19 | Y | 51716 |
| P80724\|BASP1_BOVIN | Brain acid soluble protein 1 | C | 57 | 7 | 7 | N | 23011 |
| P39873\|RNBR_BOVIN | Brain ribonuclease | C | 45 | 14 | 4 | Y | 18450 |
| Q95106\|BDNF_BOVIN | Brain-derived neurotrophic factor | HF | 3 | 1 | 1 | N | 28174 |
| tr\|F1N7Q0\|F1N7Q0_BOVIN | BTB (POZ) domain containing 12 | HF | 1 | 2 | 0 | N | 197631 |
| tr\|O46625\|O46625_BOVIN | BTrappin-2 protein (Fragment) | C | 51 | 6 | 4 | Y | 14324 |
| tr\|A6H758\|A6H758_BOVIN | C11H9ORF9 protein | C | 6 | 1 | 1 | N | 25006 |
| Q3SX46\|C1GLC_BOVIN | C1GALT1-specific chaperone 1 | C | 19 | 4 | 4 | Y | 36441 |
| tr\|A5PJL8\|A5PJL8_BOVIN | C1QTNF5 protein | C | 56 | 11 | 10 | Y | 25349 |
| Q6R8F2\|CADH1_BOVIN | Cadherin-1 | C | 6 | 6 | 6 | Y | 97938 |
| tr\|F1N619\|F1N619_BOVIN | Cadherin-1 (Fragment) | C | 6 | 6 | 6 | Y | 91860 |
| tr\|F1MGE7\|F1MGE7_BOVIN | Calcium-transporting ATPase | LF | 1 | 1 | 1 | N | 109266 |
| tr\|E1BMQ6\|E1BMQ6_BOVIN | Calcium-transporting ATPase | C | 1 | 1 | 1 | N | 109321 |
| P62157\|CALM_BOVIN | Calmodulin | C | 36 | 2 | 2 | N | 16838 |
| tr\|F1MLH6\|F1MLH6_BOVIN | Calmodulin | C | 36 | 2 | 2 | N | 16847 |
| tr\|A5D7J6\|A5D7J6_BOVIN | CALR protein | HF | 30 | 6 | 6 | Y | 48098 |
| P52193\|CALR_BOVIN | Calreticulin | HF | 30 | 6 | 6 | Y | 48039 |
| P06833\|PYY2_BOVIN | Caltrin | C | 38 | 9 | 7 | Y | 8976 |
| P00517\|KAPCA_BOVIN | cAMP-dependent protein kinase catalytic subunit alpha | C | 27 | 5 | 5 | N | 40620 |
| P00514\|KAP0_BOVIN | cAMP-dependent protein kinase type I-alpha regulatory subunit | C | 18 | 5 | 4 | Y | 42893 |
| P00515\|KAP2_BOVIN | cAMP-dependent protein kinase type II-alpha regulatory subunit | HF | 18 | 7 | 6 | N | 45094 |
| P00921\|CAH2_BOVIN | Carbonic anhydrase 2 | HF | 18 | 2 | 2 | N | 29114 |

| | | | | | | | |
|---|---|---|---|---|---|---|---|
| tr\|F1N0H3\|F1N0H3_BOVIN | Carbonic anhydrase 2 (Fragment) | HF | 19 | 2 | 2 | N | 27750 |
| tr\|E1BN79\|E1BN79_BOVIN | Carboxylic ester hydrolase | C | 53 | 29 | 26 | Y | 64339 |
| tr\|F1MIM4\|F1MIM4_BOVIN | Carboxylic ester hydrolase | HF | 2 | 3 | 0 | N | 67021 |
| tr\|A6BML7\|A6BML7_BOVIN | Carboxypeptidase | C | 31 | 14 | 14 | Y | 53981 |
| Q17QK3\|CBPQ_BOVIN | Carboxypeptidase Q | C | 6 | 2 | 2 | N | 51679 |
| tr\|Q6VAN8\|Q6VAN8_BOVIN | Carcinoembryonic antigen-related cell adhesion molecule 1 | C | 6 | 2 | 2 | Y | 47944 |
| tr\|Q6VAN7\|Q6VAN7_BOVIN | Carcinoembryonic antigen-related cell adhesion molecule 1 isoform 3Ls | C | 6 | 2 | 2 | Y | 47167 |
| tr\|Q6VAN6\|Q6VAN6_BOVIN | Carcinoembryonic antigen-related cell adhesion molecule 1 isoform 3S | C | 6 | 2 | 2 | Y | 40857 |
| tr\|Q6VAN5\|Q6VAN5_BOVIN | Carcinoembryonic antigen-related cell adhesion molecule 1 isoform 3Ss | C | 7 | 2 | 2 | Y | 40080 |
| tr\|J9U8U3\|J9U8U3_BOVIN | Cardiomyopathy associated protein 3 (Fragment) | HF | 1 | 2 | 2 | Y | 377297 |
| P07688\|CATB_BOVIN | Cathepsin B | C | 53 | 15 | 15 | Y | 36661 |
| tr\|F1MMR6\|F1MMR6_BOVIN | Cathepsin D | C | 49 | 19 | 19 | Y | 44705 |
| tr\|Q0VCU3\|Q0VCU3_BOVIN | Cathepsin F | LF | 21 | 7 | 7 | Y | 50893 |
| P25975\|CATL1_BOVIN | Cathepsin L1 | C | 61 | 18 | 18 | Y | 37347 |
| tr\|B0JYN1\|B0JYN1_BOVIN | Cathepsin L2 | C | 61 | 18 | 18 | Y | 37347 |
| Q5E998\|CATL2_BOVIN | Cathepsin L2 | C | 61 | 18 | 18 | Y | 37393 |
| P25326\|CATS_BOVIN | Cathepsin S | C | 49 | 10 | 9 | Y | 37176 |
| tr\|A5PJF7\|A5PJF7_BOVIN | C-C motif chemokine | LF | 39 | 11 | 10 | Y | 11100 |
| P28291\|CCL2_BOVIN | C-C motif chemokine 2 | C | 55 | 16 | 16 | Y | 11114 |
| tr\|F1MQT9\|F1MQT9_BOVIN | CD44 antigen (Fragment) | HF | 3 | 2 | 2 | N | 84147 |
| tr\|Q32PA1\|Q32PA1_BOVIN | CD59 molecule complement regulatory protein | C | 9 | 1 | 1 | Y | 13663 |
| P30932\|CD9_BOVIN | CD9 antigen | C | 20 | 3 | 3 | Y | 25258 |
| tr\|A6QLC4\|A6QLC4_BOVIN | CDH1 protein | C | 6 | 6 | 6 | Y | 97975 |
| tr\|A6QP71\|A6QP71_BOVIN | CDH15 protein | HF | 1 | 1 | 0 | N | 77042 |
| tr\|Q0VCE8\|Q0VCE8_BOVIN | CEACAM8 protein | C | 7 | 2 | 2 | Y | 40110 |
| tr\|Q148D9\|Q148D9_BOVIN | Cellular repressor of E1A-stimulated genes 1 | C | 55 | 7 | 7 | Y | 23948 |
| tr\|Q66LI3\|Q66LI3_BOVIN | CENP-C (Fragment) | HF | 3 | 1 | 1 | Y | 37560 |
| tr\|E1BJ15\|E1BJ15_BOVIN | Centrosomal protein of 290 kDa | C | 1 | 2 | 1 | Y | 290363 |
| Q9TU23\|CE290_BOVIN | Centrosomal protein of 290 kDa (Fragment) | C | 1 | 2 | 1 | Y | 171786 |
| Q1ZYR0\|CLN5_BOVIN | Ceroid-lipofuscinosis neuronal protein 5 | C | 19 | 5 | 5 | Y | 41227 |

| | | | | | | | |
|---|---|---|---|---|---|---|---|
| Q9XSA7\|CLIC4_BOVIN | Chloride intracellular channel protein 4 | LF | 7 | 1 | 1 | Y | 28727 |
| tr\|Q32LC5\|Q32LC5_BOVIN | CKLF-like MARVEL transmembrane domain containing 2 | C | 20 | 2 | 2 | N | 25830 |
| P49951\|CLH1_BOVIN | Clathrin heavy chain 1 | HF | 3 | 3 | 3 | N | 191587 |
| tr\|F1MPU0\|F1MPU0_BOVIN | Clathrin heavy chain 1 (Fragment) | HF | 3 | 3 | 3 | N | 189936 |
| P17697\|CLUS_BOVIN | Clusterin | C | 63 | 62 | 59 | Y | 51114 |
| Q28107\|FA5_BOVIN | Coagulation factor V | C | 1 | 3 | 0 | Y | 248981 |
| tr\|F1MTT3\|F1MTT3_BOVIN | Coagulation factor XII | LF | 3 | 1 | 1 | N | 67235 |
| Q3SYW2\|CO2_BOVIN | Complement C2 | C | 11 | 6 | 6 | Y | 82906 |
| tr\|Q0V7N2\|Q0V7N2_BOVIN | Complement C2 | C | 11 | 6 | 6 | Y | 86999 |
| tr\|A0A0A0MP91\|A0A0A0MP91_BOVIN | Complement C2 | C | 11 | 6 | 6 | Y | 82907 |
| Q2UVX4\|CO3_BOVIN | Complement C3 | C | 35 | 45 | 44 | Y | 187252 |
| P81187\|CFAB_BOVIN | Complement factor B | C | 35 | 17 | 17 | Y | 85366 |
| Q28085\|CFAH_BOVIN | Complement factor H | C | 74 | 80 | 33 | Y | 140374 |
| tr\|F1MC45\|F1MC45_BOVIN | Complement factor H (Fragment) | C | 63 | 47 | 4 | Y | 96593 |
| tr\|A0A075TEJ1\|A0A075TEJ1_BOVIN | Complement regulatory protein variant | LF | 13 | 3 | 3 | Y | 46808 |
| tr\|F1MH70\|F1MH70_BOVIN | Condensin complex subunit 2 | HF | 6 | 1 | 1 | Y | 80694 |
| tr\|F1MVI0\|F1MVI0_BOVIN | Contactin-1 | C | 7 | 4 | 4 | Y | 113306 |
| Q28106\|CNTN1_BOVIN | Contactin-1 | HF | 7 | 4 | 4 | Y | 113385 |
| Q5EA61\|KCRB_BOVIN | Creatine kinase B-type | C | 4 | 1 | 1 | N | 42719 |
| tr\|A4IFS7\|A4IFS7_BOVIN | CTSL1 protein | LF | 8 | 3 | 1 | Y | 37150 |
| P55206\|ANFC_BOVIN | C-type natriuretic peptide | C | 56 | 26 | 24 | Y | 13291 |
| A7MBJ5\|CAND1_BOVIN | Cullin-associated NEDD8-dissociated protein 1 | C | 30 | 21 | 19 | Y | 136375 |
| tr\|Q1RMP3\|Q1RMP3_BOVIN | CutA divalent cation tolerance homolog (E. coli) | LF | 48 | 5 | 5 | Y | 16347 |
| tr\|Q5DPW9\|Q5DPW9_BOVIN | Cystatin E/M | C | 69 | 13 | 12 | Y | 16356 |
| P01035\|CYTC_BOVIN | Cystatin-C | C | 50 | 7 | 7 | Y | 16265 |
| tr\|Q32LP8\|Q32LP8_BOVIN | Cysteine-rich secretory protein 2 | HF | 11 | 2 | 2 | Y | 27143 |
| tr\|Q3ZCL0\|Q3ZCL0_BOVIN | Cysteine-rich secretory protein 2 | C | 28 | 4 | 4 | Y | 27453 |
| Q0IIF9\|CP2U1_BOVIN | Cytochrome P450 2U1 | HF | 3 | 2 | 2 | Y | 61997 |
| tr\|F1MFI9\|F1MFI9_BOVIN | Cytochrome P450 2U1 | HF | 3 | 2 | 2 | Y | 62031 |
| Q3ZC84\|CNDP2_BOVIN | Cytosolic non-specific dipeptidase | HF | 4 | 1 | 1 | N | 52655 |
| tr\|A5D986\|A5D986_BOVIN | Cytosolic sialic acid 9-O-acetylesterase homolog | C | 14 | 5 | 5 | N | 59703 |
| tr\|A6QQL7\|A6QQL7_BOVIN | DCN1-like protein | HF | 5 | 1 | 1 | Y | 28293 |
| tr\|F1MDM6\|F1MDM6_BOVIN | DCN1-like protein | HF | 5 | 1 | 1 | Y | 28278 |

| | | | | | | | |
|---|---|---|---|---|---|---|---|
| tr\|A6QPL7\|A6QPL7_BOVIN | DDN protein | HF | 2 | 2 | 1 | N | 66094 |
| Q58CX7\|DIA1R_BOVIN | Deleted in autism-related protein 1 homolog | LF | 2 | 1 | 1 | N | 48095 |
| P56541\|DNS2A_BOVIN | Deoxyribonuclease-2-alpha | LF | 8 | 2 | 2 | Y | 40282 |
| tr\|F1MZ33\|F1MZ33_BOVIN | Deoxyribonuclease-2-alpha | LF | 8 | 2 | 2 | Y | 40266 |
| tr\|F1MZF2\|F1MZF2_BOVIN | Desmocollin-2 | C | 7 | 3 | 3 | N | 100553 |
| P33545\|DSC2_BOVIN | Desmocollin-2 (Fragment) | LF | 7 | 2 | 2 | N | 95875 |
| tr\|A0JN77\|A0JN77_BOVIN | Dihydroxyacetone kinase 2 homolog (S. cerevisiae) | C | 25 | 9 | 9 | N | 59160 |
| tr\|F1N455\|F1N455_BOVIN | Dipeptidyl peptidase 1 | LF | 5 | 1 | 1 | N | 51979 |
| P81425\|DPP4_BOVIN | Dipeptidyl peptidase 4 | C | 36 | 24 | 12 | Y | 88370 |
| Q10741\|ADA10_BOVIN | Disintegrin and metalloproteinase domain-containing protein 10 | C | 6 | 3 | 3 | Y | 84188 |
| O77780\|ADAM2_BOVIN | Disintegrin and metalloproteinase domain-containing protein 2 | C | 17 | 7 | 7 | Y | 83151 |
| A3KMX0\|ER6L2_BOVIN | DNA excision repair protein ERCC-6-like 2 | HF | 3 | 2 | 2 | Y | 176298 |
| Q2NL21\|DJC11_BOVIN | DnaJ homolog subfamily C member 11 | HF | 1 | 1 | 1 | N | 63237 |
| tr\|F1N036\|F1N036_BOVIN | DnaJ homolog subfamily C member 3 | C | 12 | 2 | 2 | N | 57731 |
| tr\|V6F7Z2\|V6F7Z2_BOVIN | Down syndrome cell adhesion molecule like 1 | C | 1 | 2 | 1 | N | 230459 |
| tr\|A6QNX2\|A6QNX2_BOVIN | DPP7 protein | C | 43 | 15 | 12 | Y | 53642 |
| tr\|Q5H9M6\|Q5H9M6_BOVIN | Dynein heavy chain (Fragment) | HF | 0 | 1 | 1 | Y | 501567 |
| P61285\|DYL1_BOVIN | Dynein light chain 1  cytoplasmic | HF | 12 | 1 | 1 | Y | 10366 |
| Q3MHR3\|DYL2_BOVIN | Dynein light chain 2  cytoplasmic | HF | 12 | 1 | 1 | Y | 10350 |
| A6QQP7\|DYSF_BOVIN | Dysferlin | HF | 0 | 2 | 1 | Y | 239311 |
| tr\|F1N7D7\|F1N7D7_BOVIN | Dystroglycan | C | 19 | 11 | 9 | Y | 97309 |
| A5D7F8\|SH3R1_BOVIN | E3 ubiquitin-protein ligase SH3RF1 | LF | 1 | 1 | 1 | Y | 87592 |
| A1A4K5\|ENPP2_BOVIN | Ectonucleotide pyrophosphatase/phosphodiesterase family member 2 | C | 7 | 3 | 3 | Y | 101717 |
| P15396\|ENPP3_BOVIN | Ectonucleotide pyrophosphatase/phosphodiesterase family member 3 | C | 35 | 21 | 20 | Y | 99523 |
| tr\|G8JKZ1\|G8JKZ1_BOVIN | Ectonucleotide pyrophosphatase/phosphodiesterase family member 3 | C | 35 | 21 | 20 | Y | 99495 |

| | | | | | | | |
|---|---|---|---|---|---|---|---|
| Q5E9R3\|EHD1_BOVIN | EH domain-containing protein 1 | C | 34 | 12 | 8 | Y | 60682 |
| tr\|A7YY77\|A7YY77_BOVIN | ENDOD1 protein | HF | 3 | 2 | 1 | N | 54868 |
| tr\|Q3SZQ8\|Q3SZQ8_BOVIN | Endopin 2 | LF | 7 | 3 | 2 | N | 47034 |
| tr\|Q5J801\|Q5J801_BOVIN | Endopin 2B | LF | 7 | 3 | 2 | N | 47002 |
| Q95M18\|ENPL_BOVIN | Endoplasmin | C | 13 | 8 | 8 | Y | 92427 |
| O02775\|EAP_BOVIN | Enteric beta-defensin | C | 28 | 2 | 2 | Y | 7127 |
| Q3ZC64\|EFNA1_BOVIN | Ephrin-A1 | C | 40 | 12 | 12 | Y | 23838 |
| P79345\|NPC2_BOVIN | Epididymal secretory protein E1 | C | 63 | 19 | 19 | Y | 16640 |
| tr\|A6QLT7\|A6QLT7_BOVIN | EXOC6 protein | LF | 1 | 2 | 0 | N | 93322 |
| tr\|A7E323\|A7E323_BOVIN | EXOSC10 protein | HF | 3 | 2 | 2 | Y | 80336 |
| P31976\|EZRI_BOVIN | Ezrin | C | 38 | 22 | 14 | Y | 68760 |
| tr\|F1N0Q7\|F1N0Q7_BOVIN | Farnesyl pyrophosphate synthase | HF | 3 | 1 | 1 | Y | 95682 |
| Q71SP7\|FAS_BOVIN | Fatty acid synthase | HF | 0 | 2 | 0 | N | 274552 |
| tr\|O77779\|O77779_BOVIN | Fertilin alpha (Fragment) | LF | 2 | 1 | 1 | Y | 89905 |
| tr\|Q2KJ89\|Q2KJ89_BOVIN | Fibulin 5 | C | 10 | 6 | 6 | Y | 50178 |
| Q5EA62\|FBLN5_BOVIN | Fibulin-5 | C | 10 | 6 | 6 | Y | 50164 |
| tr\|Q5FX62\|Q5FX62_BOVIN | FLICE-like inhibitory protein | HF | 6 | 2 | 1 | Y | 55554 |
| A6QLR4\|FLOT2_BOVIN | Flotillin-2 | HF | 7 | 1 | 1 | Y | 46995 |
| tr\|G3X6T9\|G3X6T9_BOVIN | Flotillin-2 (Fragment) | HF | 8 | 1 | 1 | Y | 42328 |
| P02702\|FOLR1_BOVIN | Folate receptor alpha | C | 32 | 5 | 4 | Y | 27922 |
| tr\|E1BJL8\|E1BJL8_BOVIN | Folate receptor alpha | C | 23 | 4 | 3 | Y | 29753 |
| tr\|F1MT42\|F1MT42_BOVIN | Follicle-stimulating hormone receptor | HF | 2 | 1 | 1 | Y | 78030 |
| Q3SZB7\|F16P1_BOVIN | Fructose-1 6-bisphosphatase 1 | C | 45 | 12 | 12 | Y | 36728 |
| tr\|A6QLL8\|A6QLL8_BOVIN | Fructose-bisphosphate aldolase | C | 19 | 5 | 4 | N | 39436 |
| tr\|Q3ZBY4\|Q3ZBY4_BOVIN | Fructose-bisphosphate aldolase | HF | 4 | 2 | 1 | N | 39382 |
| A6H768\|GALK1_BOVIN | Galactokinase | C | 17 | 4 | 4 | Y | 42227 |
| A7E3W2\|LG3BP_BOVIN | Galectin-3-binding protein | C | 49 | 22 | 21 | Y | 62127 |
| A6QPN6\|GILT_BOVIN | Gamma-interferon-inducible lysosomal thiol reductase | C | 32 | 5 | 5 | Y | 27490 |
| tr\|F1MAU3\|F1MAU3_BOVIN | Gamma-interferon-inducible lysosomal thiol reductase | HF | 32 | 5 | 5 | Y | 27548 |
| tr\|A6QNJ8\|A6QNJ8_BOVIN | GANAB protein (Fragment) | HF | 9 | 6 | 6 | N | 109085 |
| Q863C3\|GRP_BOVIN | Gastrin-releasing peptide | C | 39 | 4 | 4 | N | 14934 |
| tr\|F1N1I6\|F1N1I6_BOVIN | Gelsolin | C | 65 | 35 | 32 | Y | 85687 |

| | | | | | | | |
|---|---|---|---|---|---|---|---|
| tr\|F1MJH1\|F1MJH1_BOVIN | Gelsolin | HF | 70 | 35 | 32 | Y | 80703 |
| Q32LB5\|GPRL1_BOVIN | GLIPR1-like protein 1 | C | 37 | 7 | 7 | Y | 27167 |
| Q3ZBD7\|G6PI_BOVIN | Glucose-6-phosphate isomerase | C | 65 | 27 | 27 | Y | 62855 |
| Q2KHZ8\|GLCM_BOVIN | Glucosylceramidase | HF | 6 | 1 | 1 | Y | 59855 |
| tr\|F1N1D5\|F1N1D5_BOVIN | Glucosylceramidase | HF | 6 | 1 | 1 | Y | 59841 |
| tr\|Q0P598\|Q0P598_BOVIN | Glutaminyl-peptide cyclotransferase | LF | 40 | 7 | 7 | Y | 41205 |
| Q28120\|QPCT_BOVIN | Glutaminyl-peptide cyclotransferase | C | 34 | 6 | 6 | Y | 41224 |
| tr\|F1MEM5\|F1MEM5_BOVIN | Glutamyl aminopeptidase | C | 41 | 33 | 1 | Y | 109820 |
| Q32LQ0\|AMPE_BOVIN | Glutamyl aminopeptidase | HF | 41 | 33 | 1 | Y | 109801 |
| tr\|G3N2N9\|G3N2N9_BOVIN | Glutathione peroxidase | C | 73 | 16 | 10 | Y | 25083 |
| tr\|G3X8D7\|G3X8D7_BOVIN | Glutathione peroxidase | C | 40 | 14 | 8 | Y | 25341 |
| tr\|F1MCF5\|F1MCF5_BOVIN | Glutathione peroxidase (Fragment) | C | 65 | 16 | 9 | Y | 15659 |
| P37141\|GPX3_BOVIN | Glutathione peroxidase 3 | C | 40 | 14 | 8 | Y | 25663 |
| Q9N0V4\|GSTM1_BOVIN | Glutathione S-transferase Mu 1 | HF | 18 | 3 | 2 | Y | 25635 |
| tr\|Q2KIV8\|Q2KIV8_BOVIN | Glutathione S-transferase mu 3 (Brain) | C | 35 | 6 | 5 | Y | 26850 |
| Q2KJE5\|G3PT_BOVIN | Glyceraldehyde-3-phosphate dehydrogenase testis-specific | C | 10 | 2 | 2 | N | 43288 |
| tr\|A5PKD6\|A5PKD6_BOVIN | GNB4 protein | C | 3 | 1 | 1 | N | 37537 |
| tr\|Q0VD58\|Q0VD58_BOVIN | Golgi-localized protein | HF | 2 | 2 | 0 | N | 57258 |
| tr\|A6QLI3\|A6QLI3_BOVIN | GOLSYN protein | HF | 2 | 2 | 0 | N | 73752 |
| tr\|A4IF70\|A4IF70_BOVIN | GPR56 protein | HF | 5 | 2 | 2 | N | 76954 |
| tr\|Q2HJI6\|Q2HJI6_BOVIN | Granulin | C | 17 | 3 | 3 | Y | 63054 |
| tr\|A7E3V7\|A7E3V7_BOVIN | Guanine nucleotide-binding protein  beta-1 subunit (Fragment) | C | 3 | 1 | 1 | N | 37030 |
| P62871\|GBB1_BOVIN | Guanine nucleotide-binding protein G(I)/G(S)/G(T) subunit beta-1 | C | 3 | 1 | 1 | N | 37377 |
| P11017\|GBB2_BOVIN | Guanine nucleotide-binding protein G(I)/G(S)/G(T) subunit beta-2 | C | 3 | 1 | 1 | N | 37331 |
| P04896\|GNAS2_BOVIN | Guanine nucleotide-binding protein G(s) subunit alpha isoforms short | HF | 15 | 3 | 3 | Y | 45709 |
| P38409\|GNA11_BOVIN | Guanine nucleotide-binding protein subunit alpha-11 | LF | 4 | 1 | 1 | N | 42070 |
| P38408\|GNA14_BOVIN | Guanine nucleotide-binding protein subunit alpha-14 | LF | 4 | 1 | 1 | N | 41499 |

| | | | | | | | |
|---|---|---|---|---|---|---|---|
| tr\|M5FI09\|M5FI09_BOVIN | HCG1658583-like | HF | 1 | 2 | 1 | N | 113815 |
| Q0P565\|HDDC2_BOVIN | HD domain-containing protein 2 | HF | 8 | 1 | 1 | N | 23323 |
| tr\|Q2YDE6\|Q2YDE6_BOVIN | HDLBP protein (Fragment) | HF | 6 | 2 | 1 | Y | 32663 |
| Q2TBX4\|HSP13_BOVIN | Heat shock 70 kDa protein 13 | C | 33 | 12 | 12 | Y | 51921 |
| P0CB32\|HS71L_BOVIN | Heat shock 70 kDa protein 1-like | C | 33 | 17 | 8 | Y | 70389 |
| tr\|A7E3S8\|A7E3S8_BOVIN | Heat shock 70kD protein binding protein | C | 12 | 4 | 4 | N | 41445 |
| P19120\|HSP7C_BOVIN | Heat shock cognate 71 kDa protein | C | 9 | 6 | 0 | N | 71241 |
| tr\|K8FK38\|K8FK38_BOVIN | Heat Shock Protein 70 (Fragment) | HF | 15 | 9 | 2 | N | 69421 |
| Q76LV2\|HS90A_BOVIN | Heat shock protein HSP 90-alpha | C | 42 | 27 | 24 | Y | 84731 |
| tr\|G3N2V5\|G3N2V5_BOVIN | Heat shock protein HSP 90-beta | C | 4 | 4 | 1 | Y | 82206 |
| P34933\|HSP72_BOVIN | Heat shock-related 70 kDa protein 2 | HF | 9 | 6 | 0 | N | 69740 |
| Q3SZV7\|HEMO_BOVIN | Hemopexin | C | 27 | 6 | 6 | Y | 52209 |
| Q9MYY0\|HPSE_BOVIN | Heparanase | HF | 4 | 1 | 1 | N | 61077 |
| tr\|F1N1G1\|F1N1G1_BOVIN | Heparanase | HF | 4 | 1 | 1 | N | 61133 |
| Q5E9J1\|HNRPF_BOVIN | Heterogeneous nuclear ribonucleoprotein F | LF | 2 | 1 | 1 | N | 45689 |
| tr\|A2VDN7\|A2VDN7_BOVIN | Heterogeneous nuclear ribonucleoprotein U (Scaffold attachment factor A) | HF | 1 | 1 | 1 | Y | 90453 |
| tr\|Q5W5U3\|Q5W5U3_BOVIN | Hexokinase | C | 10 | 10 | 8 | N | 102207 |
| tr\|F1MZV1\|F1MZV1_BOVIN | Hexokinase | C | 10 | 10 | 8 | N | 102294 |
| tr\|F1MIM3\|F1MIM3_BOVIN | Hexokinase | HF | 2 | 3 | 1 | Y | 102579 |
| tr\|E1BLX2\|E1BLX2_BOVIN | Histone-lysine N-methyltransferase | LF | 0 | 1 | 1 | N | 186042 |
| tr\|F1MNT3\|F1MNT3_BOVIN | Hormone-sensitive lipase | C | 22 | 9 | 7 | Y | 82639 |
| tr\|Q2YDK3\|Q2YDK3_BOVIN | Hyaluronidase | C | 9 | 3 | 3 | Y | 62299 |
| tr\|F1MTV1\|F1MTV1_BOVIN | Hyaluronidase | C | 9 | 3 | 3 | Y | 62281 |
| tr\|Q05B55\|Q05B55_BOVIN | IGK protein | C | 20 | 3 | 3 | N | 26591 |
| tr\|B0JYP6\|B0JYP6_BOVIN | IGK protein | C | 20 | 3 | 3 | N | 26320 |
| tr\|Q3T101\|Q3T101_BOVIN | IGL@ protein | LF | 41 | 9 | 1 | Y | 24640 |
| tr\|Q1RMN8\|Q1RMN8_BOVIN | Immunoglobulin light chain lambda gene cluster | LF | 43 | 9 | 1 | Y | 24536 |
| Q70IB2\|RNS10_BOVIN | Inactive ribonuclease-like protein 10 | C | 38 | 8 | 8 | Y | 23363 |
| tr\|F1MUJ4\|F1MUJ4_BOVIN | Inactive serine protease PAMR1 | LF | 2 | 2 | 1 | N | 80027 |
| Q2NL29\|INO1_BOVIN | Inositol-3-phosphate synthase 1 | C | 12 | 4 | 4 | Y | 60761 |
| tr\|B8QGI3\|B8QGI3_BOVIN | Insulin-like growth factor 2 | C | 5 | 1 | 1 | Y | 19682 |
| tr\|I3PGL3\|I3PGL3_BOVIN | Insulin-like growth factor 2 (Fragment) | HF | 6 | 1 | 1 | Y | 15557 |

| | | | | | | | |
|---|---|---|---|---|---|---|---|
| tr\|Q2F6J3\|Q2F6J3_BOVIN | Insulin-like growth factor 2 preproprotein (Fragment) | HF | 9 | 1 | 1 | Y | 10782 |
| tr\|I6XXP7\|I6XXP7_BOVIN | Insulin-like growth factor binding protein 3 | LF | 25 | 6 | 6 | Y | 31570 |
| P07456\|IGF2_BOVIN | Insulin-like growth factor II | C | 5 | 1 | 1 | Y | 19682 |
| P20959\|IBP3_BOVIN | Insulin-like growth factor-binding protein 3 | LF | 25 | 6 | 6 | Y | 31570 |
| Q05717\|IBP5_BOVIN | Insulin-like growth factor-binding protein 5 | C | 13 | 3 | 3 | Y | 30314 |
| tr\|F1N026\|F1N026_BOVIN | Integral membrane protein 2B | C | 23 | 4 | 4 | N | 30386 |
| Q3T0P7\|ITM2B_BOVIN | Integral membrane protein 2B | LF | 19 | 3 | 3 | Y | 30400 |
| tr\|Q2TBX9\|Q2TBX9_BOVIN | Integrin alpha FG-GAP repeat containing 1 | C | 2 | 1 | 1 | N | 67805 |
| tr\|D3WYX5\|D3WYX5_BOVIN | Interleukin-1 receptor-associated kinase I transcript variant 2 | HF | 2 | 2 | 1 | N | 70585 |
| tr\|A2VDQ0\|A2VDQ0_BOVIN | Intraflagellar transport 81 homolog (Chlamydomonas) | HF | 1 | 1 | 1 | Y | 79485 |
| tr\|Q0QEQ4\|Q0QEQ4_BOVIN | Isocitrate dehydrogenase 1 (Fragment) | HF | 11 | 3 | 3 | N | 41342 |
| Q58CS8-2\|GNPTG_BOVIN | Isoform 2 of N-acetylglucosamine-1-phosphotransferase subunit gamma | C | 11 | 2 | 2 | N | 31407 |
| P33545-2\|DSC2_BOVIN | Isoform 2B of Desmocollin-2 | LF | 7 | 2 | 2 | N | 89768 |
| Q9BGI1-2\|PRDX5_BOVIN | Isoform Cytoplasmic+peroxisomal of Peroxiredoxin-5 mitochondrial | C | 64 | 10 | 10 | Y | 17362 |
| P23795-2\|ACES_BOVIN | Isoform H of Acetylcholinesterase | HF | 2 | 3 | 0 | N | 66908 |
| Q9XT56\|JAM1_BOVIN | Junctional adhesion molecule A | HF | 5 | 1 | 1 | N | 32456 |
| tr\|A6H7J0\|A6H7J0_BOVIN | KCNA2 protein | HF | 1 | 1 | 1 | Y | 56747 |
| tr\|A0JNI2\|A0JNI2_BOVIN | Kelch-like 10 (Drosophila) | HF | 4 | 2 | 2 | N | 68817 |
| Q5XQN5\|K2C5_BOVIN | Keratin type II cytoskeletal 5 | HF | 2 | 1 | 1 | N | 62937 |
| tr\|M0QVZ6\|M0QVZ6_BOVIN | Keratin type II cytoskeletal 5 | HF | 2 | 1 | 1 | N | 60667 |
| tr\|A7YVI7\|A7YVI7_BOVIN | KIAA0415 protein (Fragment) | LF | 2 | 2 | 1 | N | 81606 |
| tr\|E1BEN3\|E1BEN3_BOVIN | Kinesin-like protein | C | 1 | 1 | 1 | N | 115147 |
| tr\|G3X690\|G3X690_BOVIN | Kinesin-like protein | HF | 1 | 2 | 1 | Y | 157581 |
| tr\|E1BG49\|E1BG49_BOVIN | Kinesin-like protein | HF | 0 | 1 | 0 | N | 317118 |
| tr\|E1B715\|E1B715_BOVIN | Kinesin-like protein | HF | 2 | 2 | 1 | N | 89819 |
| tr\|Q0V8E3\|Q0V8E3_BOVIN | Kinesin-like protein (Fragment) | C | 1 | 1 | 1 | N | 56925 |
| tr\|G3MXV5\|G3MXV5_BOVIN | Kinesin-like protein (Fragment) | LF | 0 | 1 | 1 | Y | 190628 |
| tr\|F1MJK2\|F1MJK2_BOVIN | Kinesin-like protein (Fragment) | LF | 0 | 1 | 1 | Y | 197032 |
| tr\|A5D7M6\|A5D7M6_BOVIN | KRT5 protein | HF | 2 | 1 | 1 | N | 62683 |

| | | | | | | | |
|---|---|---|---|---|---|---|---|
| tr\|A4FV94\|A4FV94_BOVIN | KRT6A protein | HF | 2 | 1 | 1 | N | 60820 |
| tr\|F1MXX6\|F1MXX6_BOVIN | Lactadherin | C | 23 | 9 | 9 | Y | 47846 |
| Q95114\|MFGM_BOVIN | Lactadherin | C | 24 | 9 | 9 | Y | 47411 |
| tr\|B9VPZ5\|B9VPZ5_BOVIN | Lactoferrin | C | 62 | 49 | 3 | Y | 78056 |
| tr\|B3VTM3\|B3VTM3_BOVIN | Lactoferrin | C | 62 | 47 | 1 | Y | 78056 |
| tr\|C7FE01\|C7FE01_BOVIN | Lactoferrin (Fragment) | C | 63 | 49 | 3 | Y | 76275 |
| P24627\|TRFL_BOVIN | Lactotransferrin | C | 62 | 49 | 3 | Y | 78056 |
| Q95M12\|LGMN_BOVIN | Legumain | C | 22 | 8 | 8 | Y | 49284 |
| tr\|A1L542\|A1L542_BOVIN | Leucine rich repeat containing 8 family member E (Fragment) | HF | 2 | 2 | 0 | Y | 69094 |
| tr\|Q29396\|Q29396_BOVIN | Leucine-rich glycoprotein homolog protein (Fragment) | LF | 10 | 1 | 1 | N | 25653 |
| tr\|E1BNS9\|E1BNS9_BOVIN | L-lactate dehydrogenase | C | 30 | 9 | 7 | Y | 36015 |
| P19858\|LDHA_BOVIN | L-lactate dehydrogenase A chain | C | 11 | 3 | 1 | Y | 36598 |
| tr\|A7YY63\|A7YY63_BOVIN | LOC100124517 protein | HF | 1 | 1 | 0 | N | 74694 |
| tr\|A8E4M5\|A8E4M5_BOVIN | LOC100126170 protein | HF | 0 | 1 | 1 | N | 151226 |
| tr\|A6QQ58\|A6QQ58_BOVIN | LOC504248 protein | HF | 9 | 2 | 2 | Y | 35727 |
| tr\|A8E4Q3\|A8E4Q3_BOVIN | LOC511106 protein | HF | 10 | 3 | 1 | Y | 44057 |
| tr\|A6QPD4\|A6QPD4_BOVIN | LOC790886 protein | HF | 11 | 5 | 1 | Y | 45428 |
| Q3SX23\|LONP2_BOVIN | Lon protease homolog 2 peroxisomal | LF | 1 | 1 | 1 | N | 94310 |
| Q32LD3\|LYPD4_BOVIN | Ly6/PLAUR domain-containing protein 4 | C | 15 | 3 | 3 | Y | 26872 |
| Q9MYM4\|LYAG_BOVIN | Lysosomal alpha-glucosidase | C | 16 | 11 | 11 | Y | 104757 |
| Q29451\|MA2B1_BOVIN | Lysosomal alpha-mannosidase | C | 18 | 11 | 10 | Y | 112919 |
| Q3MI05\|PPGB_BOVIN | Lysosomal protective protein | C | 31 | 14 | 14 | Y | 53980 |
| tr\|Q3SZJ7\|Q3SZJ7_BOVIN | Lysosomal-associated membrane protein 2 | HF | 2 | 1 | 1 | Y | 44543 |
| Q9TTK4\|LYST_BOVIN | Lysosomal-trafficking regulator | C | 0 | 2 | 1 | N | 428603 |
| tr\|F1MLZ4\|F1MLZ4_BOVIN | Lysosomal-trafficking regulator | LF | 0 | 1 | 0 | N | 428547 |
| tr\|A0A077S1J5\|A0A077S1J5_BOVIN | Lysozyme A | LF | 16 | 2 | 2 | N | 16293 |
| Q2T9N7\|LYZL4_BOVIN | Lysozyme-like protein 4 | LF | 16 | 2 | 2 | N | 16293 |
| tr\|G3X7E0\|G3X7E0_BOVIN | Lysozyme-like protein 4 | LF | 16 | 2 | 2 | N | 16293 |
| Q8MJ24\|LOXL4_BOVIN | Lysyl oxidase homolog 4 | LF | 3 | 1 | 1 | Y | 84050 |
| tr\|G1K200\|G1K200_BOVIN | Lysyl oxidase homolog 4 (Fragment) | LF | 4 | 1 | 1 | Y | 60036 |
| P80177\|MIF_BOVIN | Macrophage migration inhibitory factor | C | 17 | 2 | 2 | Y | 12343 |
| tr\|A0A0F7RPX0\|A0A0F7RPX0_BOVIN | Macrophage migration inhibitory factor | C | 17 | 2 | 2 | Y | 12343 |

| | | | | | | | |
|---|---|---|---|---|---|---|---|
| tr\|Q9XS94\|Q9XS94_BOVIN | Major fibrous sheath protein | LF | 3 | 2 | 2 | Y | 93988 |
| tr\|A8WDJ5\|A8WDJ5_BOVIN | Major prion protein | C | 9 | 2 | 2 | Y | 29391 |
| P10279\|PRIO_BOVIN | Major prion protein | C | 9 | 2 | 2 | Y | 28614 |
| tr\|K7ZJV0\|K7ZJV0_BOVIN | Major prion protein | C | 9 | 2 | 2 | Y | 27810 |
| tr\|A7J4K5\|A7J4K5_BOVIN | Major prion protein | C | 9 | 2 | 2 | Y | 27837 |
| tr\|Q2LDZ4\|Q2LDZ4_BOVIN | Major prion protein | C | 9 | 2 | 2 | Y | 28661 |
| tr\|D5G2D5\|D5G2D5_BOVIN | Major prion protein | C | 9 | 2 | 2 | Y | 28636 |
| tr\|B3F6B5\|B3F6B5_BOVIN | Major prion protein | C | 9 | 2 | 2 | Y | 28676 |
| tr\|A6YK35\|A6YK35_BOVIN | Major prion protein | C | 9 | 2 | 2 | Y | 28614 |
| tr\|Q864M0\|Q864M0_BOVIN | Major prion protein | C | 9 | 2 | 2 | Y | 28596 |
| tr\|B3GRV8\|B3GRV8_BOVIN | Major prion protein (Fragment) | C | 11 | 2 | 2 | Y | 23470 |
| tr\|Q6EIP9\|Q6EIP9_BOVIN | Major prion protein (Fragment) | C | 25 | 2 | 2 | Y | 11714 |
| Q3T145\|MDHC_BOVIN | Malate dehydrogenase  cytoplasmic | C | 13 | 3 | 3 | Y | 36438 |
| tr\|B8Y898\|B8Y898_BOVIN | Malic enzyme | C | 11 | 3 | 3 | N | 63894 |
| tr\|F1N3V0\|F1N3V0_BOVIN | Malic enzyme | C | 11 | 3 | 3 | N | 63787 |
| A6QLI0\|EPDR1_BOVIN | Mammalian ependymin-related protein 1 | C | 16 | 4 | 4 | Y | 26485 |
| Q3SZI0\|MPI_BOVIN | Mannose-6-phosphate isomerase | C | 24 | 6 | 6 | Y | 46369 |
| tr\|F1N327\|F1N327_BOVIN | Mannose-6-phosphate isomerase | C | 24 | 6 | 6 | Y | 45958 |
| tr\|A5D7D5\|A5D7D5_BOVIN | MATN2 protein | HF | 5 | 4 | 4 | Y | 106824 |
| tr\|A6H749\|A6H749_BOVIN | MATN4 protein | HF | 16 | 1 | 1 | Y | 14106 |
| tr\|A6QPN5\|A6QPN5_BOVIN | Matrix metallopeptidase 2 (Gelatinase A  72kDa gelatinase  72kDa type IV collagenase) | C | 10 | 2 | 2 | N | 73834 |
| me\|M02769\|ALBU_BOVIN | mature Serum albumin removed signal and pro-peptides at N-term | C | 77 | 54 | 53 | Y | 66433 |
| tr\|A6QQ22\|A6QQ22_BOVIN | MCFD2 protein (Fragment) | LF | 10 | 1 | 1 | N | 18908 |
| tr\|G3MWX4\|G3MWX4_BOVIN | Membrane cofactor protein | LF | 16 | 3 | 3 | Y | 37149 |
| tr\|F1N4W4\|F1N4W4_BOVIN | Membrane cofactor protein | C | 18 | 4 | 3 | Y | 39421 |
| tr\|A0A060INZ0\|A0A060INZ0_BOVIN | Membrane cofactor protein | LF | 15 | 3 | 3 | Y | 39744 |
| tr\|A0A060IE95\|A0A060IE95_BOVIN | Membrane cofactor protein | LF | 15 | 3 | 3 | Y | 39801 |
| tr\|A6QNU3\|A6QNU3_BOVIN | Membrane cofactor protein | LF | 16 | 3 | 3 | Y | 37263 |
| tr\|F6K7I9\|F6K7I9_BOVIN | Membrane cofactor protein | LF | 15 | 3 | 3 | Y | 40134 |
| tr\|F1N4W5\|F1N4W5_BOVIN | Membrane cofactor protein | LF | 15 | 3 | 3 | Y | 39944 |
| tr\|F1N430\|F1N430_BOVIN | Metalloproteinase inhibitor 2 | C | 84 | 61 | 59 | Y | 24387 |
| tr\|Q3T0K7\|Q3T0K7_BOVIN | MFGE8 protein | HF | 23 | 9 | 9 | Y | 47862 |

| | | | | | | | |
|---|---|---|---|---|---|---|---|
| tr\|Q5E9I4\|Q5E9I4_BOVIN | MGAT1 protein | LF | 4 | 1 | 1 | N | 51702 |
| tr\|A5D7E1\|A5D7E1_BOVIN | MGC139254 protein | HF | 10 | 5 | 1 | Y | 60931 |
| tr\|A4IF76\|A4IF76_BOVIN | MGC140461 protein | C | 12 | 3 | 3 | Y | 24456 |
| tr\|A6QPE2\|A6QPE2_BOVIN | MGC148336 protein | LF | 8 | 1 | 1 | N | 17816 |
| tr\|Q9N0E6\|Q9N0E6_BOVIN | Midkine | C | 43 | 5 | 5 | Y | 15683 |
| tr\|Q3SZ28\|Q3SZ28_BOVIN | Midkine (Neurite growth-promoting factor 2) | C | 43 | 5 | 5 | Y | 15743 |
| tr\|A5D9E8\|A5D9E8_BOVIN | Mimecan | C | 27 | 6 | 6 | N | 34196 |
| tr\|G3N088\|G3N088_BOVIN | Mimecan | C | 23 | 6 | 6 | N | 40598 |
| P19879\|MIME_BOVIN | Mimecan | C | 27 | 6 | 6 | N | 34209 |
| tr\|G1K237\|G1K237_BOVIN | Mitochondrial Rho GTPase | LF | 4 | 1 | 1 | Y | 72032 |
| Q2HJF8\|MIRO1_BOVIN | Mitochondrial Rho GTPase 1 | LF | 4 | 1 | 1 | Y | 72078 |
| tr\|A6H797\|A6H797_BOVIN | MLEC protein | C | 9 | 2 | 2 | N | 31566 |
| tr\|Q9N282\|Q9N282_BOVIN | MMP-9 (Fragment) | HF | 3 | 1 | 1 | Y | 36274 |
| Q2HJ49\|MOES_BOVIN | Moesin | HF | 11 | 8 | 1 | N | 67975 |
| tr\|A6QNL0\|A6QNL0_BOVIN | Monocyte differentiation antigen CD14 | C | 10 | 3 | 3 | Y | 39922 |
| Q95122\|CD14_BOVIN | Monocyte differentiation antigen CD14 | HF | 10 | 3 | 3 | Y | 39667 |
| tr\|A8DBT6\|A8DBT6_BOVIN | Monocyte differentiation antigen CD14 | HF | 10 | 3 | 3 | Y | 39668 |
| A5D7P0\|MPIP3_BOVIN | M-phase inducer phosphatase 3 | LF | 2 | 1 | 1 | N | 53794 |
| tr\|A6QP39\|A6QP39_BOVIN | MSLN protein | C | 62 | 24 | 22 | Y | 44291 |
| tr\|A7E340\|A7E340_BOVIN | Mucin 15 cell surface associated | HF | 3 | 1 | 1 | N | 35685 |
| Q8MI01\|MUC15_BOVIN | Mucin-15 | HF | 3 | 1 | 1 | N | 35715 |
| tr\|E1BGW1\|E1BGW1_BOVIN | Mucin-15 | HF | 3 | 1 | 1 | N | 35683 |
| tr\|Q2TBT6\|Q2TBT6_BOVIN | Multiple inositol polyphosphate histidine phosphatase 1 | LF | 4 | 1 | 1 | Y | 41435 |
| tr\|A6QLD6\|A6QLD6_BOVIN | MYO1B protein | HF | 1 | 1 | 1 | Y | 131830 |
| Q27991\|MYH10_BOVIN | Myosin-10 | HF | 1 | 2 | 1 | N | 229097 |
| Q9BE39\|MYH7_BOVIN | Myosin-7 | HF | 1 | 2 | 1 | N | 223227 |
| tr\|F1N2G0\|F1N2G0_BOVIN | Myosin-7 | HF | 1 | 2 | 1 | N | 223964 |
| Q58CS8\|GNPTG_BOVIN | N-acetylglucosamine-1-phosphotransferase subunit gamma | C | 10 | 2 | 2 | N | 33783 |
| tr\|M5FJT7\|M5FJT7_BOVIN | N-acetylglucosamine-1-phosphotransferase subunit gamma | C | 11 | 2 | 2 | N | 31407 |
| Q1LZH9\|GNS_BOVIN | N-acetylglucosamine-6-sulfatase | C | 23 | 8 | 8 | Y | 62776 |
| tr\|F1MXZ0\|F1MXZ0_BOVIN | N-acetylglucosamine-6-sulfatase | C | 23 | 8 | 8 | Y | 62800 |

| | | | | | | | |
|---|---|---|---|---|---|---|---|
| tr\|Q3T074\|Q3T074_BOVIN | NAD(P)(+)--arginine ADP-ribosyltransferase | C | 5 | 2 | 1 | Y | 43998 |
| tr\|F1MUE3\|F1MUE3_BOVIN | NAD(P)(+)--arginine ADP-ribosyltransferase | C | 5 | 2 | 1 | Y | 43969 |
| tr\|E1BI74\|E1BI74_BOVIN | NAD(P)(+)--arginine ADP-ribosyltransferase (Fragment) | C | 39 | 12 | 11 | Y | 33700 |
| tr\|A6QM01\|A6QM01_BOVIN | NAGLU protein | C | 11 | 3 | 3 | Y | 74076 |
| tr\|A6QNZ3\|A6QNZ3_BOVIN | NCOA7 protein | HF | 3 | 1 | 1 | N | 25239 |
| tr\|A6QPQ1\|A6QPQ1_BOVIN | NEPN protein | HF | 2 | 2 | 0 | N | 58005 |
| P02548\|NFL_BOVIN | Neurofilament light polypeptide | LF | 5 | 3 | 2 | Y | 62646 |
| tr\|Q71UA5\|Q71UA5_BOVIN | Neutrophil beta-defensin 12 (Fragment) | HF | 33 | 3 | 1 | Y | 6464 |
| tr\|A7Z034\|A7Z034_BOVIN | NFKB1 protein | LF | 1 | 1 | 1 | N | 105463 |
| tr\|Q1RMU0\|Q1RMU0_BOVIN | NGFI-A binding protein 2 (EGR1 binding protein 2) | HF | 2 | 2 | 0 | N | 56513 |
| tr\|Q3SZQ1\|Q3SZQ1_BOVIN | Nicastrin | HF | 8 | 2 | 2 | N | 79073 |
| tr\|A2VDU4\|A2VDU4_BOVIN | NR1I2 protein | HF | 1 | 1 | 1 | N | 48525 |
| A5D7E9\|NF2L1_BOVIN | Nuclear factor erythroid 2-related factor 1 | LF | 2 | 3 | 1 | N | 83607 |
| tr\|Q1KNJ0\|Q1KNJ0_BOVIN | Nuclear factor of kappa light polypeptide enhancer in B-cells 1 | HF | 1 | 1 | 1 | N | 105488 |
| Q32KP9\|NTF2_BOVIN | Nuclear transport factor 2 | C | 34 | 2 | 2 | Y | 14478 |
| tr\|Q0IIH5\|Q0IIH5_BOVIN | Nucleobindin 2 | C | 54 | 23 | 22 | Y | 49189 |
| Q0P569\|NUCB1_BOVIN | Nucleobindin-1 | C | 61 | 35 | 34 | Y | 54982 |
| tr\|Q3SZ56\|Q3SZ56_BOVIN | Nucleoporin 88kDa | C | 3 | 3 | 1 | Y | 83772 |
| tr\|A1A4N9\|A1A4N9_BOVIN | Nucleoside diphosphate kinase | HF | 62 | 6 | 4 | Y | 17325 |
| P52175\|NDKA2_BOVIN | Nucleoside diphosphate kinase A 2 | HF | 62 | 6 | 4 | Y | 17298 |
| Q3T0Q4\|NDKB_BOVIN | Nucleoside diphosphate kinase B | HF | 61 | 6 | 4 | Y | 17316 |
| Q32KV6\|SIL1_BOVIN | Nucleotide exchange factor SIL1 | C | 11 | 5 | 5 | Y | 52581 |
| tr\|A7MBI8\|A7MBI8_BOVIN | NUDT9 protein | LF | 3 | 1 | 1 | N | 38612 |
| tr\|A6QM02\|A6QM02_BOVIN | NUP188 protein | LF | 0 | 1 | 1 | N | 196262 |
| tr\|Q3T085\|Q3T085_BOVIN | OGN protein | C | 27 | 6 | 6 | N | 34197 |
| tr\|G3X858\|G3X858_BOVIN | Olfactory receptor | HF | 2 | 1 | 1 | N | 36815 |
| tr\|Q17QF1\|Q17QF1_BOVIN | Oncostatin M receptor | C | 13 | 3 | 3 | Y | 37601 |
| P31096\|OSTP_BOVIN | Osteopontin | C | 57 | 36 | 7 | Y | 30904 |
| tr\|Q2QCT5\|Q2QCT5_BOVIN | Osteopontin (Fragment) | C | 56 | 30 | 1 | Y | 29172 |
| P45478\|PPT1_BOVIN | Palmitoyl-protein thioesterase 1 | C | 25 | 4 | 4 | Y | 34142 |
| tr\|F1MSA1\|F1MSA1_BOVIN | Palmitoyl-protein thioesterase 1 | C | 25 | 4 | 4 | Y | 34089 |

| | | | | | | | |
|---|---|---|---|---|---|---|---|
| tr\|M5FKI8\|M5FKI8_BOVIN | Pancreatic adenocarcinoma upregulated factor-like | C | 68 | 9 | 9 | N | 16970 |
| P00974\|BPT1_BOVIN | Pancreatic trypsin inhibitor | C | 13 | 1 | 1 | Y | 10903 |
| Q58CQ9\|VNN1_BOVIN | Pantetheinase | C | 23 | 7 | 7 | Y | 56947 |
| tr\|A5D7Q6\|A5D7Q6_BOVIN | PCDHGA2 protein | C | 6 | 3 | 3 | Y | 90476 |
| tr\|A4IFR2\|A4IFR2_BOVIN | PCSK1N protein | C | 36 | 7 | 6 | N | 27284 |
| tr\|A6QNL5\|A6QNL5_BOVIN | PDIA6 protein (Fragment) | LF | 3 | 1 | 1 | N | 49624 |
| tr\|A1A4Q6\|A1A4Q6_BOVIN | Peptidase inhibitor 3  skin-derived (SKALP) | HF | 19 | 3 | 1 | N | 18376 |
| tr\|Q2NKS8\|Q2NKS8_BOVIN | Peptidyl-prolyl cis-trans isomerase | HF | 44 | 3 | 3 | N | 11945 |
| P80311\|PPIB_BOVIN | Peptidyl-prolyl cis-trans isomerase B | C | 62 | 19 | 18 | Y | 23744 |
| Q9BGI2\|PRDX4_BOVIN | Peroxiredoxin-4 | HF | 16 | 3 | 3 | Y | 30741 |
| Q9BGI1\|PRDX5_BOVIN | Peroxiredoxin-5  mitochondrial | C | 47 | 10 | 10 | Y | 23253 |
| tr\|A6QPB5\|A6QPB5_BOVIN | PGM1 protein | HF | 2 | 1 | 1 | N | 62197 |
| P13696\|PEBP1_BOVIN | Phosphatidylethanolamine-binding protein 1 | C | 40 | 5 | 5 | N | 20986 |
| tr\|Q3T010\|Q3T010_BOVIN | Phosphatidylethanolamine-binding protein 4 | C | 63 | 10 | 10 | Y | 25146 |
| P32871\|PK3CA_BOVIN | Phosphatidylinositol 4 5-bisphosphate 3-kinase catalytic subunit alpha isoform | C | 1 | 2 | 0 | N | 124328 |
| O02811\|PI4KA_BOVIN | Phosphatidylinositol 4-kinase alpha | HF | 2 | 2 | 1 | Y | 229390 |
| tr\|F1MJS4\|F1MJS4_BOVIN | Phosphoglucomutase-1 | HF | 2 | 1 | 1 | N | 62196 |
| Q08DP0\|PGM1_BOVIN | Phosphoglucomutase-1 | HF | 2 | 1 | 1 | N | 61589 |
| tr\|Q32KN6\|Q32KN6_BOVIN | Phosphoglycerate kinase | C | 74 | 32 | 24 | Y | 44758 |
| Q3T0P6\|PGK1_BOVIN | Phosphoglycerate kinase 1 | C | 21 | 7 | 0 | Y | 44538 |
| Q32KV0\|PGAM2_BOVIN | Phosphoglycerate mutase 2 | C | 59 | 15 | 14 | Y | 28685 |
| tr\|F1N2F2\|F1N2F2_BOVIN | Phosphoglycerate mutase 2 | C | 59 | 15 | 14 | Y | 28699 |
| tr\|M5FJY9\|M5FJY9_BOVIN | Phosphoglycolate phosphatase | C | 24 | 5 | 5 | Y | 34320 |
| Q2T9S4\|PGP_BOVIN | Phosphoglycolate phosphatase | C | 24 | 5 | 5 | Y | 34320 |
| tr\|F1MSD7\|F1MSD7_BOVIN | Phosphoinositide phospholipase C | LF | 1 | 1 | 1 | N | 136216 |
| tr\|Q1RML9\|Q1RML9_BOVIN | Phospholipase A2  group VII (Platelet-activating factor acetylhydrolase  plasma) | HF | 75 | 47 | 2 | Y | 50151 |
| tr\|Q9TS13\|Q9TS13_BOVIN | Phospholipase C beta (Fragment) | HF | 5 | 1 | 1 | N | 21128 |
| tr\|F1MZ89\|F1MZ89_BOVIN | Phospholipid-transporting ATPase (Fragment) | C | 1 | 1 | 1 | N | 166459 |
| tr\|E1BN81\|E1BN81_BOVIN | Phospholipid-transporting ATPase (Fragment) | C | 2 | 1 | 1 | N | 116596 |
| Q95121\|PEDF_BOVIN | Pigment epithelium-derived factor | LF | 12 | 2 | 2 | N | 46229 |
| tr\|Q5S1W6\|Q5S1W6_BOVIN | PITSLRE protein kinase beta 1 | HF | 4 | 2 | 1 | N | 49529 |
| tr\|V6F953\|V6F953_BOVIN | Placenta-expressed transcript 1 protein | C | 32 | 8 | 8 | Y | 26609 |

| | | | | | | | |
|---|---|---|---|---|---|---|---|
| A5D7U1\|PLET1_BOVIN | Placenta-expressed transcript 1 protein | C | 32 | 8 | 8 | Y | 26609 |
| Q9N2I2\|IPSP_BOVIN | Plasma serine protease inhibitor | C | 73 | 31 | 29 | Y | 45297 |
| Q28017\|PAFA_BOVIN | Platelet-activating factor acetylhydrolase | C | 74 | 52 | 50 | Y | 50133 |
| Q2KJ15\|PDGFA_BOVIN | Platelet-derived growth factor subunit A | LF | 12 | 2 | 2 | N | 24009 |
| tr\|F1N2P4\|F1N2P4_BOVIN | Platelet-derived growth factor subunit A | LF | 12 | 2 | 2 | N | 24027 |
| tr\|A0JN47\|A0JN47_BOVIN | Plexin domain containing 2 | C | 11 | 4 | 4 | N | 59468 |
| tr\|C0SQ70\|C0SQ70_BOVIN | Plexin domain containing 2 | C | 11 | 4 | 4 | N | 59215 |
| tr\|Q17QC7\|Q17QC7_BOVIN | Poliovirus receptor-related 2 (Herpesvirus entry mediator B) | C | 4 | 1 | 1 | N | 43262 |
| P18493\|PARP1_BOVIN | Poly [ADP-ribose] polymerase 1 | LF | 1 | 1 | 1 | N | 113486 |
| tr\|F1MU08\|F1MU08_BOVIN | Poly [ADP-ribose] polymerase 1 | LF | 1 | 1 | 1 | N | 113368 |
| Q4GZT3\|PKD2_BOVIN | Polycystin-2 | LF | 2 | 2 | 1 | Y | 109789 |
| P81265\|PIGR_BOVIN | Polymeric immunoglobulin receptor | C | 17 | 6 | 6 | Y | 82435 |
| tr\|E1B9U7\|E1B9U7_BOVIN | Polypeptide N-acetylgalactosaminyltransferase | HF | 2 | 2 | 0 | N | 67746 |
| tr\|F1N554\|F1N554_BOVIN | Polypeptide N-acetylgalactosaminyltransferase (Fragment) | C | 19 | 6 | 5 | Y | 63425 |
| P0CG53\|UBB_BOVIN | Polyubiquitin-B | LF | 15 | 5 | 5 | Y | 34308 |
| P0CH28\|UBC_BOVIN | Polyubiquitin-C | LF | 7 | 5 | 5 | Y | 77570 |
| tr\|E1B9K1\|E1B9K1_BOVIN | Polyubiquitin-C | LF | 15 | 5 | 5 | Y | 34366 |
| tr\|Q08635\|Q08635_BOVIN | Potassium channel (BGK5) (Fragment) | HF | 1 | 1 | 1 | Y | 54153 |
| tr\|Q08D98\|Q08D98_BOVIN | PPP2R5D protein (Fragment) | HF | 2 | 2 | 0 | N | 71823 |
| A6QLY7\|PBIP1_BOVIN | Pre-B-cell leukemia transcription factor-interacting protein 1 | C | 12 | 5 | 5 | Y | 80258 |
| O77588\|PLOD1_BOVIN | Procollagen-lysine 2-oxoglutarate 5-dioxygenase 1 | C | 32 | 15 | 15 | Y | 83487 |
| P26779\|SAP_BOVIN | Prosaposin | C | 70 | 33 | 5 | Y | 58051 |
| tr\|A1L555\|A1L555_BOVIN | Prosaposin | C | 58 | 30 | 2 | Y | 58168 |
| tr\|B1H0W7\|B1H0W7_BOVIN | Prostaglandin D2 synthase 21kDa (Brain) | C | 46 | 8 | 7 | Y | 21229 |
| Q32L99\|PTGR2_BOVIN | Prostaglandin reductase 2 | C | 23 | 4 | 4 | Y | 38400 |
| O02853\|PTGDS_BOVIN | Prostaglandin-H2 D-isomerase | C | 46 | 8 | 7 | Y | 21229 |
| A6H730\|PPAP_BOVIN | Prostatic acid phosphatase | HF | 11 | 3 | 3 | Y | 44622 |
| tr\|M5FI38\|M5FI38_BOVIN | Protease serine 22-like | HF | 19 | 3 | 3 | Y | 32255 |
| tr\|Q08DU0\|Q08DU0_BOVIN | Protease serine 8 | C | 22 | 5 | 5 | Y | 36709 |
| tr\|Q1JP95\|Q1JP95_BOVIN | Proteasome 26S ATPase subunit 3 (Fragment) | HF | 4 | 1 | 1 | N | 47995 |
| tr\|E1BD83\|E1BD83_BOVIN | Proteasome subunit alpha type | C | 4 | 1 | 1 | N | 29203 |

| | | | | | | | |
|---|---|---|---|---|---|---|---|
| tr\|G5E5C3\|G5E5C3_BOVIN | Proteasome subunit alpha type | HF | 17 | 3 | 3 | Y | 27284 |
| tr\|A7E3D5\|A7E3D5_BOVIN | Proteasome subunit alpha type (Fragment) | HF | 5 | 1 | 1 | N | 26567 |
| Q3T0X5\|PSA1_BOVIN | Proteasome subunit alpha type-1 | C | 30 | 5 | 5 | Y | 29586 |
| Q3ZCK9\|PSA4_BOVIN | Proteasome subunit alpha type-4 | C | 24 | 4 | 4 | Y | 29484 |
| Q2YDE4\|PSA6_BOVIN | Proteasome subunit alpha type-6 | HF | 17 | 3 | 3 | Y | 27399 |
| Q3ZBG0\|PSA7_BOVIN | Proteasome subunit alpha type-7 | HF | 4 | 1 | 1 | N | 27869 |
| tr\|G5E589\|G5E589_BOVIN | Proteasome subunit beta type | C | 28 | 5 | 5 | Y | 26318 |
| tr\|B0JYN8\|B0JYN8_BOVIN | Proteasome subunit beta type | HF | 18 | 3 | 3 | N | 22993 |
| Q2TBX6\|PSB1_BOVIN | Proteasome subunit beta type-1 | C | 28 | 5 | 5 | Y | 26246 |
| Q5E9K0\|PSB2_BOVIN | Proteasome subunit beta type-2 | C | 21 | 2 | 2 | N | 22896 |
| P33672\|PSB3_BOVIN | Proteasome subunit beta type-3 | HF | 18 | 3 | 3 | N | 22993 |
| Q32KL2\|PSB5_BOVIN | Proteasome subunit beta type-5 | C | 24 | 4 | 4 | Y | 28609 |
| Q3MHN0\|PSB6_BOVIN | Proteasome subunit beta type-6 | HF | 21 | 2 | 2 | Y | 25542 |
| tr\|F1MTI7\|F1MTI7_BOVIN | Protein CutA | C | 68 | 6 | 6 | Y | 16446 |
| tr\|F1N5T0\|F1N5T0_BOVIN | Protein CutA | C | 59 | 6 | 6 | Y | 18932 |
| Q5E946\|PARK7_BOVIN | Protein deglycase DJ-1 | C | 33 | 6 | 6 | Y | 20035 |
| tr\|Q0V8C4\|Q0V8C4_BOVIN | Protein disulfide isomerase-associated 6 (Fragment) | LF | 7 | 1 | 1 | N | 22158 |
| tr\|A6H7J6\|A6H7J6_BOVIN | Protein disulfide-isomerase | C | 8 | 2 | 2 | N | 57204 |
| tr\|A5D7E8\|A5D7E8_BOVIN | Protein disulfide-isomerase | C | 2 | 1 | 1 | N | 56930 |
| P05307\|PDIA1_BOVIN | Protein disulfide-isomerase | LF | 5 | 2 | 2 | N | 57266 |
| P38657\|PDIA3_BOVIN | Protein disulfide-isomerase A3 | C | 2 | 1 | 1 | N | 56930 |
| A5PKI3\|FAM3C_BOVIN | Protein FAM3C | C | 32 | 6 | 6 | Y | 24797 |
| Q3B7M3\|FA65B_BOVIN | Protein FAM65B | HF | 1 | 2 | 0 | N | 111933 |
| tr\|Q1RMR9\|Q1RMR9_BOVIN | Protein kinase C and casein kinase substrate in neurons 2 | LF | 2 | 1 | 1 | N | 55891 |
| tr\|F1MX65\|F1MX65_BOVIN | Protein OS-9 | C | 4 | 2 | 2 | N | 75807 |
| Q3MHX6\|OS9_BOVIN | Protein OS-9 | C | 4 | 2 | 2 | N | 75779 |
| Q3T0W4\|PP1R7_BOVIN | Protein phosphatase 1 regulatory subunit 7 | HF | 5 | 1 | 1 | N | 41388 |
| tr\|Q0VC12\|Q0VC12_BOVIN | Protein YIPF | LF | 3 | 1 | 1 | N | 37987 |
| Q3SYY2\|TPST2_BOVIN | Protein-tyrosine sulfotransferase 2 | C | 12 | 4 | 3 | Y | 41967 |
| tr\|F1MKG9\|F1MKG9_BOVIN | Protein-tyrosine sulfotransferase 2 | LF | 12 | 2 | 2 | N | 41957 |
| tr\|Q3SZ81\|Q3SZ81_BOVIN | PSMC3 protein (Fragment) | HF | 3 | 1 | 1 | N | 48094 |
| tr\|Q687I9\|Q687I9_BOVIN | Purine nucleoside phosphorylase | LF | 3 | 1 | 1 | N | 32067 |

| | | | | | | | |
|---|---|---|---|---|---|---|---|
| P55859\|PNPH_BOVIN | Purine nucleoside phosphorylase | LF | 3 | 1 | 1 | N | 32037 |
| tr\|Q9BEG4\|Q9BEG4_BOVIN | Putative alpha-2 3-sialyltransferase (Fragment) | C | 8 | 2 | 2 | N | 37322 |
| tr\|F1MIH9\|F1MIH9_BOVIN | Putative phospholipase B-like 2 | C | 20 | 7 | 7 | Y | 65723 |
| tr\|A5D7Q2\|A5D7Q2_BOVIN | Putative uncharacterized protein | C | 26 | 7 | 7 | Y | 51671 |
| tr\|Q58D51\|Q58D51_BOVIN | Putative uncharacterized protein DKFZp313G1735 | LF | 10 | 3 | 3 | N | 61353 |
| tr\|Q0V8I4\|Q0V8I4_BOVIN | Putative uncharacterized protein FLJ32810 | HF | 2 | 1 | 1 | N | 48446 |
| tr\|Q2NKT7\|Q2NKT7_BOVIN | Putative uncharacterized protein MGC137030 | C | 8 | 1 | 1 | N | 16149 |
| tr\|A5D984\|A5D984_BOVIN | Pyruvate kinase | HF | 11 | 4 | 4 | Y | 57949 |
| tr\|Q3ZC87\|Q3ZC87_BOVIN | Pyruvate kinase (Fragment) | HF | 10 | 4 | 4 | Y | 61428 |
| P21856\|GDIA_BOVIN | Rab GDP dissociation inhibitor alpha | C | 33 | 9 | 4 | Y | 50566 |
| P50397\|GDIB_BOVIN | Rab GDP dissociation inhibitor beta | C | 20 | 6 | 1 | Y | 50488 |
| tr\|A6QLS9\|A6QLS9_BOVIN | RAB10 protein | HF | 10 | 2 | 1 | N | 22541 |
| tr\|Q3ZBG1\|Q3ZBG1_BOVIN | RAB14 protein | HF | 12 | 2 | 1 | N | 23897 |
| tr\|Q148J4\|Q148J4_BOVIN | RAB2A  member RAS oncogene family | LF | 13 | 2 | 2 | N | 20851 |
| Q32LP2\|RADI_BOVIN | Radixin | C | 12 | 9 | 0 | N | 68568 |
| tr\|F1MJJ8\|F1MJJ8_BOVIN | Radixin (Fragment) | C | 12 | 9 | 0 | N | 68584 |
| tr\|Q0VD52\|Q0VD52_BOVIN | RAS guanyl releasing protein 3 (Calcium and DAG-regulated) | LF | 1 | 1 | 1 | Y | 78224 |
| Q2TA29\|RB11A_BOVIN | Ras-related protein Rab-11A | LF | 9 | 2 | 2 | N | 24470 |
| tr\|F2Z4D5\|F2Z4D5_BOVIN | Ras-related protein Rab-11A | LF | 9 | 2 | 2 | N | 24394 |
| Q3MHP2\|RB11B_BOVIN | Ras-related protein Rab-11B | C | 25 | 6 | 6 | N | 24488 |
| A4FV54\|RAB8A_BOVIN | Ras-related protein Rab-8A | HF | 11 | 2 | 1 | N | 23684 |
| Q2HJI8\|RAB8B_BOVIN | Ras-related protein Rab-8B | HF | 8 | 2 | 1 | Y | 23656 |
| tr\|F1N2J9\|F1N2J9_BOVIN | Ras-related protein Rab-8B | HF | 8 | 2 | 1 | Y | 23686 |
| P62833\|RAP1A_BOVIN | Ras-related protein Rap-1A | HF | 7 | 1 | 1 | N | 20987 |
| P61223\|RAP1B_BOVIN | Ras-related protein Rap-1b | HF | 7 | 1 | 1 | N | 20825 |
| Q2KI30\|REEP2_BOVIN | Receptor expression-enhancing protein 2 | LF | 5 | 2 | 1 | N | 28440 |
| tr\|F1MWF1\|F1MWF1_BOVIN | Receptor protein-tyrosine kinase | HF | 1 | 1 | 1 | Y | 151660 |
| Q3SZ41\|SUH_BOVIN | Recombining binding protein suppressor of hairless | HF | 1 | 1 | 1 | Y | 54341 |
| P81134\|RENR_BOVIN | Renin receptor | C | 46 | 10 | 9 | Y | 39491 |
| tr\|G8JKY4\|G8JKY4_BOVIN | Renin receptor (Fragment) | C | 46 | 10 | 9 | Y | 39481 |
| tr\|Q0VCQ9\|Q0VCQ9_BOVIN | Reticulocalbin 2  EF-hand calcium binding domain | HF | 39 | 10 | 10 | N | 36909 |
| tr\|F1MF50\|F1MF50_BOVIN | Rho-associated protein kinase | LF | 2 | 3 | 1 | Y | 159760 |

| | | | | | | | |
|---|---|---|---|---|---|---|---|
| tr\|Q58DP6\|Q58DP6_BOVIN | Ribonuclease 4 | C | 52 | 10 | 10 | Y | 16938 |
| tr\|W0UV03\|W0UV03_BOVIN | Ribonuclease A C1 | C | 71 | 48 | 36 | Y | 16377 |
| tr\|W0UVI3\|W0UVI3_BOVIN | Ribonuclease A C2 | C | 47 | 20 | 7 | Y | 23701 |
| tr\|W0UV85\|W0UV85_BOVIN | Ribonuclease A K1 | C | 23 | 2 | 2 | Y | 24226 |
| tr\|W0UV04\|W0UV04_BOVIN | Ribonuclease A K2 | C | 42 | 7 | 7 | Y | 24367 |
| tr\|W0UVF3\|W0UVF3_BOVIN | Ribonuclease A M1 | C | 39 | 7 | 7 | Y | 17714 |
| P61823\|RNAS1_BOVIN | Ribonuclease pancreatic | C | 67 | 20 | 7 | Y | 16461 |
| Q3T114\|UK114_BOVIN | Ribonuclease UK114 | HF | 10 | 1 | 1 | N | 14272 |
| tr\|Q3SZN8\|Q3SZN8_BOVIN | Ribonuclease/angiogenin inhibitor 1 | HF | 8 | 2 | 2 | Y | 48850 |
| Q2HJ58\|PRPS1_BOVIN | Ribose-phosphate pyrophosphokinase 1 | HF | 10 | 2 | 2 | Y | 34834 |
| tr\|Q0III8\|Q0III8_BOVIN | RNASET2 protein (Fragment) | C | 25 | 6 | 5 | Y | 32830 |
| P23439\|PDE6B_BOVIN | Rod cGMP-specific 3' 5'-cyclic phosphodiesterase subunit beta | HF | 1 | 2 | 0 | N | 98331 |
| tr\|F1N0U0\|F1N0U0_BOVIN | Rod cGMP-specific 3' 5'-cyclic phosphodiesterase subunit beta (Fragment) | HF | 2 | 2 | 0 | N | 70884 |
| Q0VCY0\|AT2A1_BOVIN | Sarcoplasmic/endoplasmic reticulum calcium ATPase 1 | LF | 1 | 1 | 1 | N | 109290 |
| tr\|A6QPK0\|A6QPK0_BOVIN | SCGB2A2 protein | C | 74 | 10 | 10 | Y | 10427 |
| A0JNP2\|SG1D_BOVIN | Secretoglobin family 1D member | C | 52 | 4 | 4 | Y | 11294 |
| A7MB70\|SEM3C_BOVIN | Semaphorin-3C | C | 14 | 7 | 6 | Y | 85237 |
| P04557\|SFP3_BOVIN | Seminal plasma protein A3 | C | 74 | 51 | 48 | Y | 16140 |
| P81019\|SFP4_BOVIN | Seminal plasma protein BSP-30 kDa | C | 60 | 58 | 56 | Y | 21269 |
| P02784\|SFP1_BOVIN | Seminal plasma protein PDC-109 | C | 79 | 81 | 78 | Y | 15481 |
| P00669\|RNS_BOVIN | Seminal ribonuclease | C | 71 | 48 | 36 | Y | 16377 |
| tr\|Q2NKZ9\|Q2NKZ9_BOVIN | Serine carboxypeptidase 1 | HF | 3 | 1 | 1 | N | 50841 |
| tr\|A1L543\|A1L543_BOVIN | Serine carboxypeptidase 1 protein | HF | 3 | 1 | 1 | N | 45844 |
| tr\|Q2TBS5\|Q2TBS5_BOVIN | Serine peptidase inhibitor  Kazal type 2 (Acrosin-trypsin inhibitor) | C | 31 | 2 | 2 | Y | 9391 |
| tr\|Q32KP8\|Q32KP8_BOVIN | Serine peptidase inhibitor-like  with Kunitz and WAP domains 1 (Eppin) | C | 41 | 5 | 5 | Y | 15093 |
| F1N152\|HTRA1_BOVIN | Serine protease HTRA1 | C | 14 | 4 | 4 | Y | 51907 |
| tr\|Q8HZY1\|Q8HZY1_BOVIN | Serine protease inhibitor clade E member 2 | C | 79 | 37 | 2 | Y | 43877 |
| P01001\|ISK6_BOVIN | Serine protease inhibitor Kazal-type 6 | C | 20 | 1 | 1 | Y | 8663 |
| A0JNI4\|SRR_BOVIN | Serine racemase | HF | 21 | 4 | 4 | Y | 36181 |

| | | | | | | | |
|---|---|---|---|---|---|---|---|
| tr\|G3X6N3\|G3X6N3_BOVIN | Serotransferrin | C | 44 | 30 | 2 | Y | 77666 |
| Q29443\|TRFE_BOVIN | Serotransferrin | C | 45 | 29 | 1 | Y | 77753 |
| tr\|Q2HJF0\|Q2HJF0_BOVIN | Serotransferrin-like | C | 63 | 33 | 4 | Y | 69183 |
| Q9TTE1\|SPA31_BOVIN | Serpin A3-1 | C | 4 | 1 | 1 | N | 46237 |
| A2I7M9\|SPA32_BOVIN | Serpin A3-2 | C | 4 | 1 | 1 | N | 46237 |
| tr\|A0A0A0MP92\|A0A0A0MP92_BOVIN | Serpin A3-7 | LF | 7 | 3 | 2 | N | 47016 |
| tr\|Q08DC0\|Q08DC0_BOVIN | Serpin peptidase inhibitor clade E (Nexin plasminogen activator inhibitor type 1) member 2 | HF | 79 | 37 | 2 | Y | 43891 |
| P02769\|ALBU_BOVIN | Serum albumin | C | 74 | 54 | 53 | Y | 69294 |
| tr\|A4FV01\|A4FV01_BOVIN | SF3B2 protein | HF | 1 | 1 | 1 | N | 100354 |
| tr\|Q2KI90\|Q2KI90_BOVIN | Sialic acid acetylesterase | LF | 21 | 8 | 8 | Y | 59727 |
| A6BMK7\|NEUR1_BOVIN | Sialidase-1 | C | 31 | 6 | 6 | Y | 45433 |
| tr\|A5D960\|A5D960_BOVIN | Sialyltransferase 4A | C | 8 | 2 | 2 | N | 39002 |
| Q3MHE8\|SRPR_BOVIN | Signal recognition particle receptor subunit alpha | C | 11 | 4 | 4 | Y | 69896 |
| tr\|F1MNJ2\|F1MNJ2_BOVIN | Signal recognition particle receptor subunit alpha | C | 11 | 4 | 4 | Y | 65652 |
| tr\|Q862Q3\|Q862Q3_BOVIN | Similar to beta 2-microglobulin (Fragment) | C | 74 | 8 | 8 | Y | 11043 |
| tr\|Q862K7\|Q862K7_BOVIN | Similar to cytoplasmic dynein light chain 1 (Fragment) | HF | 14 | 1 | 1 | Y | 9252 |
| tr\|Q862G1\|Q862G1_BOVIN | Similar to insulin-like growth factor II (Fragment) | HF | 6 | 1 | 1 | Y | 15728 |
| tr\|Q3T0F0\|Q3T0F0_BOVIN | SLC3A2 protein | C | 9 | 4 | 4 | N | 59445 |
| tr\|Q08DL0\|Q08DL0_BOVIN | SLC3A2 protein | C | 9 | 4 | 4 | N | 63183 |
| Q32LM2\|SGTA_BOVIN | Small glutamine-rich tetratricopeptide repeat-containing protein alpha | HF | 4 | 1 | 1 | Y | 34213 |
| tr\|E1B8N5\|E1B8N5_BOVIN | Sodium/potassium-transporting ATPase subunit alpha | C | 10 | 5 | 5 | Y | 113733 |
| P58352\|GTR3_BOVIN | Solute carrier family 2 facilitated glucose transporter member 3 | LF | 7 | 3 | 3 | N | 54020 |
| tr\|A2VDL2\|A2VDL2_BOVIN | Solute carrier family 2 (Facilitated glucose transporter) member 3 | LF | 7 | 3 | 3 | N | 54119 |
| tr\|Q58DQ6\|Q58DQ6_BOVIN | Solute carrier family 3 (Activators of dibasic and neutral amino acid transport) member 2 | C | 10 | 4 | 4 | N | 55595 |
| tr\|Q5EA54\|Q5EA54_BOVIN | Solute carrier family 3 (Activators of dibasic and neutral amino acid transport) member 2 | C | 9 | 4 | 4 | N | 63211 |
| Q58D31\|DHSO_BOVIN | Sorbitol dehydrogenase | C | 25 | 6 | 6 | Y | 38099 |

| | | | | | | | |
|---|---|---|---|---|---|---|---|
| tr\|F1MG03\|F1MG03_BOVIN | Speckle targeted PIP5K1A-regulated poly(A) polymerase | LF | 1 | 1 | 1 | N | 93798 |
| Q32PB3\|SACA4_BOVIN | Sperm acrosome membrane-associated protein 4 | C | 35 | 2 | 2 | Y | 12964 |
| tr\|A4UAF1\|A4UAF1_BOVIN | Sperm associated antigen 11 isoform D | C | 22 | 3 | 2 | Y | 14818 |
| tr\|A4UAF2\|A4UAF2_BOVIN | Sperm associated antigen 11 isoform E | C | 35 | 3 | 2 | Y | 9244 |
| tr\|Q2PMM0\|Q2PMM0_BOVIN | Sperm inner acrosomal membrane protein IAM38 (Fragment) | LF | 21 | 5 | 5 | Y | 34158 |
| tr\|Q4R0H2\|Q4R0H2_BOVIN | Spermadhesin 2 | C | 78 | 44 | 12 | Y | 15221 |
| P82292\|Z13_BOVIN | Spermadhesin Z13 | C | 77 | 33 | 3 | Y | 13383 |
| P29392\|SPAD1_BOVIN | Spermadhesin-1 | C | 85 | 56 | 55 | Y | 15036 |
| Q0VD19\|ASM_BOVIN | Sphingomyelin phosphodiesterase | C | 30 | 12 | 12 | Y | 69392 |
| Q3ZCH0\|GRP75_BOVIN | Stress-70 protein  mitochondrial | HF | 1 | 1 | 1 | N | 73742 |
| tr\|F1MM32\|F1MM32_BOVIN | Sulfhydryl oxidase | HF | 63 | 34 | 33 | Y | 63006 |
| tr\|A6QQA8\|A6QQA8_BOVIN | Sulfhydryl oxidase | C | 63 | 34 | 33 | Y | 62975 |
| tr\|E1BFV8\|E1BFV8_BOVIN | Sulfotransferase | HF | 2 | 2 | 1 | Y | 64965 |
| P00442\|SODC_BOVIN | Superoxide dismutase [Cu-Zn] | C | 47 | 5 | 1 | Y | 15683 |
| tr\|F1MNQ4\|F1MNQ4_BOVIN | Superoxide dismutase [Cu-Zn] | HF | 60 | 5 | 1 | Y | 15687 |
| A0JNN2\|SFTA2_BOVIN | Surfactant-associated protein 2 | C | 13 | 1 | 1 | Y | 8223 |
| tr\|Q2NL10\|Q2NL10_BOVIN | Syntaxin binding protein 2 | HF | 7 | 2 | 2 | Y | 66384 |
| Q3ZBT5\|STX7_BOVIN | Syntaxin-7 | HF | 5 | 1 | 1 | N | 29656 |
| tr\|A5D9B6\|A5D9B6_BOVIN | Syntenin | HF | 8 | 1 | 1 | N | 32415 |
| tr\|A8PVV5\|A8PVV5_BOVIN | SYPL1 protein | C | 18 | 2 | 2 | Y | 19476 |
| O97790\|TBCD1_BOVIN | TBC1 domain family member 1 | HF | 1 | 2 | 1 | N | 132097 |
| tr\|G5E531\|G5E531_BOVIN | T-complex protein 1 subunit alpha | C | 50 | 20 | 19 | Y | 60223 |
| Q3ZBH0\|TCPB_BOVIN | T-complex protein 1 subunit beta | C | 50 | 19 | 19 | Y | 57475 |
| Q2T9X2\|TCPD_BOVIN | T-complex protein 1 subunit delta | C | 27 | 10 | 9 | Y | 58207 |
| tr\|F1N0E5\|F1N0E5_BOVIN | T-complex protein 1 subunit delta | C | 27 | 10 | 9 | Y | 58162 |
| Q2NKZ1\|TCPH_BOVIN | T-complex protein 1 subunit eta | C | 50 | 20 | 19 | Y | 59443 |
| tr\|F1MWR8\|F1MWR8_BOVIN | T-complex protein 1 subunit eta (Fragment) | C | 50 | 20 | 19 | Y | 59180 |
| Q3T0K2\|TCPG_BOVIN | T-complex protein 1 subunit gamma | C | 58 | 23 | 21 | Y | 60586 |
| Q3ZCI9\|TCPQ_BOVIN | T-complex protein 1 subunit theta | C | 44 | 21 | 21 | Y | 59610 |
| Q3MHL7\|TCPZ_BOVIN | T-complex protein 1 subunit zeta | C | 40 | 15 | 8 | Y | 57956 |
| Q3T084\|TCPW_BOVIN | T-complex protein 1 subunit zeta-2 | C | 31 | 11 | 1 | Y | 58032 |
| tr\|F1MP48\|F1MP48_BOVIN | T-complex protein 1 subunit zeta-2 | C | 37 | 12 | 2 | Y | 57965 |

| | | | | | | | |
|---|---|---|---|---|---|---|---|
| tr\|A5D7E6\|A5D7E6_BOVIN | Tetraspanin | C | 4 | 1 | 1 | Y | 30019 |
| tr\|G8JKX6\|G8JKX6_BOVIN | Tetraspanin (Fragment) | C | 23 | 3 | 3 | Y | 22666 |
| Q3T0S3\|TSN1_BOVIN | Tetraspanin-1 | C | 3 | 1 | 1 | N | 26208 |
| tr\|A6QPE3\|A6QPE3_BOVIN | TEX101 protein | C | 41 | 7 | 7 | Y | 27286 |
| tr\|A6QQ20\|A6QQ20_BOVIN | TGOLN2 protein (Fragment) | HF | 8 | 1 | 1 | N | 33444 |
| tr\|Q3SX33\|Q3SX33_BOVIN | Thy-1 cell surface antigen | C | 11 | 2 | 2 | N | 18012 |
| tr\|Q9TS96\|Q9TS96_BOVIN | Thymus HYPOCHOLESTEROLEMIC factor (TPHF) (Superoxide dismutase) (SOD) (Fragments) | HF | 63 | 5 | 1 | Y | 11344 |
| P01267\|THYG_BOVIN | Thyroglobulin | HF | 0 | 1 | 1 | Y | 303221 |
| Q2KIM0\|FUCO_BOVIN | Tissue alpha-L-fucosidase | C | 36 | 9 | 9 | Y | 54089 |
| Q7YRQ8\|TFPI2_BOVIN | Tissue factor pathway inhibitor 2 | C | 69 | 29 | 27 | Y | 26675 |
| tr\|Q866B2\|Q866B2_BOVIN | Toll-like receptor 9 | HF | 2 | 2 | 1 | Y | 115454 |
| tr\|A5H631\|A5H631_BOVIN | Toll-like receptor 9 | HF | 2 | 2 | 1 | Y | 115484 |
| tr\|Q58CU1\|Q58CU1_BOVIN | Transcobalamin II | C | 43 | 10 | 1 | Y | 32235 |
| Q9XSC9\|TCO2_BOVIN | Transcobalamin-2 | C | 54 | 21 | 11 | Y | 47958 |
| tr\|O97567\|O97567_BOVIN | Transcription factor AEBP1 | LF | 5 | 3 | 3 | Y | 82366 |
| P61585\|RHOA_BOVIN | Transforming protein RhoA | C | 52 | 6 | 6 | Y | 21768 |
| tr\|G3X757\|G3X757_BOVIN | Transitional endoplasmic reticulum ATPase | C | 18 | 8 | 8 | Y | 89303 |
| Q3ZBT1\|TERA_BOVIN | Transitional endoplasmic reticulum ATPase | C | 18 | 8 | 8 | Y | 89330 |
| tr\|E1BJ94\|E1BJ94_BOVIN | Transmembrane protease serine | HF | 1 | 1 | 1 | N | 48397 |
| Q58DK4\|TKFC_BOVIN | Triokinase/FMN cyclase | C | 25 | 9 | 9 | N | 59131 |
| Q5E956\|TPIS_BOVIN | Triosephosphate isomerase | LF | 5 | 1 | 1 | N | 26690 |
| Q0V8B6\|TPP1_BOVIN | Tripeptidyl-peptidase 1 | C | 51 | 15 | 15 | Y | 61352 |
| tr\|F1MK08\|F1MK08_BOVIN | Tripeptidyl-peptidase 1 | C | 51 | 15 | 15 | Y | 61339 |
| A5PK39\|TPP2_BOVIN | Tripeptidyl-peptidase 2 | C | 14 | 11 | 11 | Y | 138361 |
| tr\|F1N0M0\|F1N0M0_BOVIN | Tripeptidyl-peptidase 2 | C | 14 | 11 | 11 | Y | 138345 |
| tr\|A3KN16\|A3KN16_BOVIN | TRMU protein | LF | 1 | 1 | 1 | N | 95515 |
| Q32KN8\|TBA3_BOVIN | Tubulin alpha-3 chain | LF | 32 | 8 | 8 | Y | 49926 |
| Q6B856\|TBB2B_BOVIN | Tubulin beta-2B chain | HF | 31 | 9 | 1 | Y | 49953 |
| Q3MHM5\|TBB4B_BOVIN | Tubulin beta-4B chain | C | 37 | 11 | 2 | Y | 49831 |
| Q2HJ81\|TBB6_BOVIN | Tubulin beta-6 chain | HF | 12 | 5 | 1 | Y | 49900 |
| tr\|G3X7R8\|G3X7R8_BOVIN | Tubulin beta-6 chain (Fragment) | HF | 16 | 5 | 1 | Y | 39683 |

| | | | | | | | |
|---|---|---|---|---|---|---|---|
| Q3T077\|TPPP2_BOVIN | Tubulin polymerization-promoting protein family member 2 | C | 34 | 5 | 5 | N | 18564 |
| tr\|Q0VD44\|Q0VD44_BOVIN | Twisted gastrulation homolog 1 (Drosophila) | C | 16 | 2 | 2 | Y | 24937 |
| tr\|E1BND0\|E1BND0_BOVIN | Ubiquitin carboxyl-terminal hydrolase | C | 1 | 3 | 2 | Y | 294173 |
| tr\|J7JXJ4\|J7JXJ4_BOVIN | Ubiquitin carboxyl-terminal hydrolase (Fragment) | C | 42 | 3 | 1 | Y | 16315 |
| tr\|F1N1Z2\|F1N1Z2_BOVIN | Ubiquitin carboxyl-terminal hydrolase (Fragment) | LF | 0 | 1 | 1 | Y | 157582 |
| P23356\|UCHL1_BOVIN | Ubiquitin carboxyl-terminal hydrolase isozyme L1 | HF | 22 | 4 | 2 | Y | 28335 |
| Q2TBG8\|UCHL3_BOVIN | Ubiquitin carboxyl-terminal hydrolase isozyme L3 | HF | 13 | 2 | 2 | N | 26182 |
| P62992\|RS27A_BOVIN | Ubiquitin-40S ribosomal protein S27a | LF | 29 | 5 | 5 | Y | 17965 |
| P63048\|RL40_BOVIN | Ubiquitin-60S ribosomal protein L40 | C | 53 | 7 | 7 | Y | 14728 |
| Q1RMX2\|UB2D2_BOVIN | Ubiquitin-conjugating enzyme E2 D2 | HF | 20 | 2 | 1 | Y | 16735 |
| Q3ZCF7\|UB2D3_BOVIN | Ubiquitin-conjugating enzyme E2 D3 | HF | 20 | 2 | 1 | Y | 16745 |
| tr\|Q32LF7\|Q32LF7_BOVIN | UDP-Gal:betaGlcNAc beta 1 4- galactosyltransferase polypeptide 4 | C | 51 | 18 | 16 | Y | 39461 |
| tr\|F1MSC3\|F1MSC3_BOVIN | Uncharacterized protein | C | 6 | 10 | 8 | Y | 262684 |
| tr\|E1B8A0\|E1B8A0_BOVIN | Uncharacterized protein | C | 16 | 4 | 4 | Y | 37787 |
| tr\|E1B7S8\|E1B7S8_BOVIN | Uncharacterized protein | C | 15 | 4 | 4 | Y | 61273 |
| tr\|E1BBU4\|E1BBU4_BOVIN | Uncharacterized protein | C | 1 | 1 | 1 | N | 93220 |
| tr\|F1MZJ5\|F1MZJ5_BOVIN | Uncharacterized protein | C | 1 | 1 | 1 | N | 88174 |
| tr\|F1MKM4\|F1MKM4_BOVIN | Uncharacterized protein | C | 5 | 2 | 2 | N | 75430 |
| tr\|F1MJV6\|F1MJV6_BOVIN | Uncharacterized protein | C | 6 | 5 | 4 | N | 132531 |
| tr\|F1N1C7\|F1N1C7_BOVIN | Uncharacterized protein | C | 6 | 4 | 4 | Y | 131962 |
| tr\|E1B8K0\|E1B8K0_BOVIN | Uncharacterized protein | C | 0 | 1 | 1 | N | 156487 |
| tr\|F1N2J8\|F1N2J8_BOVIN | Uncharacterized protein | C | 23 | 6 | 6 | Y | 39957 |
| tr\|E1BH06\|E1BH06_BOVIN | Uncharacterized protein | C | 5 | 6 | 6 | N | 192764 |
| tr\|F1N6C0\|F1N6C0_BOVIN | Uncharacterized protein | C | 36 | 2 | 2 | N | 16966 |
| tr\|F1MWD3\|F1MWD3_BOVIN | Uncharacterized protein | C | 54 | 21 | 19 | Y | 59615 |
| tr\|F1MPE1\|F1MPE1_BOVIN | Uncharacterized protein | C | 1 | 2 | 1 | N | 161717 |
| tr\|E1BKX1\|E1BKX1_BOVIN | Uncharacterized protein | C | 4 | 3 | 3 | Y | 152767 |
| tr\|F1MLR4\|F1MLR4_BOVIN | Uncharacterized protein | C | 33 | 7 | 7 | Y | 40688 |
| tr\|E1BK29\|E1BK29_BOVIN | Uncharacterized protein | C | 1 | 1 | 1 | N | 108265 |
| tr\|F1MX50\|F1MX50_BOVIN | Uncharacterized protein | C | 55 | 7 | 7 | Y | 23959 |
| tr\|E1BC47\|E1BC47_BOVIN | Uncharacterized protein | C | 51 | 8 | 8 | Y | 28148 |
| tr\|F6R3I5\|F6R3I5_BOVIN | Uncharacterized protein | C | 29 | 4 | 4 | Y | 27144 |

| | | | | | | | |
|---|---|---|---|---|---|---|---|
| tr\|G3MYQ2\|G3MYQ2_BOVIN | Uncharacterized protein | C | 29 | 7 | 6 | Y | 19185 |
| tr\|F1MLE1\|F1MLE1_BOVIN | Uncharacterized protein | C | 1 | 2 | 1 | N | 171480 |
| tr\|F1MD73\|F1MD73_BOVIN | Uncharacterized protein | C | 2 | 2 | 2 | Y | 190098 |
| tr\|F1N724\|F1N724_BOVIN | Uncharacterized protein | C | 1 | 3 | 2 | Y | 520807 |
| tr\|F1MK55\|F1MK55_BOVIN | Uncharacterized protein | C | 0 | 2 | 1 | N | 508325 |
| tr\|F1N5R7\|F1N5R7_BOVIN | Uncharacterized protein | C | 0 | 2 | 0 | N | 461310 |
| tr\|F1MJJ0\|F1MJJ0_BOVIN | Uncharacterized protein | C | 0 | 1 | 1 | Y | 511306 |
| tr\|F1N4K6\|F1N4K6_BOVIN | Uncharacterized protein | C | 1 | 2 | 1 | N | 205793 |
| tr\|E1BJV0\|E1BJV0_BOVIN | Uncharacterized protein | C | 30 | 14 | 11 | Y | 61025 |
| tr\|E1B9P4\|E1B9P4_BOVIN | Uncharacterized protein | C | 65 | 15 | 12 | Y | 26014 |
| tr\|F1MU34\|F1MU34_BOVIN | Uncharacterized protein | C | 5 | 3 | 3 | N | 107063 |
| tr\|F1N0I3\|F1N0I3_BOVIN | Uncharacterized protein | C | 2 | 3 | 1 | Y | 222214 |
| tr\|G3X6D5\|G3X6D5_BOVIN | Uncharacterized protein | C | 1 | 1 | 1 | N | 120722 |
| tr\|E1BQ21\|E1BQ21_BOVIN | Uncharacterized protein | C | 14 | 3 | 3 | N | 26042 |
| tr\|G3N1D8\|G3N1D8_BOVIN | Uncharacterized protein | C | 30 | 5 | 5 | Y | 25428 |
| tr\|E1BDN9\|E1BDN9_BOVIN | Uncharacterized protein | C | 31 | 5 | 5 | Y | 24903 |
| tr\|G3N2D8\|G3N2D8_BOVIN | Uncharacterized protein | C | 4 | 2 | 2 | N | 60905 |
| tr\|E1BDY3\|E1BDY3_BOVIN | Uncharacterized protein | C | 26 | 24 | 23 | Y | 134635 |
| tr\|E1BI28\|E1BI28_BOVIN | Uncharacterized protein | C | 56 | 6 | 6 | Y | 20903 |
| tr\|E1BB14\|E1BB14_BOVIN | Uncharacterized protein | C | 3 | 1 | 1 | N | 37254 |
| tr\|E1BHY6\|E1BHY6_BOVIN | Uncharacterized protein | C | 17 | 3 | 3 | Y | 63081 |
| tr\|E1BNY8\|E1BNY8_BOVIN | Uncharacterized protein | C | 0 | 1 | 1 | Y | 236171 |
| tr\|F1MT25\|F1MT25_BOVIN | Uncharacterized protein | C | 1 | 2 | 1 | Y | 139586 |
| tr\|E1B782\|E1B782_BOVIN | Uncharacterized protein | C | 0 | 4 | 1 | N | 528175 |
| tr\|F1MWF0\|F1MWF0_BOVIN | Uncharacterized protein | C | 22 | 13 | 13 | Y | 116493 |
| tr\|E1B748\|E1B748_BOVIN | Uncharacterized protein | C | 28 | 17 | 17 | Y | 111684 |
| tr\|F1N2D5\|F1N2D5_BOVIN | Uncharacterized protein | C | 8 | 4 | 4 | Y | 61389 |
| tr\|E1BDR2\|E1BDR2_BOVIN | Uncharacterized protein | C | 8 | 3 | 2 | N | 71516 |
| tr\|F1MLW7\|F1MLW7_BOVIN | Uncharacterized protein | C | 43 | 8 | 8 | Y | 24397 |
| tr\|E1BPQ9\|E1BPQ9_BOVIN | Uncharacterized protein | C | 10 | 2 | 2 | Y | 39739 |
| tr\|F1MS23\|F1MS23_BOVIN | Uncharacterized protein | C | 52 | 6 | 6 | Y | 19711 |
| tr\|E1B9Y3\|E1B9Y3_BOVIN | Uncharacterized protein | C | 20 | 2 | 2 | Y | 21707 |
| tr\|F1MYX5\|F1MYX5_BOVIN | Uncharacterized protein | C | 16 | 5 | 5 | Y | 70112 |
| tr\|E1BLI4\|E1BLI4_BOVIN | Uncharacterized protein | C | 39 | 8 | 8 | Y | 15416 |

| | | | | | | | |
|---|---|---|---|---|---|---|---|
| tr\|M5FMV7\|M5FMV7_BOVIN | Uncharacterized protein | C | 19 | 6 | 6 | Y | 48013 |
| tr\|G3X7F3\|G3X7F3_BOVIN | Uncharacterized protein | C | 27 | 15 | 1 | Y | 74145 |
| tr\|E1BDF3\|E1BDF3_BOVIN | Uncharacterized protein | C | 47 | 20 | 20 | Y | 64357 |
| tr\|E1BG25\|E1BG25_BOVIN | Uncharacterized protein | C | 34 | 15 | 14 | Y | 80694 |
| tr\|E1BM47\|E1BM47_BOVIN | Uncharacterized protein | C | 14 | 15 | 13 | Y | 181029 |
| tr\|G3X8E3\|G3X8E3_BOVIN | Uncharacterized protein | C | 54 | 6 | 6 | Y | 12534 |
| tr\|F1MDF2\|F1MDF2_BOVIN | Uncharacterized protein | C | 55 | 6 | 6 | Y | 12293 |
| tr\|F1MQI3\|F1MQI3_BOVIN | Uncharacterized protein | C | 0 | 3 | 1 | N | 801904 |
| tr\|E1B9F2\|E1B9F2_BOVIN | Uncharacterized protein | C | 1 | 2 | 1 | Y | 210120 |
| tr\|E1BP93\|E1BP93_BOVIN | Uncharacterized protein | C | 1 | 2 | 2 | N | 150932 |
| tr\|E1BBK6\|E1BBK6_BOVIN | Uncharacterized protein | C | 33 | 17 | 17 | Y | 65042 |
| tr\|F1MZU2\|F1MZU2_BOVIN | Uncharacterized protein | C | 14 | 5 | 4 | Y | 82992 |
| tr\|E1B818\|E1B818_BOVIN | Uncharacterized protein | C | 13 | 6 | 6 | N | 73620 |
| tr\|F1MU18\|F1MU18_BOVIN | Uncharacterized protein | C | 13 | 3 | 3 | Y | 37644 |
| tr\|E1BM92\|E1BM92_BOVIN | Uncharacterized protein | C | 20 | 10 | 9 | Y | 91521 |
| tr\|E1BLW6\|E1BLW6_BOVIN | Uncharacterized protein | C | 43 | 8 | 8 | Y | 36163 |
| tr\|F1MUB9\|F1MUB9_BOVIN | Uncharacterized protein | C | 22 | 5 | 5 | Y | 36695 |
| tr\|F1MHN4\|F1MHN4_BOVIN | Uncharacterized protein | C | 1 | 2 | 0 | N | 191981 |
| tr\|F1MGN0\|F1MGN0_BOVIN | Uncharacterized protein | C | 23 | 11 | 10 | Y | 89038 |
| tr\|F1N0F2\|F1N0F2_BOVIN | Uncharacterized protein | C | 14 | 5 | 5 | N | 59673 |
| tr\|F1N2B5\|F1N2B5_BOVIN | Uncharacterized protein | C | 10 | 4 | 4 | N | 55479 |
| tr\|E1BKZ9\|E1BKZ9_BOVIN | Uncharacterized protein | C | 3 | 2 | 2 | N | 91818 |
| tr\|E1BL78\|E1BL78_BOVIN | Uncharacterized protein | C | 9 | 1 | 1 | N | 11458 |
| tr\|F1MQL4\|F1MQL4_BOVIN | Uncharacterized protein | C | 23 | 4 | 4 | Y | 11433 |
| tr\|F1MHF1\|F1MHF1_BOVIN | Uncharacterized protein | C | 38 | 14 | 14 | Y | 46274 |
| tr\|E1BGM7\|E1BGM7_BOVIN | Uncharacterized protein | C | 1 | 2 | 2 | Y | 235348 |
| tr\|F1N757\|F1N757_BOVIN | Uncharacterized protein | C | 0 | 16 | 12 | Y | 3813804 |
| tr\|F2Z4K0\|F2Z4K0_BOVIN | Uncharacterized protein | C | 33 | 9 | 9 | Y | 49960 |
| tr\|E1BHT5\|E1BHT5_BOVIN | Uncharacterized protein | C | 0 | 2 | 0 | N | 572608 |
| tr\|F1MUP9\|F1MUP9_BOVIN | Uncharacterized protein | C | 9 | 3 | 3 | N | 42840 |
| tr\|F1N3U5\|F1N3U5_BOVIN | Uncharacterized protein | C | 50 | 24 | 21 | Y | 52681 |
| tr\|E1BGX2\|E1BGX2_BOVIN | Uncharacterized protein | C | 10 | 1 | 1 | Y | 10743 |
| tr\|Q3T0Z0\|Q3T0Z0_BOVIN | Uncharacterized protein | C | 79 | 26 | 26 | Y | 12729 |
| tr\|E1BNA9\|E1BNA9_BOVIN | Uncharacterized protein | C | 0 | 1 | 1 | N | 331148 |

| | | | | | | | |
|---|---|---|---|---|---|---|---|
| tr\|F1MZ96\|F1MZ96_BOVIN | Uncharacterized protein | C | 20 | 3 | 3 | N | 26562 |
| tr\|F1MH40\|F1MH40_BOVIN | Uncharacterized protein | C | 20 | 3 | 3 | N | 26334 |
| tr\|E1BGB7\|E1BGB7_BOVIN | Uncharacterized protein | C | 24 | 8 | 7 | Y | 71723 |
| tr\|F1MHK9\|F1MHK9_BOVIN | Uncharacterized protein | C | 2 | 3 | 3 | Y | 190767 |
| tr\|E1BB65\|E1BB65_BOVIN | Uncharacterized protein | C | 16 | 2 | 2 | Y | 13305 |
| tr\|E1BJW9\|E1BJW9_BOVIN | Uncharacterized protein | C | 8 | 7 | 7 | N | 131812 |
| tr\|F1MIL6\|F1MIL6_BOVIN | Uncharacterized protein | LF | 14 | 3 | 3 | Y | 28981 |
| tr\|G3MXM6\|G3MXM6_BOVIN | Uncharacterized protein | LF | 5 | 3 | 3 | Y | 82380 |
| tr\|F1MCK2\|F1MCK2_BOVIN | Uncharacterized protein | LF | 0 | 1 | 1 | N | 586979 |
| tr\|F1MYH5\|F1MYH5_BOVIN | Uncharacterized protein | LF | 3 | 2 | 2 | Y | 94002 |
| tr\|E1BNR0\|E1BNR0_BOVIN | Uncharacterized protein | LF | 0 | 2 | 1 | Y | 515764 |
| tr\|F1N7Q8\|F1N7Q8_BOVIN | Uncharacterized protein | LF | 0 | 1 | 1 | N | 226698 |
| tr\|E1BCE1\|E1BCE1_BOVIN | Uncharacterized protein | LF | 0 | 1 | 1 | N | 208169 |
| tr\|F1N353\|F1N353_BOVIN | Uncharacterized protein | LF | 3 | 1 | 1 | Y | 26141 |
| tr\|F1N1F8\|F1N1F8_BOVIN | Uncharacterized protein | LF | 1 | 2 | 1 | Y | 353233 |
| tr\|E1BBS9\|E1BBS9_BOVIN | Uncharacterized protein | LF | 1 | 2 | 1 | Y | 222998 |
| tr\|E1BIN5\|E1BIN5_BOVIN | Uncharacterized protein | LF | 11 | 6 | 6 | N | 88959 |
| tr\|F1MBN4\|F1MBN4_BOVIN | Uncharacterized protein | LF | 2 | 1 | 1 | N | 48095 |
| tr\|E1BMG2\|E1BMG2_BOVIN | Uncharacterized protein | LF | 0 | 1 | 1 | N | 530231 |
| tr\|F1N7A1\|F1N7A1_BOVIN | Uncharacterized protein | LF | 2 | 2 | 1 | N | 71038 |
| tr\|F1N005\|F1N005_BOVIN | Uncharacterized protein | LF | 1 | 2 | 0 | N | 93356 |
| tr\|G3MZZ8\|G3MZZ8_BOVIN | Uncharacterized protein | LF | 0 | 1 | 1 | N | 173493 |
| tr\|E1B725\|E1B725_BOVIN | Uncharacterized protein | LF | 14 | 3 | 3 | N | 49795 |
| tr\|G3N2K4\|G3N2K4_BOVIN | Uncharacterized protein | LF | 26 | 23 | 22 | Y | 121076 |
| tr\|E1BBU2\|E1BBU2_BOVIN | Uncharacterized protein | LF | 1 | 1 | 1 | N | 140261 |
| tr\|E1BF57\|E1BF57_BOVIN | Uncharacterized protein | LF | 1 | 2 | 1 | N | 99115 |
| tr\|F1MYA6\|F1MYA6_BOVIN | Uncharacterized protein | LF | 8 | 1 | 1 | N | 17815 |
| tr\|F1N3S7\|F1N3S7_BOVIN | Uncharacterized protein | LF | 2 | 1 | 1 | N | 98595 |
| tr\|G3MZN3\|G3MZN3_BOVIN | Uncharacterized protein | LF | 4 | 1 | 1 | N | 36209 |
| tr\|E1BKX0\|E1BKX0_BOVIN | Uncharacterized protein | LF | 1 | 1 | 1 | N | 80431 |
| tr\|F1MW79\|F1MW79_BOVIN | Uncharacterized protein | LF | 9 | 1 | 1 | Y | 30881 |
| tr\|G3MY90\|G3MY90_BOVIN | Uncharacterized protein | LF | 34 | 2 | 2 | Y | 6927 |
| tr\|G3MZU3\|G3MZU3_BOVIN | Uncharacterized protein | LF | 4 | 1 | 1 | N | 21190 |
| tr\|E1BLX5\|E1BLX5_BOVIN | Uncharacterized protein | LF | 3 | 1 | 1 | Y | 37899 |

| | | | | | | | |
|---|---|---|---|---|---|---|---|
| tr\|F6PZ29\|F6PZ29_BOVIN | Uncharacterized protein | LF | 12 | 1 | 1 | N | 16328 |
| tr\|E1BKX3\|E1BKX3_BOVIN | Uncharacterized protein | LF | 1 | 2 | 1 | N | 152037 |
| tr\|E1BCU2\|E1BCU2_BOVIN | Uncharacterized protein | LF | 1 | 2 | 0 | N | 162778 |
| tr\|E1BJU4\|E1BJU4_BOVIN | Uncharacterized protein | LF | 1 | 1 | 1 | N | 73764 |
| tr\|E1BIG2\|E1BIG2_BOVIN | Uncharacterized protein | LF | 2 | 2 | 0 | N | 69748 |
| tr\|F1N6B6\|F1N6B6_BOVIN | Uncharacterized protein | LF | 0 | 1 | 1 | N | 196272 |
| tr\|G3MXC4\|G3MXC4_BOVIN | Uncharacterized protein | LF | 3 | 3 | 1 | Y | 86942 |
| tr\|E1BFM2\|E1BFM2_BOVIN | Uncharacterized protein | LF | 3 | 1 | 1 | Y | 47274 |
| tr\|E1BC58\|E1BC58_BOVIN | Uncharacterized protein | LF | 12 | 2 | 2 | N | 24167 |
| tr\|E1BHV1\|E1BHV1_BOVIN | Uncharacterized protein | LF | 0 | 1 | 0 | N | 553749 |
| tr\|E1BE29\|E1BE29_BOVIN | Uncharacterized protein | LF | 0 | 1 | 0 | N | 563805 |
| tr\|E1BAS6\|E1BAS6_BOVIN | Uncharacterized protein | LF | 0 | 1 | 1 | Y | 301194 |
| tr\|F1MLJ7\|F1MLJ7_BOVIN | Uncharacterized protein | LF | 0 | 1 | 1 | N | 169944 |
| tr\|F1MHW2\|F1MHW2_BOVIN | Uncharacterized protein | LF | 1 | 1 | 1 | N | 96954 |
| tr\|E1BPF2\|E1BPF2_BOVIN | Uncharacterized protein | LF | 0 | 1 | 1 | N | 209018 |
| tr\|E1BL02\|E1BL02_BOVIN | Uncharacterized protein | LF | 1 | 3 | 0 | N | 141638 |
| tr\|G3MZQ5\|G3MZQ5_BOVIN | Uncharacterized protein | LF | 1 | 1 | 1 | N | 65851 |
| tr\|E1BIW0\|E1BIW0_BOVIN | Uncharacterized protein | LF | 1 | 2 | 1 | Y | 199465 |
| tr\|G3MX66\|G3MX66_BOVIN | Uncharacterized protein | LF | 28 | 3 | 3 | Y | 21366 |
| tr\|E1BB22\|E1BB22_BOVIN | Uncharacterized protein | LF | 0 | 1 | 1 | Y | 150625 |
| tr\|Q2TBG4\|Q2TBG4_BOVIN | Uncharacterized protein | LF | 5 | 1 | 1 | N | 31207 |
| tr\|E1BJP1\|E1BJP1_BOVIN | Uncharacterized protein | LF | 4 | 1 | 1 | N | 20655 |
| tr\|F1MIB3\|F1MIB3_BOVIN | Uncharacterized protein | LF | 0 | 1 | 1 | N | 283429 |
| tr\|E1BG18\|E1BG18_BOVIN | Uncharacterized protein | LF | 0 | 1 | 1 | N | 166364 |
| tr\|F1MDR1\|F1MDR1_BOVIN | Uncharacterized protein | LF | 0 | 1 | 1 | N | 168829 |
| tr\|F1N369\|F1N369_BOVIN | Uncharacterized protein | LF | 19 | 5 | 5 | Y | 36864 |
| tr\|F1MLW8\|F1MLW8_BOVIN | Uncharacterized protein | LF | 11 | 3 | 1 | Y | 24624 |
| tr\|F1MYN4\|F1MYN4_BOVIN | Uncharacterized protein | HF | 1 | 2 | 1 | Y | 176514 |
| tr\|E1BPL3\|E1BPL3_BOVIN | Uncharacterized protein | HF | 2 | 2 | 0 | N | 83085 |
| tr\|F2Z4F0\|F2Z4F0_BOVIN | Uncharacterized protein | HF | 15 | 4 | 4 | N | 42614 |
| tr\|E1BK03\|E1BK03_BOVIN | Uncharacterized protein | HF | 1 | 2 | 1 | N | 125208 |
| tr\|E1BMU6\|E1BMU6_BOVIN | Uncharacterized protein | HF | 1 | 1 | 1 | N | 89606 |
| tr\|E1BM36\|E1BM36_BOVIN | Uncharacterized protein | HF | 1 | 1 | 1 | N | 88594 |
| tr\|G3MZJ0\|G3MZJ0_BOVIN | Uncharacterized protein | HF | 0 | 2 | 0 | N | 269382 |

| | | | | | | | |
|---|---|---|---|---|---|---|---|
| tr\|E1B719\|E1B719_BOVIN | Uncharacterized protein | HF | 0 | 2 | 0 | N | 277171 |
| tr\|F1MI56\|F1MI56_BOVIN | Uncharacterized protein | HF | 0 | 2 | 0 | N | 275013 |
| tr\|E1B7G6\|E1B7G6_BOVIN | Uncharacterized protein | HF | 1 | 3 | 1 | N | 156126 |
| tr\|E1BNA3\|E1BNA3_BOVIN | Uncharacterized protein | HF | 1 | 2 | 1 | N | 127969 |
| tr\|E1BP14\|E1BP14_BOVIN | Uncharacterized protein | HF | 1 | 2 | 2 | Y | 220695 |
| tr\|F1MKU0\|F1MKU0_BOVIN | Uncharacterized protein | HF | 0 | 1 | 1 | N | 176556 |
| tr\|E1BKQ8\|E1BKQ8_BOVIN | Uncharacterized protein | HF | 0 | 1 | 1 | N | 169690 |
| tr\|E1BJV2\|E1BJV2_BOVIN | Uncharacterized protein | HF | 2 | 2 | 1 | N | 101297 |
| tr\|G3MWL0\|G3MWL0_BOVIN | Uncharacterized protein | HF | 2 | 2 | 1 | N | 104380 |
| tr\|G3N157\|G3N157_BOVIN | Uncharacterized protein | HF | 2 | 3 | 0 | N | 138370 |
| tr\|E1BNE2\|E1BNE2_BOVIN | Uncharacterized protein | HF | 2 | 3 | 0 | N | 134571 |
| tr\|E1BI42\|E1BI42_BOVIN | Uncharacterized protein | HF | 2 | 2 | 2 | Y | 127199 |
| tr\|E1BI97\|E1BI97_BOVIN | Uncharacterized protein | HF | 1 | 3 | 2 | Y | 265693 |
| tr\|E1BFE5\|E1BFE5_BOVIN | Uncharacterized protein | HF | 2 | 2 | 2 | Y | 87741 |
| tr\|F1N5K1\|F1N5K1_BOVIN | Uncharacterized protein | HF | 2 | 2 | 1 | N | 89902 |
| tr\|F1MXP9\|F1MXP9_BOVIN | Uncharacterized protein | HF | 0 | 2 | 0 | N | 369266 |
| tr\|E1BAK5\|E1BAK5_BOVIN | Uncharacterized protein | HF | 1 | 1 | 1 | Y | 105797 |
| tr\|F1ME99\|F1ME99_BOVIN | Uncharacterized protein | HF | 2 | 3 | 1 | N | 133544 |
| tr\|G5E5V6\|G5E5V6_BOVIN | Uncharacterized protein | HF | 1 | 2 | 1 | N | 147931 |
| tr\|E1BJH3\|E1BJH3_BOVIN | Uncharacterized protein | HF | 1 | 2 | 2 | Y | 269607 |
| tr\|F1N2L7\|F1N2L7_BOVIN | Uncharacterized protein | HF | 11 | 2 | 2 | Y | 27117 |
| tr\|F1MRN5\|F1MRN5_BOVIN | Uncharacterized protein | HF | 1 | 2 | 1 | N | 332733 |
| tr\|E1BJE1\|E1BJE1_BOVIN | Uncharacterized protein | HF | 1 | 2 | 1 | Y | 210074 |
| tr\|E1B9R5\|E1B9R5_BOVIN | Uncharacterized protein | HF | 0 | 2 | 1 | Y | 541373 |
| tr\|E1BAK4\|E1BAK4_BOVIN | Uncharacterized protein | HF | 0 | 1 | 0 | N | 255551 |
| tr\|F2Z4F5\|F2Z4F5_BOVIN | Uncharacterized protein | HF | 3 | 1 | 1 | N | 82032 |
| tr\|E1BG89\|E1BG89_BOVIN | Uncharacterized protein | HF | 3 | 1 | 1 | N | 26175 |
| tr\|F1N062\|F1N062_BOVIN | Uncharacterized protein | HF | 1 | 1 | 1 | Y | 121466 |
| tr\|E1BKH6\|E1BKH6_BOVIN | Uncharacterized protein | HF | 1 | 1 | 1 | N | 55007 |
| tr\|F1N6H6\|F1N6H6_BOVIN | Uncharacterized protein | HF | 1 | 2 | 1 | N | 109407 |
| tr\|E1BF15\|E1BF15_BOVIN | Uncharacterized protein | HF | 1 | 1 | 1 | Y | 92868 |
| tr\|E1BK68\|E1BK68_BOVIN | Uncharacterized protein | HF | 1 | 1 | 1 | N | 93698 |
| tr\|F1N6Y1\|F1N6Y1_BOVIN | Uncharacterized protein | HF | 8 | 6 | 6 | N | 109472 |
| tr\|E1B7E3\|E1B7E3_BOVIN | Uncharacterized protein | HF | 1 | 2 | 2 | Y | 259182 |

| | | | | | | | |
|---|---|---|---|---|---|---|---|
| tr\|E1B9D3\|E1B9D3_BOVIN | Uncharacterized protein | HF | 1 | 2 | 1 | Y | 255399 |
| tr\|F1MDU9\|F1MDU9_BOVIN | Uncharacterized protein | HF | 1 | 3 | 2 | N | 242247 |
| tr\|F1MME7\|F1MME7_BOVIN | Uncharacterized protein | HF | 1 | 1 | 1 | N | 94482 |
| tr\|E1BLD1\|E1BLD1_BOVIN | Uncharacterized protein | HF | 0 | 2 | 0 | N | 289337 |
| tr\|F1MNS0\|F1MNS0_BOVIN | Uncharacterized protein | HF | 0 | 2 | 0 | N | 531585 |
| tr\|E1BE11\|E1BE11_BOVIN | Uncharacterized protein | HF | 0 | 3 | 1 | N | 607353 |
| tr\|E1B8E7\|E1B8E7_BOVIN | Uncharacterized protein | HF | 0 | 1 | 1 | Y | 345034 |
| tr\|E1BNY9\|E1BNY9_BOVIN | Uncharacterized protein | HF | 0 | 2 | 0 | N | 482196 |
| tr\|E1BBJ7\|E1BBJ7_BOVIN | Uncharacterized protein | HF | 1 | 2 | 1 | N | 138998 |
| tr\|E1B944\|E1B944_BOVIN | Uncharacterized protein | HF | 2 | 2 | 1 | N | 120638 |
| tr\|F1N685\|F1N685_BOVIN | Uncharacterized protein | HF | 1 | 2 | 1 | N | 283587 |
| tr\|F1MLD6\|F1MLD6_BOVIN | Uncharacterized protein | HF | 1 | 1 | 1 | Y | 56294 |
| tr\|F1MKB0\|F1MKB0_BOVIN | Uncharacterized protein | HF | 1 | 1 | 1 | Y | 56717 |
| tr\|E1B7K3\|E1B7K3_BOVIN | Uncharacterized protein | HF | 1 | 1 | 1 | Y | 63741 |
| tr\|F1MYV2\|F1MYV2_BOVIN | Uncharacterized protein | HF | 0 | 1 | 1 | N | 174976 |
| tr\|F1MBZ1\|F1MBZ1_BOVIN | Uncharacterized protein | HF | 4 | 2 | 2 | N | 68916 |
| tr\|G3N205\|G3N205_BOVIN | Uncharacterized protein | HF | 1 | 2 | 0 | N | 165709 |
| tr\|E1BNZ0\|E1BNZ0_BOVIN | Uncharacterized protein | HF | 1 | 2 | 0 | N | 162896 |
| tr\|M0QVY0\|M0QVY0_BOVIN | Uncharacterized protein | HF | 2 | 1 | 1 | N | 60804 |
| tr\|F1MUY2\|F1MUY2_BOVIN | Uncharacterized protein | HF | 2 | 1 | 1 | N | 60856 |
| tr\|F1MEG3\|F1MEG3_BOVIN | Uncharacterized protein | HF | 0 | 2 | 0 | N | 331081 |
| tr\|G3MXJ5\|G3MXJ5_BOVIN | Uncharacterized protein | HF | 2 | 1 | 1 | Y | 44553 |
| tr\|F1MIA9\|F1MIA9_BOVIN | Uncharacterized protein | HF | 25 | 4 | 4 | Y | 19716 |
| tr\|F1MI18\|F1MI18_BOVIN | Uncharacterized protein | HF | 0 | 1 | 0 | N | 165758 |
| tr\|F1MMS7\|F1MMS7_BOVIN | Uncharacterized protein | HF | 10 | 3 | 1 | Y | 43986 |
| tr\|E1BI82\|E1BI82_BOVIN | Uncharacterized protein | HF | 61 | 32 | 4 | Y | 69149 |
| tr\|E1BN28\|E1BN28_BOVIN | Uncharacterized protein | HF | 1 | 3 | 1 | Y | 150417 |
| tr\|V6F7T8\|V6F7T8_BOVIN | Uncharacterized protein | HF | 2 | 2 | 0 | N | 64531 |
| tr\|G5E5D5\|G5E5D5_BOVIN | Uncharacterized protein | HF | 0 | 2 | 1 | Y | 771059 |
| tr\|F6PRJ0\|F6PRJ0_BOVIN | Uncharacterized protein | HF | 1 | 2 | 0 | Y | 90217 |
| tr\|F1MHR8\|F1MHR8_BOVIN | Uncharacterized protein | HF | 5 | 4 | 4 | Y | 106937 |
| tr\|F1MUK2\|F1MUK2_BOVIN | Uncharacterized protein | HF | 1 | 2 | 0 | N | 110415 |
| tr\|F1N1D8\|F1N1D8_BOVIN | Uncharacterized protein | HF | 3 | 1 | 1 | Y | 55205 |
| tr\|E1BPL8\|E1BPL8_BOVIN | Uncharacterized protein | HF | 4 | 2 | 2 | N | 85899 |

| | | | | | | | |
|---|---|---|---|---|---|---|---|
| tr\|F1MM57\|F1MM57_BOVIN | Uncharacterized protein | HF | 1 | 2 | 2 | Y | 232362 |
| tr\|F1MG13\|F1MG13_BOVIN | Uncharacterized protein | HF | 0 | 1 | 1 | N | 390074 |
| tr\|E1BKQ6\|E1BKQ6_BOVIN | Uncharacterized protein | HF | 0 | 1 | 1 | N | 232838 |
| tr\|E1BKW7\|E1BKW7_BOVIN | Uncharacterized protein | HF | 2 | 2 | 2 | Y | 304077 |
| tr\|E1BF23\|E1BF23_BOVIN | Uncharacterized protein | HF | 1 | 1 | 1 | N | 165262 |
| tr\|F1MGM4\|F1MGM4_BOVIN | Uncharacterized protein | HF | 1 | 2 | 1 | N | 267182 |
| tr\|F1MT60\|F1MT60_BOVIN | Uncharacterized protein | HF | 0 | 3 | 1 | N | 567743 |
| tr\|F1MKW9\|F1MKW9_BOVIN | Uncharacterized protein | HF | 1 | 1 | 1 | N | 105350 |
| tr\|G3MXN4\|G3MXN4_BOVIN | Uncharacterized protein | HF | 1 | 1 | 1 | N | 126454 |
| tr\|E1BFN9\|E1BFN9_BOVIN | Uncharacterized protein | HF | 0 | 1 | 1 | Y | 184471 |
| tr\|F1N184\|F1N184_BOVIN | Uncharacterized protein | HF | 2 | 2 | 0 | N | 59371 |
| tr\|G3N1P3\|G3N1P3_BOVIN | Uncharacterized protein | HF | 3 | 1 | 1 | Y | 62618 |
| tr\|F1N415\|F1N415_BOVIN | Uncharacterized protein | HF | 0 | 3 | 1 | N | 547165 |
| tr\|E1BF59\|E1BF59_BOVIN | Uncharacterized protein | HF | 1 | 3 | 1 | Y | 527646 |
| tr\|F1MBV2\|F1MBV2_BOVIN | Uncharacterized protein | HF | 9 | 2 | 2 | Y | 35658 |
| tr\|E1BAF6\|E1BAF6_BOVIN | Uncharacterized protein | HF | 1 | 2 | 1 | N | 229153 |
| tr\|E1BNJ9\|E1BNJ9_BOVIN | Uncharacterized protein | HF | 18 | 3 | 3 | Y | 34079 |
| tr\|F1MWE0\|F1MWE0_BOVIN | Uncharacterized protein | HF | 3 | 1 | 1 | N | 51055 |
| tr\|E1BH45\|E1BH45_BOVIN | Uncharacterized protein | HF | 1 | 2 | 1 | N | 181758 |
| tr\|E1BHN4\|E1BHN4_BOVIN | Uncharacterized protein | HF | 0 | 4 | 0 | N | 374199 |
| tr\|F1MMR9\|F1MMR9_BOVIN | Uncharacterized protein | HF | 1 | 2 | 0 | N | 208257 |
| tr\|F1MPT4\|F1MPT4_BOVIN | Uncharacterized protein | HF | 0 | 1 | 1 | N | 241466 |
| tr\|E1BBB5\|E1BBB5_BOVIN | Uncharacterized protein | HF | 1 | 2 | 1 | Y | 157116 |
| tr\|F1MWN4\|F1MWN4_BOVIN | Uncharacterized protein | HF | 1 | 2 | 1 | N | 148483 |
| tr\|F1MXV4\|F1MXV4_BOVIN | Uncharacterized protein | HF | 1 | 2 | 1 | N | 200277 |
| tr\|E1BET1\|E1BET1_BOVIN | Uncharacterized protein | HF | 1 | 2 | 1 | Y | 189610 |
| tr\|G3MZA6\|G3MZA6_BOVIN | Uncharacterized protein | HF | 1 | 2 | 0 | N | 103901 |
| tr\|E1BLT3\|E1BLT3_BOVIN | Uncharacterized protein | HF | 1 | 4 | 0 | N | 288890 |
| tr\|F1MHC2\|F1MHC2_BOVIN | Uncharacterized protein | HF | 7 | 2 | 2 | Y | 66356 |
| tr\|G3MZF5\|G3MZF5_BOVIN | Uncharacterized protein | HF | 2 | 2 | 0 | N | 73863 |
| tr\|E1BKM6\|E1BKM6_BOVIN | Uncharacterized protein | HF | 1 | 2 | 1 | N | 118749 |
| tr\|G5E661\|G5E661_BOVIN | Uncharacterized protein | HF | 0 | 2 | 0 | N | 304802 |
| tr\|G5E5W9\|G5E5W9_BOVIN | Uncharacterized protein | HF | 0 | 2 | 0 | N | 286103 |
| tr\|G3N0H1\|G3N0H1_BOVIN | Uncharacterized protein | HF | 1 | 1 | 1 | N | 174158 |

| | | | | | | | |
|---|---|---|---|---|---|---|---|
| tr\|F1MQI1\|F1MQI1_BOVIN | Uncharacterized protein | HF | 1 | 2 | 1 | N | 271479 |
| tr\|E1BP50\|E1BP50_BOVIN | Uncharacterized protein | HF | 1 | 1 | 1 | N | 170961 |
| tr\|E1BLK1\|E1BLK1_BOVIN | Uncharacterized protein | HF | 0 | 2 | 2 | N | 305194 |
| tr\|E1BJC6\|E1BJC6_BOVIN | Uncharacterized protein | HF | 2 | 2 | 2 | N | 61398 |
| tr\|F1ME38\|F1ME38_BOVIN | Uncharacterized protein | HF | 3 | 2 | 2 | N | 118792 |
| tr\|E1BK25\|E1BK25_BOVIN | Uncharacterized protein | HF | 0 | 1 | 1 | N | 164782 |
| tr\|E1BL04\|E1BL04_BOVIN | Uncharacterized protein | HF | 1 | 2 | 2 | Y | 428734 |
| tr\|E1BE98\|E1BE98_BOVIN | Uncharacterized protein | HF | 4 | 2 | 2 | Y | 123142 |
| tr\|F1MRS4\|F1MRS4_BOVIN | Uncharacterized protein | HF | 2 | 2 | 1 | N | 78635 |
| tr\|E1BG37\|E1BG37_BOVIN | Uncharacterized protein | HF | 2 | 2 | 1 | N | 105415 |
| tr\|E1BB98\|E1BB98_BOVIN | Uncharacterized protein | HF | 2 | 2 | 1 | N | 46497 |
| tr\|E1BKY2\|E1BKY2_BOVIN | Uncharacterized protein | HF | 16 | 2 | 2 | Y | 13235 |
| tr\|G5E5W7\|G5E5W7_BOVIN | Uncharacterized protein | HF | 2 | 2 | 2 | N | 96902 |
| tr\|F1ME41\|F1ME41_BOVIN | Uncharacterized protein | HF | 12 | 1 | 1 | Y | 10440 |
| tr\|G3MX54\|G3MX54_BOVIN | Uncharacterized protein | HF | 12 | 1 | 1 | Y | 10378 |
| tr\|F1MU57\|F1MU57_BOVIN | Uncharacterized protein | HF | 19 | 13 | 1 | Y | 88830 |
| tr\|E1B7A3\|E1B7A3_BOVIN | Uncharacterized protein | HF | 12 | 1 | 1 | Y | 10348 |
| tr\|F1MM07\|F1MM07_BOVIN | Uncharacterized protein | HF | 1 | 2 | 1 | N | 222641 |
| tr\|F1MTP1\|F1MTP1_BOVIN | Uncharacterized protein | HF | 0 | 1 | 0 | N | 188038 |
| tr\|F1MS52\|F1MS52_BOVIN | Uncharacterized protein (Fragment) | C | 0 | 1 | 1 | N | 126589 |
| tr\|F1MEW1\|F1MEW1_BOVIN | Uncharacterized protein (Fragment) | C | 2 | 2 | 0 | N | 85072 |
| tr\|G3N3E8\|G3N3E8_BOVIN | Uncharacterized protein (Fragment) | C | 1 | 1 | 1 | Y | 220525 |
| tr\|G3X834\|G3X834_BOVIN | Uncharacterized protein (Fragment) | LF | 1 | 1 | 1 | Y | 104189 |
| tr\|G3X6U1\|G3X6U1_BOVIN | Uncharacterized protein (Fragment) | HF | 3 | 1 | 1 | N | 55098 |
| tr\|F1MQ87\|F1MQ87_BOVIN | Uncharacterized protein (Fragment) | HF | 3 | 1 | 1 | Y | 141516 |
| tr\|F1MYZ3\|F1MYZ3_BOVIN | Uncharacterized protein (Fragment) | HF | 0 | 2 | 2 | Y | 526510 |
| tr\|F1N5D7\|F1N5D7_BOVIN | Uncharacterized protein (Fragment) | HF | 1 | 1 | 1 | N | 129939 |
| tr\|E1BNF6\|E1BNF6_BOVIN | Uncharacterized protein (Fragment) | HF | 0 | 2 | 1 | N | 401667 |
| tr\|E1BK38\|E1BK38_BOVIN | Uncharacterized protein (Fragment) | C | 0 | 2 | 1 | N | 567920 |
| tr\|F1MGY9\|F1MGY9_BOVIN | Uncharacterized protein (Fragment) | C | 37 | 10 | 10 | Y | 37107 |
| tr\|F1MY16\|F1MY16_BOVIN | Uncharacterized protein (Fragment) | C | 1 | 2 | 2 | Y | 256001 |
| tr\|F1MXF5\|F1MXF5_BOVIN | Uncharacterized protein (Fragment) | C | 0 | 2 | 1 | Y | 442146 |
| tr\|F1MNJ4\|F1MNJ4_BOVIN | Uncharacterized protein (Fragment) | C | 18 | 7 | 7 | Y | 55901 |
| tr\|E1BLB4\|E1BLB4_BOVIN | Uncharacterized protein (Fragment) | C | 0 | 2 | 1 | N | 501324 |

| | | | | | | | |
|---|---|---|---|---|---|---|---|
| tr\|F1MGQ1\|F1MGQ1_BOVIN | Uncharacterized protein (Fragment) | C | 64 | 29 | 29 | Y | 35108 |
| tr\|F1N1K6\|F1N1K6_BOVIN | Uncharacterized protein (Fragment) | C | 1 | 4 | 1 | Y | 530684 |
| tr\|F1N5W4\|F1N5W4_BOVIN | Uncharacterized protein (Fragment) | C | 18 | 3 | 3 | Y | 41752 |
| tr\|F1N3E9\|F1N3E9_BOVIN | Uncharacterized protein (Fragment) | C | 22 | 10 | 8 | N | 64127 |
| tr\|F1MPF3\|F1MPF3_BOVIN | Uncharacterized protein (Fragment) | C | 11 | 19 | 19 | Y | 347588 |
| tr\|F1MTZ4\|F1MTZ4_BOVIN | Uncharacterized protein (Fragment) | C | 0 | 1 | 1 | Y | 403272 |
| tr\|G3X6I0\|G3X6I0_BOVIN | Uncharacterized protein (Fragment) | C | 9 | 9 | 9 | Y | 202985 |
| tr\|F1MYQ7\|F1MYQ7_BOVIN | Uncharacterized protein (Fragment) | C | 1 | 3 | 1 | Y | 338694 |
| tr\|F1N5H2\|F1N5H2_BOVIN | Uncharacterized protein (Fragment) | C | 9 | 3 | 3 | N | 53678 |
| tr\|F1MZ40\|F1MZ40_BOVIN | Uncharacterized protein (Fragment) | C | 13 | 5 | 5 | Y | 74692 |
| tr\|F1MUX6\|F1MUX6_BOVIN | Uncharacterized protein (Fragment) | C | 38 | 6 | 5 | Y | 25067 |
| tr\|G3MX12\|G3MX12_BOVIN | Uncharacterized protein (Fragment) | C | 0 | 4 | 1 | N | 518272 |
| tr\|F1ML49\|F1ML49_BOVIN | Uncharacterized protein (Fragment) | C | 4 | 4 | 2 | N | 115899 |
| tr\|G3MWX7\|G3MWX7_BOVIN | Uncharacterized protein (Fragment) | C | 66 | 17 | 16 | Y | 11903 |
| tr\|G3MX67\|G3MX67_BOVIN | Uncharacterized protein (Fragment) | C | 12 | 4 | 4 | N | 36536 |
| tr\|F1N6H1\|F1N6H1_BOVIN | Uncharacterized protein (Fragment) | C | 0 | 2 | 1 | Y | 517637 |
| tr\|F1N6H4\|F1N6H4_BOVIN | Uncharacterized protein (Fragment) | C | 0 | 3 | 1 | Y | 824687 |
| tr\|F1MGK8\|F1MGK8_BOVIN | Uncharacterized protein (Fragment) | C | 4 | 3 | 3 | Y | 110281 |
| tr\|G3MXX5\|G3MXX5_BOVIN | Uncharacterized protein (Fragment) | C | 23 | 9 | 9 | Y | 50285 |
| tr\|F1N4J2\|F1N4J2_BOVIN | Uncharacterized protein (Fragment) | C | 0 | 1 | 0 | N | 518418 |
| tr\|F1MMM6\|F1MMM6_BOVIN | Uncharacterized protein (Fragment) | C | 16 | 12 | 12 | Y | 147331 |
| tr\|F1N4N6\|F1N4N6_BOVIN | Uncharacterized protein (Fragment) | C | 1 | 1 | 1 | N | 120617 |
| tr\|E1BJV1\|E1BJV1_BOVIN | Uncharacterized protein (Fragment) | C | 0 | 1 | 1 | Y | 414894 |
| tr\|F1MF78\|F1MF78_BOVIN | Uncharacterized protein (Fragment) | C | 0 | 3 | 1 | Y | 789349 |
| tr\|G3N0V0\|G3N0V0_BOVIN | Uncharacterized protein (Fragment) | C | 71 | 15 | 15 | Y | 35952 |
| tr\|F6RP72\|F6RP72_BOVIN | Uncharacterized protein (Fragment) | C | 33 | 9 | 9 | Y | 49828 |
| tr\|E1BDR1\|E1BDR1_BOVIN | Uncharacterized protein (Fragment) | C | 27 | 2 | 2 | Y | 21194 |
| tr\|G3N3C5\|G3N3C5_BOVIN | Uncharacterized protein (Fragment) | C | 8 | 5 | 5 | Y | 134188 |
| tr\|F1MY12\|F1MY12_BOVIN | Uncharacterized protein (Fragment) | C | 35 | 5 | 4 | Y | 24484 |
| tr\|F1MC52\|F1MC52_BOVIN | Uncharacterized protein (Fragment) | C | 15 | 2 | 2 | Y | 13457 |
| tr\|F1MPT2\|F1MPT2_BOVIN | Uncharacterized protein (Fragment) | C | 9 | 7 | 0 | Y | 112139 |
| tr\|F1N2X6\|F1N2X6_BOVIN | Uncharacterized protein (Fragment) | C | 10 | 5 | 5 | Y | 104842 |
| tr\|F1MIM0\|F1MIM0_BOVIN | Uncharacterized protein (Fragment) | C | 16 | 2 | 2 | Y | 13059 |
| tr\|F1N6G4\|F1N6G4_BOVIN | Uncharacterized protein (Fragment) | LF | 9 | 2 | 2 | N | 41937 |

| | | | | | | | |
|---|---|---|---|---|---|---|---|
| tr\|G5E5C6\|G5E5C6_BOVIN | Uncharacterized protein (Fragment) | LF | 24 | 5 | 1 | Y | 39701 |
| tr\|F1MY02\|F1MY02_BOVIN | Uncharacterized protein (Fragment) | LF | 2 | 1 | 1 | Y | 89961 |
| tr\|F1MUM9\|F1MUM9_BOVIN | Uncharacterized protein (Fragment) | LF | 1 | 1 | 1 | Y | 130506 |
| tr\|E1BDG9\|E1BDG9_BOVIN | Uncharacterized protein (Fragment) | LF | 1 | 1 | 1 | N | 100240 |
| tr\|F1MX68\|F1MX68_BOVIN | Uncharacterized protein (Fragment) | LF | 5 | 2 | 2 | N | 54462 |
| tr\|F1N6Y7\|F1N6Y7_BOVIN | Uncharacterized protein (Fragment) | LF | 1 | 2 | 1 | N | 138278 |
| tr\|G5E513\|G5E513_BOVIN | Uncharacterized protein (Fragment) | LF | 10 | 2 | 1 | Y | 49970 |
| tr\|G5E5T5\|G5E5T5_BOVIN | Uncharacterized protein (Fragment) | LF | 6 | 2 | 1 | N | 42478 |
| tr\|F1N191\|F1N191_BOVIN | Uncharacterized protein (Fragment) | LF | 14 | 5 | 5 | N | 65367 |
| tr\|F1N157\|F1N157_BOVIN | Uncharacterized protein (Fragment) | LF | 1 | 2 | 1 | Y | 131193 |
| tr\|G5E6C5\|G5E6C5_BOVIN | Uncharacterized protein (Fragment) | LF | 2 | 1 | 1 | Y | 68937 |
| tr\|F1MF64\|F1MF64_BOVIN | Uncharacterized protein (Fragment) | LF | 1 | 2 | 1 | Y | 314665 |
| tr\|F1MDK3\|F1MDK3_BOVIN | Uncharacterized protein (Fragment) | LF | 1 | 2 | 1 | N | 135323 |
| tr\|G5E562\|G5E562_BOVIN | Uncharacterized protein (Fragment) | LF | 2 | 1 | 1 | N | 37891 |
| tr\|F1ML06\|F1ML06_BOVIN | Uncharacterized protein (Fragment) | LF | 1 | 2 | 1 | Y | 119525 |
| tr\|F1N0J5\|F1N0J5_BOVIN | Uncharacterized protein (Fragment) | LF | 1 | 1 | 1 | Y | 133637 |
| tr\|G3N0A8\|G3N0A8_BOVIN | Uncharacterized protein (Fragment) | LF | 0 | 1 | 1 | Y | 445001 |
| tr\|F1MSL9\|F1MSL9_BOVIN | Uncharacterized protein (Fragment) | LF | 3 | 2 | 1 | Y | 174794 |
| tr\|F1MJN7\|F1MJN7_BOVIN | Uncharacterized protein (Fragment) | LF | 1 | 2 | 2 | Y | 227614 |
| tr\|F1MJN1\|F1MJN1_BOVIN | Uncharacterized protein (Fragment) | LF | 1 | 2 | 2 | Y | 242067 |
| tr\|G5E604\|G5E604_BOVIN | Uncharacterized protein (Fragment) | LF | 27 | 2 | 1 | Y | 11058 |
| tr\|G5E622\|G5E622_BOVIN | Uncharacterized protein (Fragment) | LF | 2 | 1 | 1 | Y | 83054 |
| tr\|G3X800\|G3X800_BOVIN | Uncharacterized protein (Fragment) | LF | 2 | 1 | 1 | Y | 69265 |
| tr\|G3X8C8\|G3X8C8_BOVIN | Uncharacterized protein (Fragment) | LF | 4 | 1 | 1 | N | 25182 |
| tr\|G3N0G9\|G3N0G9_BOVIN | Uncharacterized protein (Fragment) | LF | 7 | 1 | 1 | Y | 13626 |
| tr\|F1MVK1\|F1MVK1_BOVIN | Uncharacterized protein (Fragment) | LF | 2 | 3 | 3 | N | 173973 |
| tr\|F1MSZ5\|F1MSZ5_BOVIN | Uncharacterized protein (Fragment) | HF | 2 | 1 | 1 | Y | 87012 |
| tr\|F1MRA8\|F1MRA8_BOVIN | Uncharacterized protein (Fragment) | HF | 8 | 3 | 3 | Y | 62927 |
| tr\|F1MD79\|F1MD79_BOVIN | Uncharacterized protein (Fragment) | HF | 0 | 1 | 0 | N | 130053 |
| tr\|F1MLY7\|F1MLY7_BOVIN | Uncharacterized protein (Fragment) | HF | 1 | 3 | 2 | Y | 299727 |
| tr\|G3MXX3\|G3MXX3_BOVIN | Uncharacterized protein (Fragment) | HF | 1 | 1 | 1 | N | 208923 |
| tr\|F1N544\|F1N544_BOVIN | Uncharacterized protein (Fragment) | HF | 1 | 2 | 1 | N | 226523 |
| tr\|F1N3F6\|F1N3F6_BOVIN | Uncharacterized protein (Fragment) | HF | 1 | 2 | 1 | N | 220106 |
| tr\|F1N734\|F1N734_BOVIN | Uncharacterized protein (Fragment) | HF | 1 | 2 | 2 | Y | 304704 |

| | | | | | | | |
|---|---|---|---|---|---|---|---|
| tr\|F1MS24\|F1MS24_BOVIN | Uncharacterized protein (Fragment) | HF | 1 | 1 | 1 | N | 69174 |
| tr\|G3N309\|G3N309_BOVIN | Uncharacterized protein (Fragment) | HF | 0 | 2 | 1 | Y | 529407 |
| tr\|E1B7E2\|E1B7E2_BOVIN | Uncharacterized protein (Fragment) | HF | 1 | 2 | 1 | N | 221312 |
| tr\|F1MX04\|F1MX04_BOVIN | Uncharacterized protein (Fragment) | HF | 0 | 1 | 0 | N | 178592 |
| tr\|F1MC84\|F1MC84_BOVIN | Uncharacterized protein (Fragment) | HF | 0 | 2 | 1 | N | 503287 |
| tr\|F1N740\|F1N740_BOVIN | Uncharacterized protein (Fragment) | HF | 0 | 2 | 0 | N | 442421 |
| tr\|E1BED7\|E1BED7_BOVIN | Uncharacterized protein (Fragment) | HF | 0 | 3 | 0 | N | 576043 |
| tr\|E1BAI2\|E1BAI2_BOVIN | Uncharacterized protein (Fragment) | HF | 0 | 1 | 1 | N | 115672 |
| tr\|G5E5T3\|G5E5T3_BOVIN | Uncharacterized protein (Fragment) | HF | 1 | 1 | 1 | N | 121682 |
| tr\|G3MW10\|G3MW10_BOVIN | Uncharacterized protein (Fragment) | HF | 1 | 1 | 1 | N | 126823 |
| tr\|G3MZG0\|G3MZG0_BOVIN | Uncharacterized protein (Fragment) | HF | 0 | 2 | 0 | N | 391140 |
| tr\|F1MKJ3\|F1MKJ3_BOVIN | Uncharacterized protein (Fragment) | HF | 0 | 2 | 0 | N | 392941 |
| tr\|E1B805\|E1B805_BOVIN | Uncharacterized protein (Fragment) | HF | 1 | 2 | 1 | Y | 187266 |
| tr\|F1MY32\|F1MY32_BOVIN | Uncharacterized protein (Fragment) | HF | 8 | 1 | 1 | N | 16898 |
| tr\|F1N506\|F1N506_BOVIN | Uncharacterized protein (Fragment) | HF | 2 | 2 | 1 | N | 100655 |
| tr\|F1MLR9\|F1MLR9_BOVIN | Uncharacterized protein (Fragment) | HF | 0 | 1 | 1 | N | 247870 |
| tr\|F1MBK4\|F1MBK4_BOVIN | Uncharacterized protein (Fragment) | HF | 2 | 2 | 0 | N | 60412 |
| tr\|F1N1S8\|F1N1S8_BOVIN | Uncharacterized protein (Fragment) | HF | 2 | 2 | 0 | N | 72696 |
| tr\|F1MJ07\|F1MJ07_BOVIN | Uncharacterized protein (Fragment) | HF | 2 | 2 | 2 | N | 171184 |
| tr\|E1BP38\|E1BP38_BOVIN | Uncharacterized protein (Fragment) | HF | 0 | 2 | 0 | N | 235101 |
| tr\|F1MHA5\|F1MHA5_BOVIN | Uncharacterized protein (Fragment) | HF | 1 | 2 | 1 | Y | 555967 |
| tr\|F1MIL7\|F1MIL7_BOVIN | Uncharacterized protein (Fragment) | HF | 3 | 1 | 1 | N | 29539 |
| tr\|F1MBL6\|F1MBL6_BOVIN | Uncharacterized protein (Fragment) | HF | 0 | 2 | 0 | N | 407888 |
| tr\|F1MRK2\|F1MRK2_BOVIN | Uncharacterized protein (Fragment) | HF | 0 | 2 | 1 | N | 402590 |
| tr\|F1MGT1\|F1MGT1_BOVIN | Uncharacterized protein (Fragment) | HF | 0 | 2 | 0 | N | 1005129 |
| tr\|F1N6T6\|F1N6T6_BOVIN | Uncharacterized protein (Fragment) | HF | 2 | 1 | 1 | Y | 149981 |
| tr\|F1MDH3\|F1MDH3_BOVIN | Uncharacterized protein (Fragment) | HF | 0 | 2 | 0 | N | 270813 |
| tr\|F1MBJ7\|F1MBJ7_BOVIN | Uncharacterized protein (Fragment) | HF | 2 | 3 | 0 | N | 116096 |
| tr\|F1N6I9\|F1N6I9_BOVIN | Uncharacterized protein (Fragment) | HF | 0 | 1 | 1 | Y | 219726 |
| tr\|E1BE64\|E1BE64_BOVIN | Uncharacterized protein (Fragment) | HF | 2 | 3 | 1 | Y | 103705 |
| tr\|G5E6J5\|G5E6J5_BOVIN | Uncharacterized protein (Fragment) | HF | 3 | 2 | 1 | N | 49922 |
| tr\|F1ML28\|F1ML28_BOVIN | Uncharacterized protein (Fragment) | HF | 3 | 2 | 2 | Y | 74213 |
| tr\|F1MJU4\|F1MJU4_BOVIN | Uncharacterized protein (Fragment) | HF | 1 | 1 | 1 | Y | 140767 |
| tr\|G3MXG6\|G3MXG6_BOVIN | Uncharacterized protein (Fragment) | HF | 19 | 1 | 1 | Y | 14759 |

| | | | | | | | |
|---|---|---|---|---|---|---|---|
| tr\|E1BFN5\|E1BFN5_BOVIN | Uncharacterized protein (Fragment)2 | HF | 35 | 22 | 2 | Y | 78385 |
| P10568\|MYO1A_BOVIN | Unconventional myosin-Ia | HF | 1 | 1 | 1 | Y | 118869 |
| Q07130\|UGPA_BOVIN | UTP--glucose-1-phosphate uridylyltransferase | C | 26 | 9 | 9 | N | 56903 |
| tr\|Q2KJ80\|Q2KJ80_BOVIN | Vacuolar protein sorting 33 homolog A (S. cerevisiae) | LF | 1 | 1 | 1 | N | 67508 |
| tr\|A5D9D1\|A5D9D1_BOVIN | Vanin 2 | C | 50 | 24 | 21 | Y | 52621 |
| P63026\|VAMP2_BOVIN | Vesicle-associated membrane protein 2 | LF | 21 | 1 | 1 | N | 12649 |
| tr\|F1MDK1\|F1MDK1_BOVIN | Vesicle-associated membrane protein 2 (Fragment) | LF | 21 | 1 | 1 | N | 12517 |
| Q2KJD2\|VAMP3_BOVIN | Vesicle-associated membrane protein 3 | C | 39 | 5 | 5 | N | 11535 |
| tr\|G3X752\|G3X752_BOVIN | Vesicle-associated membrane protein 3 (Fragment) | C | 40 | 5 | 5 | N | 11404 |
| P81401\|VIP_BOVIN | VIP peptides | C | 77 | 18 | 18 | Y | 19165 |
| tr\|I7CT57\|I7CT57_BOVIN | Vitamin D binding protein | LF | 6 | 2 | 2 | N | 53328 |
| tr\|F1N5M2\|F1N5M2_BOVIN | Vitamin D-binding protein | LF | 6 | 2 | 2 | N | 53356 |
| Q3MHN5\|VTDB_BOVIN | Vitamin D-binding protein | LF | 6 | 2 | 2 | N | 53342 |
| P07224\|PROS_BOVIN | Vitamin K-dependent protein S | LF | 3 | 2 | 2 | Y | 75133 |
| P31404\|VATA_BOVIN | V-type proton ATPase catalytic subunit A | C | 21 | 8 | 8 | Y | 68344 |
| P31408\|VATB2_BOVIN | V-type proton ATPase subunit B brain isoform | HF | 31 | 9 | 9 | Y | 56577 |
| Q32LB7\|VATE2_BOVIN | V-type proton ATPase subunit E 2 | C | 12 | 3 | 3 | N | 26171 |
| P40682\|VAS1_BOVIN | V-type proton ATPase subunit S1 | C | 22 | 6 | 6 | Y | 51781 |
| tr\|Q0VD14\|Q0VD14_BOVIN | WDR45-like | HF | 2 | 1 | 1 | N | 38094 |
| Q9GLM3\|RPGR1_BOVIN | X-linked retinitis pigmentosa GTPase regulator-interacting protein 1 | HF | 1 | 2 | 1 | N | 138433 |
| O97764\|QOR_BOVIN | Zeta-crystallin | C | 24 | 5 | 5 | Y | 35383 |
| tr\|A7E3P5\|A7E3P5_BOVIN | Zinc binding alcohol dehydrogenase domain containing 1 | C | 28 | 4 | 4 | Y | 32091 |
| Q3ZCH5\|ZA2G_BOVIN | Zinc-alpha-2-glycoprotein | C | 61 | 23 | 23 | Y | 33852 |
| tr\|Q32KZ7\|Q32KZ7_BOVIN | Zona pellucida binding protein | LF | 19 | 5 | 5 | Y | 36956 |
